# *Banting Memorial Lecture 2012* Reversing the twin cycles of Type 2 diabetes

**DOI:** 10.1111/dme.12039

**Published:** 2013-02-20

**Authors:** R Taylor

**Affiliations:** Magnetic Resonance Centre, Institute of Cellular Medicine Newcastle UniversityNewcastle upon Tyne, UK

## Abstract

It has become widely accepted that Type 2 diabetes is inevitably life-long, with irreversible and progressive beta cell damage. However, the restoration of normal glucose metabolism within days after bariatric surgery in the majority of people with Type 2 diabetes disproves this concept. There is now no doubt that this reversal of diabetes depends upon the sudden and profound decrease in food intake, and does not relate to any direct surgical effect. The Counterpoint study demonstrated that normal glucose levels and normal beta cell function could be restored by a very low calorie diet alone. Novel magnetic resonance methods were applied to measure intra-organ fat. The results showed two different time courses: a) resolution of hepatic insulin sensitivity within days along with a rapid fall in liver fat and normalisation of fasting glucose levels; and b) return of normal beta cell insulin secretion over weeks in step with a fall in pancreas fat. Now that it has been possible to observe the pathophysiological events during reversal of Type 2 diabetes, the reverse time course of events which determine the onset of the condition can be identified. The twin cycle hypothesis postulates that chronic calorie excess leads to accumulation of liver fat with eventual spill over into the pancreas. These self-reinforcing cycles between liver and pancreas eventually cause metabolic inhibition of insulin secretion after meals and onset of hyperglycaemia. It is now clear that Type 2 diabetes is a reversible condition of intra-organ fat excess to which some people are more susceptible than others.

## Introduction

What is the basic nature of Type 2 diabetes? What causes it? It is associated with obesity, and both insulin resistance and a β-cell defect are involved. After diagnosis blood glucose levels rise steadily whether or not treatment is intensive 1. By 10 years after diagnosis, the rate of rise in HbA_1c_ decreases, because by that stage 50% of all people with Type 2 diabetes are on insulin therapy. This steady deterioration has been observed in many studies and underpins the belief that Type 2 diabetes is an inexorably progressive disease. It is widely acknowledged as a lifelong condition and, in order to maximize coping, those with the disease are advised to come to terms with the notion that they have an incurable condition 2.

## What causes the deterioration in control?

The major pathophysiological change underlying the steady worsening of blood glucose control is a steady decrease in β-cell function, as shown by modelling β-cell function from the UK Prospective Diabetes Study (UKPDS) data set 3. Indeed, at the time of diagnosis, it is already down to 50% of normal. Then it deteriorates in a depressingly linear fashion. The rate of decline in glucose tolerance is strongly related to the loss of β-cell function, whilst insulin resistance in muscle changes little 4,5. This mirrors observations on populations with high incidence of Type 2 diabetes, in which transition from hyperinsulinaemic normal glucose tolerance to overt diabetes involved a further loss of acute β-cell competence 6,7.

**FIGURE 1:**
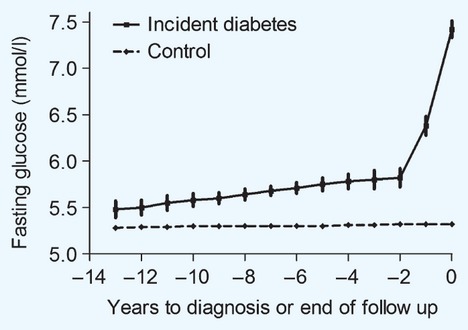
Change in fasting plasma glucose during the 13 years prior to onset of Type 2 diabetes. These data from the Whitehall II study demonstrate the elevation of plasma glucose within the normal range for many years, but a sudden breakdown of control mechanisms around 2 years prior to diagnosis. Data replotted from Tabak *et al*. (2009), with permission from Elsevier 8.

Direct observation of the 13-year period leading up to diagnosis of Type 2 diabetes has recently provided insight into the time course of changes leading to diagnosis. The Whitehall II Study has shown that plasma glucose levels are very slightly elevated years before diagnosis, although well within the normal range, but that the onset of distinct hyperglycaemia occurs over only 2 years (Fig. 1) 8. What could underlie this relatively rapid failure of insulin secretion? Does this reflect a process such as amyloid deposition or oxidative stress leading to β-cell death? Postulating such a mechanism would suggest that Type 2 diabetes requires the coincidence of two separate disease processes—one to cause β-cell dysfunction and one to cause insulin resistance. This seems overly complicated for such a common disease.

## Background physiology

### β-cell

**FIGURE 2:**
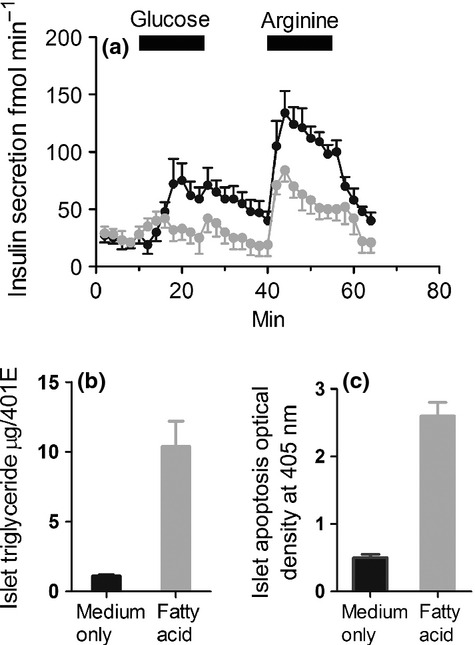
(a) Rates of insulin secretion by islets from pre-diabetic male Zucker diabetic fatty (ZDF) rats when *in vitro* glucose concentration is raised from 3 to 23 mmol/l and when arginine is added at 20 mmol/l. Pre-incubated at 3 mmol/l glucose only (black line); pre-incubated for 7 days with 2 mmol/l oleic and palmitic acid (grey line). In susceptible islets, excess provision of alternative fuel prevents ATP production from glucose or arginine, and this causes decreased insulin secretion. Data replotted from Lee *et al*. (1994) with permission from Dr Roger H. Unger [9]. (b, c) Exposure of human islet to fatty acid causes an increase in intra-islet fat storage (b) and a marker of apoptosis (c). Data replotted from Lalloyer *et al*. (2006) with permission from American Diabetes Association 10.

*In vitro* observations on the β-cell insulin secretion are helpful. Figure 2 shows the increase in rate of insulin production by islets exposed to a sudden increase in either glucose or arginine availability. Arginine is an amino acid that is readily taken up by β-cells. The increase in insulin secretion in response to both substances happens because supply of any metabolic fuel will cause the β-cells to make adenosine-5'-triphosphate (ATP). In turn, an increase in intracellular ATP activates the process of insulin secretion. But if the β-cells have previously been exposed to unremarkable concentrations of a different nutrient—fatty acid—then ATP production is chronically increased. The β-cells no longer secrete insulin normally in response to glucose, and only grudgingly in response to arginine (Fig. 2a). The β-cells simply have too much ATP already because of energy from fat, and making more from glucose is unnecessary and unwelcome. This causes them to fail to respond normally to glucose. Islets from animals not genetically predisposed to diabetes can withstand the excess fat supply and retain glucose-mediated insulin secretion 9.

Studies on human islets have shown that exposure to a low concentration of fatty acids brings about avid uptake of fat (Fig. 2b and c) 10. The presence of excess fat increases the rate of programmed cell death or apoptosis. As a low rate of β-cell turnover may continue throughout human life, β-cell number will fall if the rate of apoptosis is increased. Post-mortem studies show that β-cell number at diagnosis of Type 2 diabetes is around 50% and decreases to around 20% after many years 11,12. Fatty acids have been shown to inhibit β-cell proliferation *in vitro* by induction of the cell cycle inhibitors p16 and p18, especially when glucose concentration is increased 13. In the Zucker diabetic fatty (ZDF) rat, a genetic model of spontaneous Type 2 diabetes, a rapid increase in pancreatic fat precedes the onset of hyperglycaemia 9. It is particularly noteworthy that diabetes in this genetic model is completely preventable by restriction of food intake 14. This illustrates very well the interaction between genetic susceptibility and environmental factors in the aetiology of Type 2 diabetes.

### Liver

In liver, all triglyceride is stored intracellularly, unlike in muscle. The sensitivity of the liver to insulin is strongly related to the intra-hepatocellular triglyceride content 15. Decreasing the fat content of liver can improve insulin suppression of glucose production and improve fasting plasma glucose 16,17. In passing, it should be noted that fasting glucose concentration is entirely determined by glucose production from the liver.

How does fat accumulate in liver? Storage of liver fat can only occur when the total daily calorie intake exceeds expenditure day after day, and year after year. During any one period of time, if more calories are ingested than metabolized then any fat excess is stored either subcutaneously, viscerally or in the liver. But any excess carbohydrate cannot be stored once the glycogen depots are full. If more glucose is ingested than can be oxidized for energy or stored as glycogen, it has to be turned into fat by the process of *de novo* lipogenesis. This process only happens in the liver in humans, and triglyceride synthesized *in situ* is particularly likely to be stored in hepatocytes rather than exported for safe storage in subcutaneous adipose tissue. The newly synthesized fat has three possible fates: it can be oxidized for energy; exported as VLDL in the plasma to be delivered to other tissues or it can be stored in a rather full liver. As *de novo* lipogenesis is stimulated by insulin, those people who are relatively insulin resistant in muscle—and who therefore have a raised plasma insulin level—are especially likely to accumulate fat in the liver. This could explain the reason why muscle insulin resistance is the first detectable signal of risk for Type 2 diabetes 18,19.

One recent study has shown very clearly the effect of consistent calorie excess. In the Candy study, volunteers were asked to eat a bag of sweets, drink a 300-ml bottle of Pepsi and 30 ml of fruit juice each day in addition to their usual food. This sucrose overfeeding for 3 weeks brought about a 30% increase in liver fat content 20. This was associated with a 30% rise in serum alanine aminotransferase (ALT), indicating the associated metabolic stress on hepatocytes.

**FIGURE 3:**
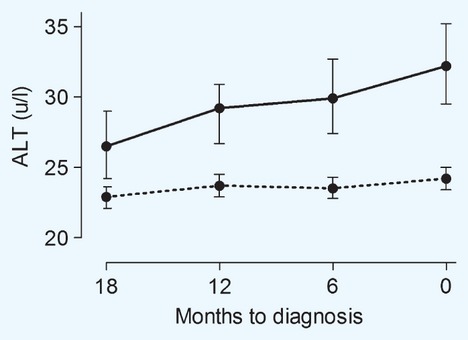
During the 18 months prior to diagnosis of Type 2 diabetes, serum ALT rises steadily (solid line). This is likely to reflect the steadily increasing burden of fat within hepatocytes. In any one individual, this change may go unnoticed as for most people it remains within the range of normal. Serum ALT is also shown for the larger group who did not develop diabetes (dotted line). Data replotted from Sattar *et al*. (2007) with permission from American Diabetes Association 23.

The direct relevance of this to the onset of Type 2 diabetes was elegantly demonstrated by Sattar and colleagues. The West of Scotland Coronary Prevention Study group of 6595 men were followed up for 15 years, and naturally some developed diabetes. Stored plasma samples were analysed to allow retrospective reconstruction of what happened to liver enzymes in the majority who did not develop diabetes, and in those who were about to develop diabetes. Figure 3 shows the rise in ALT, which precedes diagnosis of diabetes. This is well within the normal range, and it is unlikely to be detected in any one individual. Hepatic fat is building up and the process of Type 2 diabetes is underway. Before diagnosis of Type 2 diabetes, there is a long silent scream from the liver.

## Surgical reversal of Type 2 diabetes

We have known for over 30 years that most people show normalization of blood glucose after gastric bypass surgery. However, data from Guidone and colleagues were striking 38. Blood glucose levels normalized within 7 days of biliopancreatic diversion. What has been done to the natural history of this chronic condition? It has become widely believed that surgery produces normoglycaemia via a direct effect upon the incretin hormones. This simply cannot be the primary cause of the early change in fasting plasma glucose, as these secondary regulatory hormones fall well short of such capability.

There is an obvious and cataclysmic change in metabolism at the time of surgery. The group studied by Guidone had an average weight of 150 kg (BMI 54 kg/m^2^) and must therefore have been eating at least 3200 kilocalories per day merely to maintain their body mass. They were suddenly prevented from doing so at the time of surgery. The immediate consequence is therefore negative calorie balance, and the body must suddenly draw upon its reserves. Instead of the fatty acid intermediaries that inhibit glucose metabolism being left to accumulate in the cytoplasm 21, they are urgently required for oxidation in mitochondria. Diacylglycerol, the product of removing one of the three fatty acids from triacylglycerol (i.e. triglyceride), rapidly decreases in concentration and suddenly the cell has a normal choice of fuel—either glucose or fat depending upon needs. The likely sequence of events has been drawn together as the Twin Cycle Hypothesis, which describes the reversible aetiology of Type 2 diabetes 22.

During chronic positive calorie balance, any excess carbohydrate must undergo *de novo* lipogenesis, and this particularly promotes fat accumulation in the liver. As insulin stimulates *de novo* lipogenesis, individuals with a degree of insulin resistance (determined by family or lifestyle factors) will accumulate liver fat more readily than others because of the higher plasma insulin levels. The increased liver fat, signalled by rising serum ALT levels 23 in turn will cause relative resistance to insulin suppression of hepatic glucose production. Over many years the resulting hyperinsulinemia will increase further the conversion of excess calories into liver fat. A vicious cycle of hyperinsulinaemia and increased liver fat will become established. Fatty liver leads to increased export of VLDL triacylglycerol 24, which will increase fat delivery to the islets, with excess fatty acid availability impairing the acute insulin secretion in response to ingested food. Eventually the fatty acid and glucose inhibitory effects on the islets will reach a trigger level, precipitating clinical diabetes. Post-bariatric surgery, the whole mechanism could be thrown into reverse because of profound negative calorie balance. This is a testable hypothesis.

## Testing the Twin Cycle Hypothesis

The Counterpoint study (Counteracting Pancreatic Inhibition by Triglyceride) aimed to induce negative calorie balance using a very low calorie diet—about one quarter of an average person's daily food intake 25. Before and during an 8-week period, gold standard tests of β-cell function and insulin sensitivity in liver and muscle were carried out. But in order to measure the postulated culprit, a new method was devised for quantitation of fat in liver and pancreas.

**FIGURE 4:**
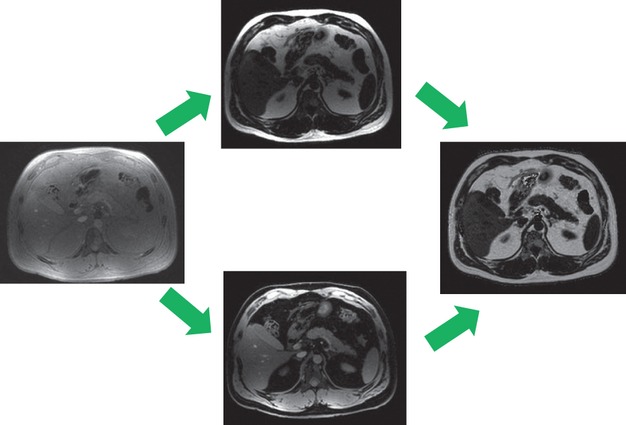
On the left is a standard image of the abdomen. As the atomic nuclei of protons in fat and water resonate at slightly different frequencies, it is possible to separate the signals from each. Separate images of fat (upper) and water (lower) can be derived. Then, for every single pixel the percentage of fat can be calculated and plotted as an image (right). This allows averaging of the percentage of fat for each pixel definitely within the pancreas and, as the pancreas is identified after acquisition of the image, this can be precisely determined. The same process can be applied to liver, avoiding major vessels.

Figure 4 outlines how the magnetic resonance method works. Previous methods required an acquisition time of approximately 20 min, and were not precise because of respiratory movements causing visceral fat to move in and out of the volume where pancreas was expected to be. This was avoided using a new approach in which the information was acquired in a single 10-s breath hold, and the volume of tissue to be examined was defined precisely. As the signal comes from hydrogen atom nuclei, and as these behave differently when in water molecules compared with fat molecules, it was possible to measure the percentage of fat in each pixel and to express these values as an average for the organ. Both pancreas and liver fat could be measured, with excellent Bland–Altman reproducibility coefficients of 0.9 and 0.5, respectively.

The very low calorie diet was given as three sachets per day of liquid meal replacement [Optifast (Nestle UK Ltd, Croydon, Surrey, UK); total 600 kcalories] plus non-starchy vegetables or salad. Although monotony was the main problem of this diet, it was strikingly effective. In the group of people with Type 2 diabetes, mean body weight decreased by 3.9 kg in the first week (60% of this as fat) and a total of 15.3 kg over the 8-week period. This is similar to weight loss after gastric bypass, and the conditions had been established to allow testing of the hypothesis.

**FIGURE 5:**
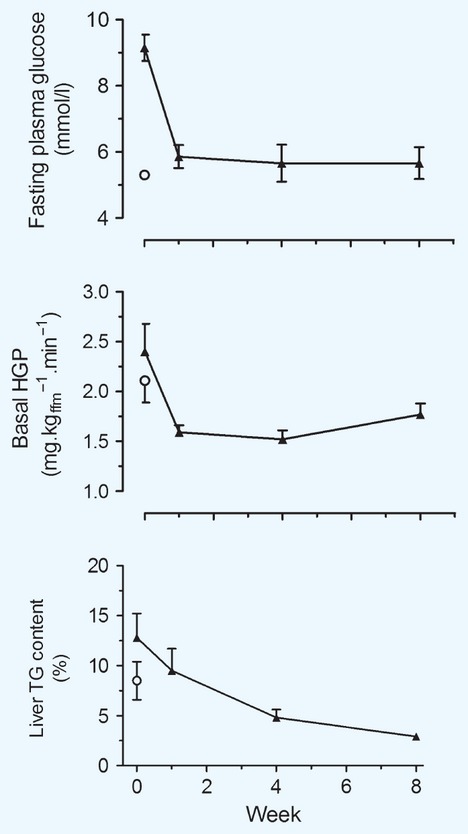
Effect of a very low calorie diet in Type 2 diabetes on fasting plasma glucose (upper panel), basal rate of hepatic glucose production (HGP) (middle panel) and hepatic triacylglycerol (TG) content (lower panel). For comparison, data from a matched control group without diabetes are shown as open circles. Reproduced from Lim *et al*. (2011) 25.

**FIGURE 6:**
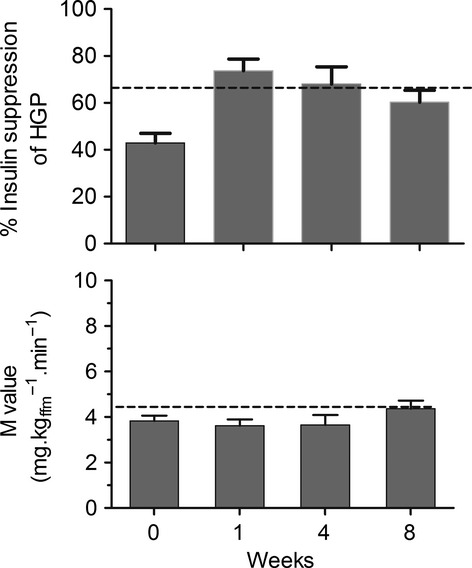
After induction of sudden negative calorie balance, there was an immediate improvement in insulin sensitivity of liver (upper panel), in sharp contrast to no change in muscle (lower panel). Insulin sensitivity for liver is shown as the degree of suppression of hepatic glucose output (HGP) during an insulin clamp (insulin infusion rate 40 mU m^−2^ min^−1^) and for muscle as clamp glucose disposal rate. The dotted lines indicate the mean values for the normoglycaemic control group. Data replotted from Lim et al. (2011) 25.

A dramatic fall in plasma glucose was observed, comparable with that following gastric bypass surgery. In the first 7 days, fasting plasma glucose fell from 9.2 mmol/l to normal (Fig. 5). It remained normal. One of the most important actions of insulin is to keep the hepatic production of glucose under control. The liver in turn has the job of producing glucose constantly. A person only wakes up in the morning because their liver has faithfully been making the glucose needed by the brain, all night long. But high insulin levels should suppress this after breakfast when food-derived glucose will take over the supply—providing that the liver is fully sensitive to insulin. The matched normoglycaemic control subjects suppressed fasting glucose production during insulin infusion by nearly 70%. And the subjects with Type 2 diabetes at baseline had very insulin-resistant livers—with suppression by only 43% at baseline. This is also the reason why fasting plasma glucose is raised in diabetes—the liver glucose production is relatively unsuppressed. But within 7 days, the insulin sensitivity of the liver had normalized in the group with diabetes (Fig. 5 & 6). Liver fat fell by 30% during the first 7 days of negative energy balance. In view of the known relationship between intra-hepatocellular fat and hepatic insulin sensitivity 16,17, this would determine the normalization of fasting hepatic glucose production. The first part of the Twin Cycle Hypothesis has been confirmed.

There was no change in muscle insulin sensitivity during the diet, and it is obvious that it played no part in the very rapid achievement of normal plasma glucose. This has previously been demonstrated during more modest calorie restriction 16. Insulin resistance in muscle would appear to act as a long-term promotor of the diabetic state (Fig. 6) rather than as a factor causing ambient hyperglycaemia.

**FIGURE 7:**
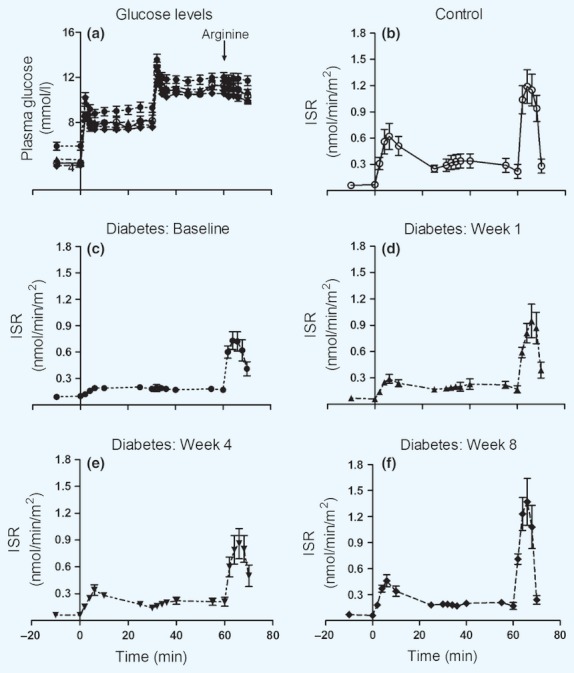
The Stepped Insulin Secretion Test was carried out in test subjects and control subjects by stepwise elevation of plasma glucose, then administration of a bolus of arginine. (a) Achieved plasma glucose levels in each group. (b) Insulin section rates (ISR) in a matched control group. c) Diabetes group at baseline. (d) Diabetes group at 1 week of diet. (e) Diabetes group at 4 weeks. (f) Diabetes group at 8 weeks. It can be seen that both first-phase response and maximal (arginine-stimulated) rates of insulin secretion return to normal. Reproduced from Lim *et al*. (2011) 25.

To quantify precisely the changes in β-cell function, it was vital to use a gold standard method that directly measured both first-phase and maximal insulin secretion rates (Fig. 7). During the Stepped Insulin Secretion Test (SIST), plasma glucose was raised by 2.8 mm by glucose intravenous infusion, then kept steady above basal level. It was then raised by another 2.8 mmol/l and an intravenous bolus arginine was given to maximally stimulate insulin secretion. At baseline, the first-phase insulin response was absent and the maximal peak of insulin secretion was less than 60% of control values, as would be expected in Type 2 diabetes. Week by week during the Counterpoint study, the insulin response increased so that, after 8 weeks of diet, the first-phase response was within the normal range and the maximal insulin secretion rate was numerically but not significantly greater than that of the control subjects. The β-cells had woken up! This has never been demonstrated before. Clearly, the β-cells are not permanently damaged in Type 2 diabetes, but are merely metabolically inhibited.

**FIGURE 8:**
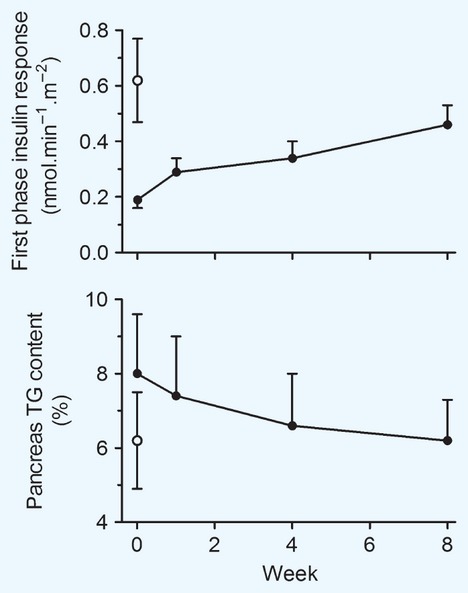
During the 8 weeks of the hypocaloric diet there were steady and reciprocal changes in first-phase insulin secretion (upper panel) and pancreas fat content (lower panel). Values for the weight-matched control group without diabetes are shown as open circles. Reproduced from Lim *et al*. (2011) 25.

Change in pancreas fat content and change in first-phase insulin response during the 8-week study period is shown in Fig. 8. The slow steady decrease in pancreas fat content was mirrored by the slow steady improvement in first-phase response. In view of the information presented above, the relationship is likely to be causative.

## Further observations

Because of the remarkable extent of blood glucose improvement during the 8-week study, additional ethics permission was obtained to carry out an oral glucose tolerance test 12 weeks after completing the diet. Subjects had no formal dietary advice or follow-up, but were provided with general advice about continuing calorie restriction. At the time of follow-up oral glucose tolerance test, weight increase by 3.1 ± 1.0 kg had occurred. HbA_1c_ remained similar (44 ± 1 vs. 42 ± 2 mmol/mol; 6.2 ± 0.1 vs. 6.0 ± 0.2%) and fasting plasma glucose was 6.1 ± 0.2 mmol/l (compared with 5.7 ± 0.5 at week 8, and 9.2 ± 0.4 mmol/l at baseline). Despite the weight gain, the tests confirmed normal glucose tolerance in three subjects, impaired glucose tolerance in four subjects and return of diabetes in only three of the 10 subjects able to be retested. Further research is required to establish whether the reversal of Type 2 diabetes continues long term if weight gain is avoided. Certainly the first patient I personally advised about substantial weight loss to reverse diabetes presented 8 years ago, just when the role of fat in the liver was coming into focus, and remains normoglycaemic 8 years later.

After publication of the Counterpoint Study, word about reversal of Type 2 diabetes spread rapidly. Over 1000 emails and letters were received from people with diabetes in the subsequent 6 months. Interest was worldwide, although the majority of enquiries were from the UK. The emails all told the same story. These people detested their diabetes and wanted to regain their health. All the emails were answered, and information on how diabetes can be reversed was placed on the Newcastle Magnetic Resonance Centre website. A second wave of emails from the same people later reported substantial weight loss and over half achieved reversal of diabetes. There was an average weight loss of 15 kg, and average blood glucose normalized 26. A proportion had been previously on insulin therapy. At the stage of considering major weight loss to reverse their diabetes, approximately half of this cohort of 77 people had been strongly discouraged by doctor or dietician, but they had gone ahead. The other half had been well supported and some doctors and dieticians requested information directly from the research team.

Certainly, the people who emailed were not representative of the average practice population of people with Type 2 diabetes, the majority of whom may not wish to change their lifestyle or feel unable to do this. The respondents were clearly a distinct and important subgroup who are likely to benefit from very different management. A label is required to allow doctors to recognize and appropriately manage this subgroup who were willing to do anything to get rid of their diabetes. These are the Health Motivated. At the time of diagnosis, the Health-Motivated individuals will benefit from being advised that they are likely to be able to reverse their diabetes completely by achieving weight loss equivalent to 15–20% of body weight. Only if subsequent outcomes show that they are not sufficiently strongly motivated should the routine guidelines for managing Type 2 diabetes be rolled out. As an association (Diabetes UK) we promote self-care. Type 2 diabetes can be reversed at home.

## Questions to be answered

The recent observations raise several major questions.

Is long-standing Type 2 diabetes reversible? The Counterpoint study involved only people with short duration of diabetes. But we now know of many people reversing long-duration Type 2 diabetes (one person after 28 years) following either a hypocaloric diet or bariatric surgery 26,27.

Is diabetes with normal BMI reversible? Yes: if you show me a person of normal BMI who has classical Type 2 diabetes, I shall show you a relatively fatty liver. This is most evident in people of South Asian ethnicity.

Will diabetes come back? No, provided weight is kept down below an individual's personal fat threshold to trigger Type 2 diabetes.

What is the best advice to prevent weight regain? Certainly it must be a eucaloric diet with increased daily physical exercise, although possible benefits of specific foodstuffs require to be formally tested. This is a story just beginning.

## Path to the Twin Cycle Hypothesis

The path to this hypothesis has journeyed through adipose tissue, muscle, liver and now has led to the central organ of Type 2 diabetes—the pancreas. George Alberti supervised my research fellowship and has been integral to the success of the entire expedition. We established that the insulin receptor on human adipocytes did not reflect that on circulating monocytes, contrary to beliefs at the time 28. Mary Argyraki, my first research fellow, demonstrated that fatty acids could induce insulin resistance in human muscle strips *in vitro* 29. Andrew Johnson took this *in vivo* and showed that intralipid infusion caused insulin resistance by substrate competition, not by affecting the process of insulin signalling in muscle 30. David Broughton demonstrated that ageing had nothing to do with insulin resistance if physical activity was matched between older and younger groups 31. Then, as a result of a 1-year sabbatical I spent working with Jerry Shulman at Yale University, we were able to prove that the magnetic resonance method was accurate in comparison with muscle biopsy plus biochemical analysis 32,33. This was a critical step in applying magnetic resonance to study human metabolism. After transplanting the methods back to the UK, Peter Carey was able to describe what happened during everyday eating and demonstrated that normal storage of food as glycogen in muscle was almost absent in Type 2 diabetes 34. But muscle did not explain it all. Parag Singhal showed that the abnormally high production of glucose from the liver was not normally suppressed in diabetes 35. Balasubramanian Ravikumar then demonstrated the tight relationship *in vivo* between amount of liver fat and insulin sensitivity of the liver 17. Mike Trenell moved mountains to transform our ability to influence everyday ordinary activity for metabolic benefit and to measure outcomes 36. Ana Jovanovic showed how our new metabolic understanding could be transformed into simple means of improving control without using drugs 37. A prior snack encourages muscle to store glycogen after a meal, and lowers post-meal plasma glucose. And, most recently, Ee Lin Lim deserves enormous credit for carrying out the Counterpoint study 25. But the powerhouse of this research has been the magnetic resonance physics team who devised the means of probing the inner secrets of the pancreas.

## Summary

**FIGURE 9:**
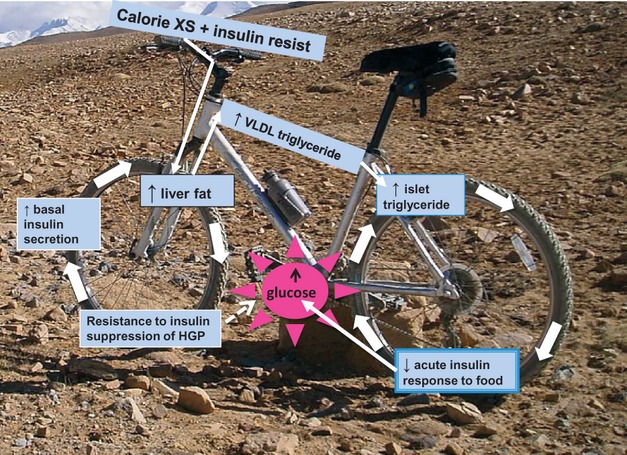
This bicycle is controlled by handlebars representing chronic, excess (XS) calorie intake in the presence of muscle insulin resistance. Raised plasma insulin levels will expedite chronic excess calorie storage from carbohydrate via *de novo* lipogenesis, and hence promote liver fat storage. This will cause the liver to become relatively resistant to insulin and a small increase in plasma glucose will occur. In turn, insulin secretion will increase to control plasma glucose down. The further increased insulin levels will bring about a self-reinforcing cycle. Excess fat will result in increased export of VLDL triglyceride from the liver, uptake by islets and inhibition of meal insulin secretion. At a personal threshold, the pancreas fat becomes too great a load and plasma glucose levels will then rise relatively rapidly. HGP, hepatic glucose production.

The Twin Cycle Hypothesis has come of age. Figure 9 illustrates the pathophysiological steps whereby *de novo* lipogenesis increases liver fat, and spillover of VLDL triglyceride will increase islet fat exposure. At a personal fat threshold, the β-cells fail to respond adequately to food and plasma glucose levels become abnormal. For the person riding this bi-cycle, a glance at the front wheel, whilst hurtling down the hill of life, may provide a shock. For it is going the wrong way round. If that person adjusts the handlebars to remove the driving factor of positive calorie balance, the cycles can be reversed.

Health-Motivated people can reverse their Type 2 diabetes completely and maintain long-term normoglycaemia. The recent research was designed to illuminate the basic nature of Type 2 diabetes, and therapeutic studies must follow.

It is now clear that Type 2 diabetes is a simple condition of fat excess to which some people are more susceptible than others.

## Supporting Information

**Slides 1.** PowerPoint presentation.
