# Supplementary material for: Banting Memorial Lecture 2012 Reversing the twin cycles of Type 2 diabetes
Source: Diabet Med. 2013 Feb 20;30(3):267–75. doi: 10.1111/dme.12039 (PMC3593165; doi:10.1111/dme.12039)
Supplement: Supplementary file 1 [file dme0030-0267-SD1.pptx]

## Slide 1
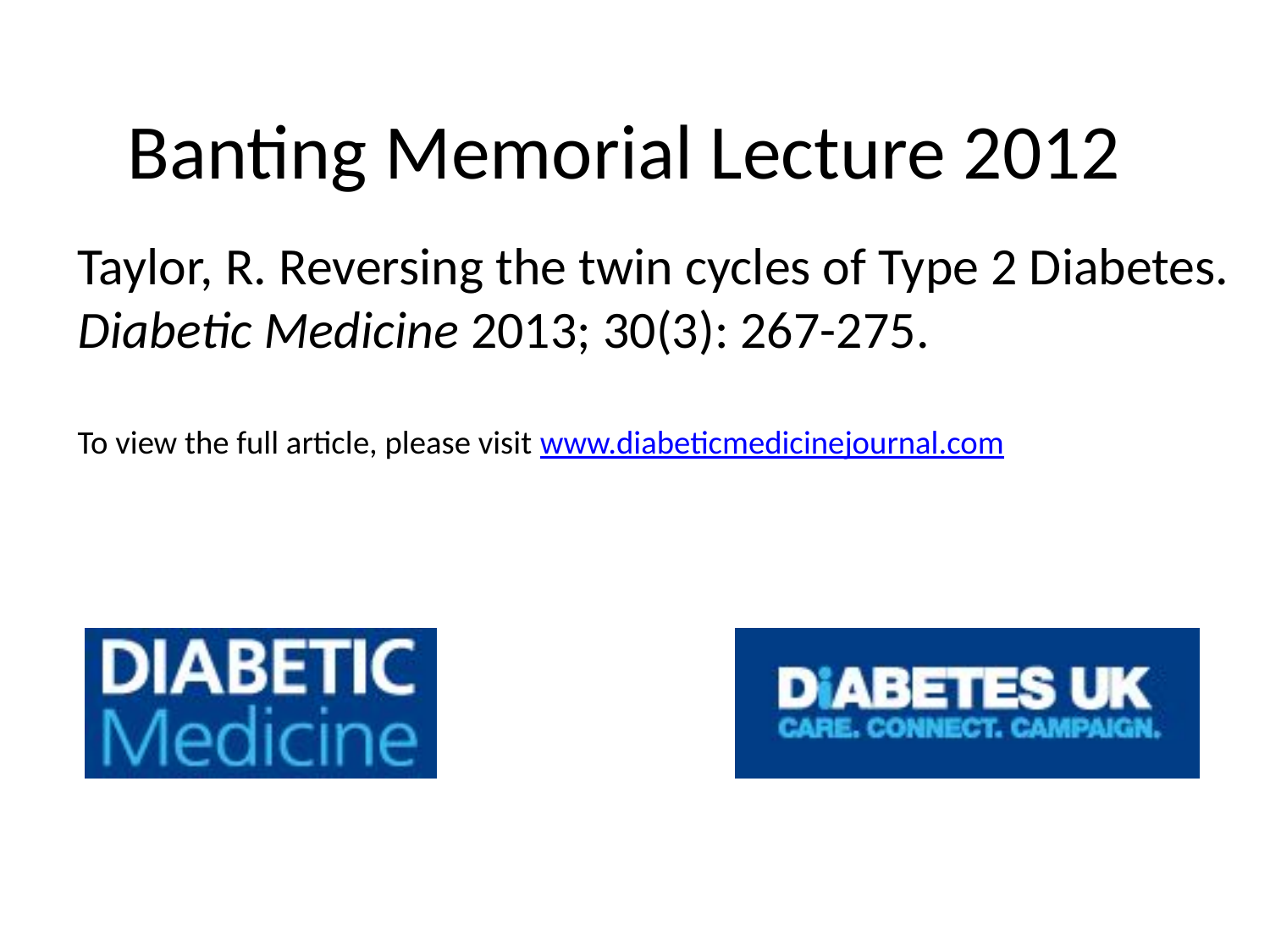

# Banting Memorial Lecture 2012
Taylor, R. Reversing the twin cycles of Type 2 Diabetes. Diabetic Medicine 2013; 30(3): 267-275.
To view the full article, please visit www.diabeticmedicinejournal.com

## Slide 2
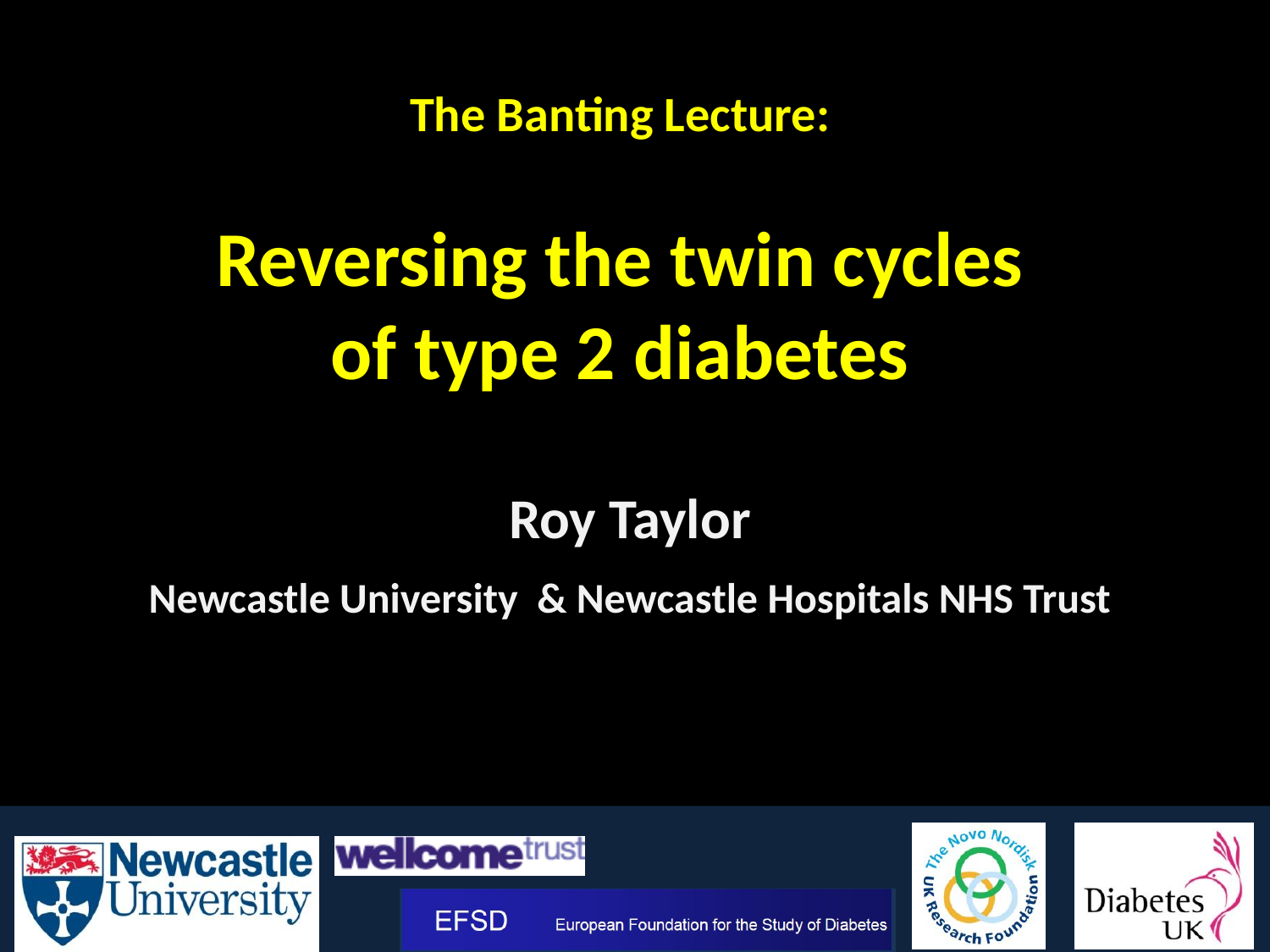

The Banting Lecture:
Reversing the twin cycles of type 2 diabetes
Roy Taylor
Newcastle University & Newcastle Hospitals NHS Trust

## Slide 3
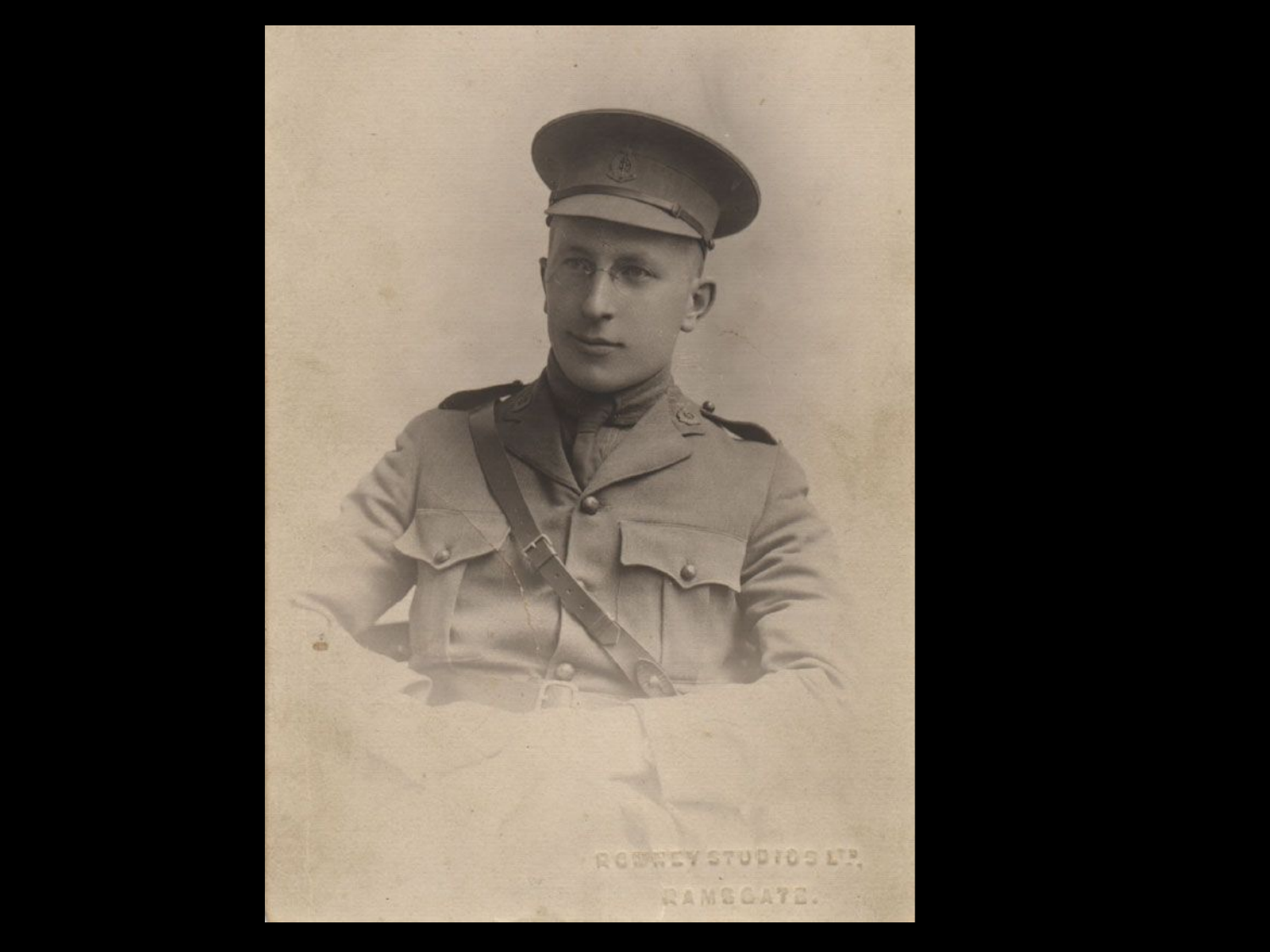

## Slide 4
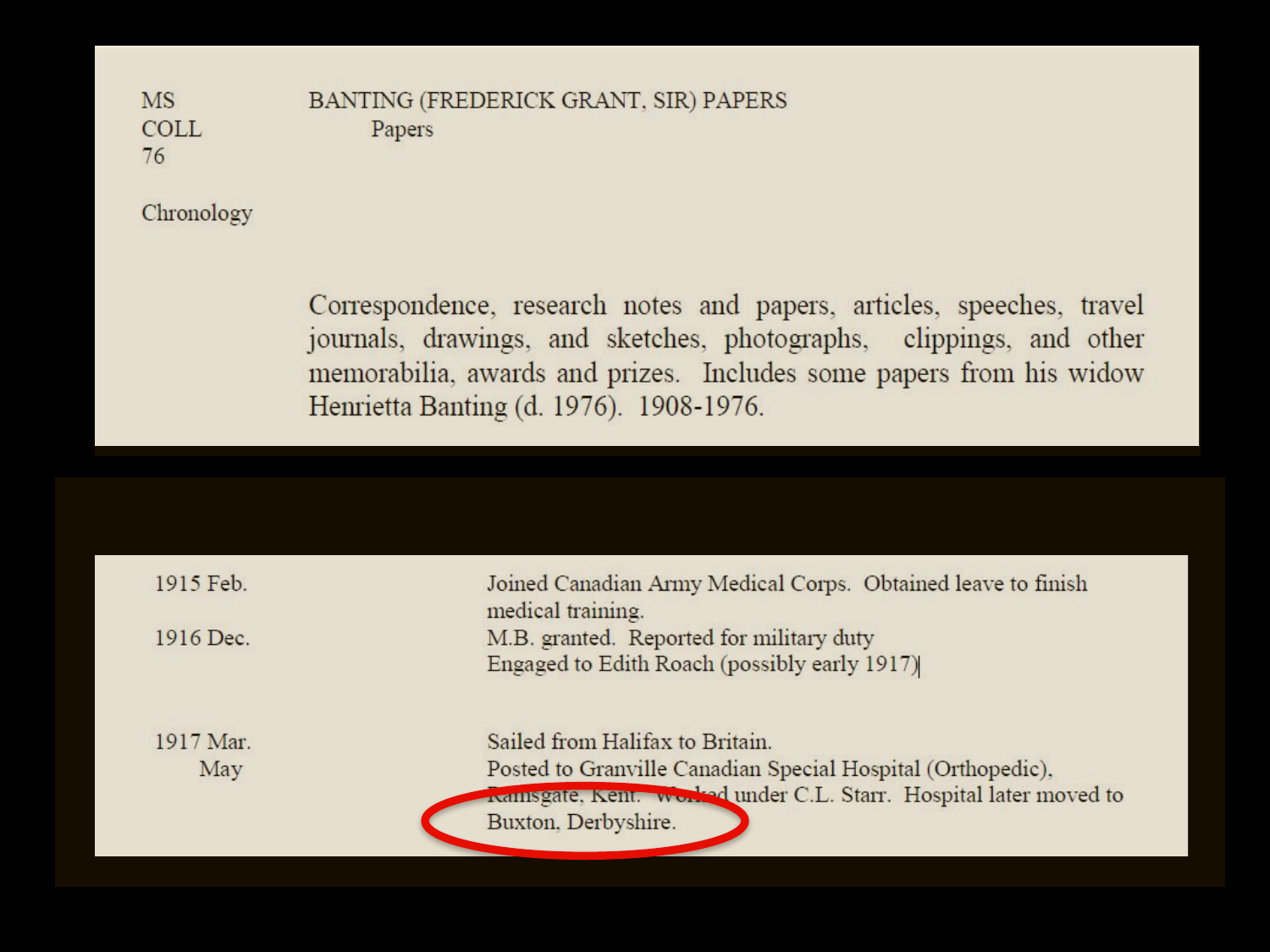

## Slide 5
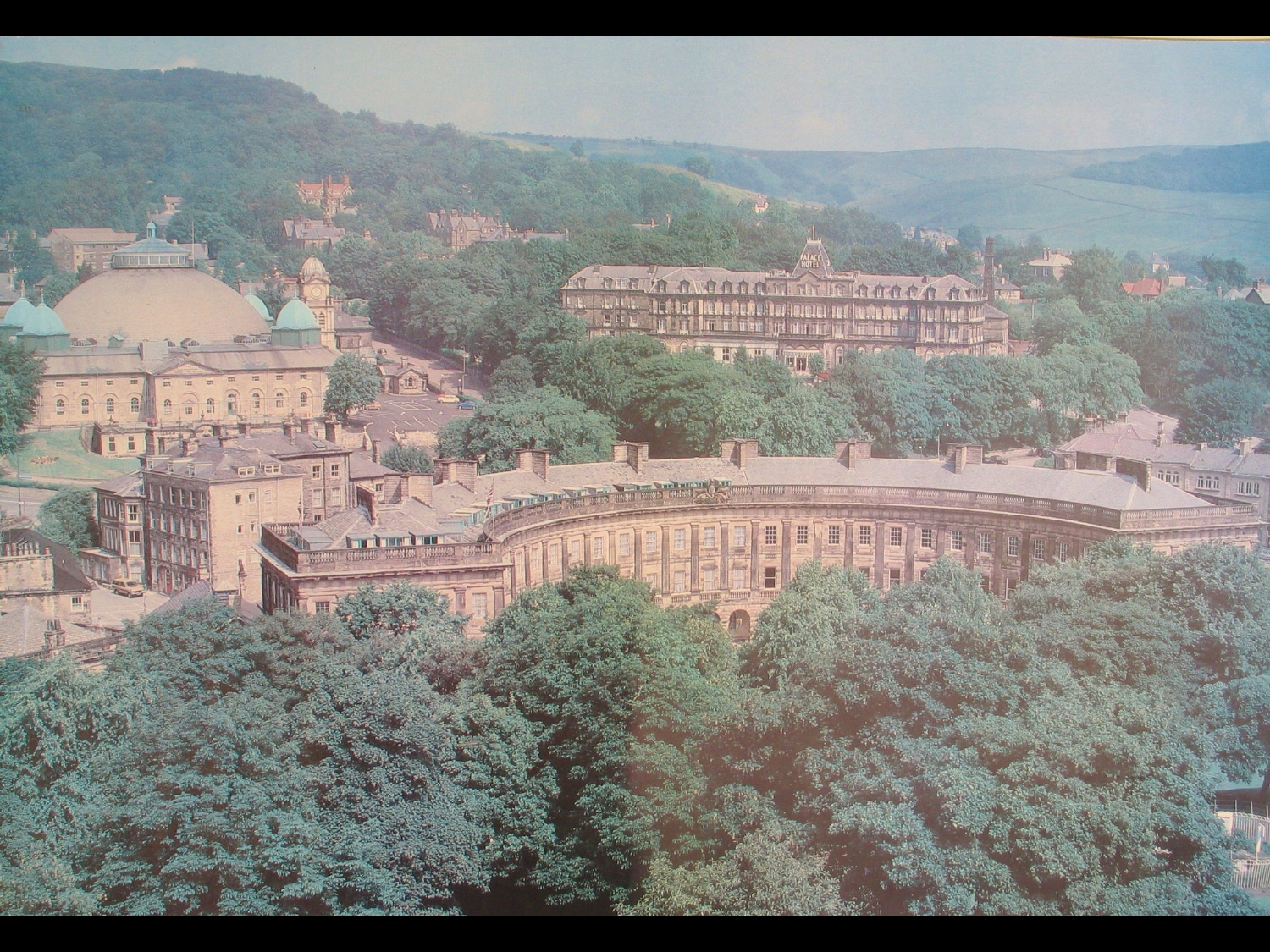

## Slide 6
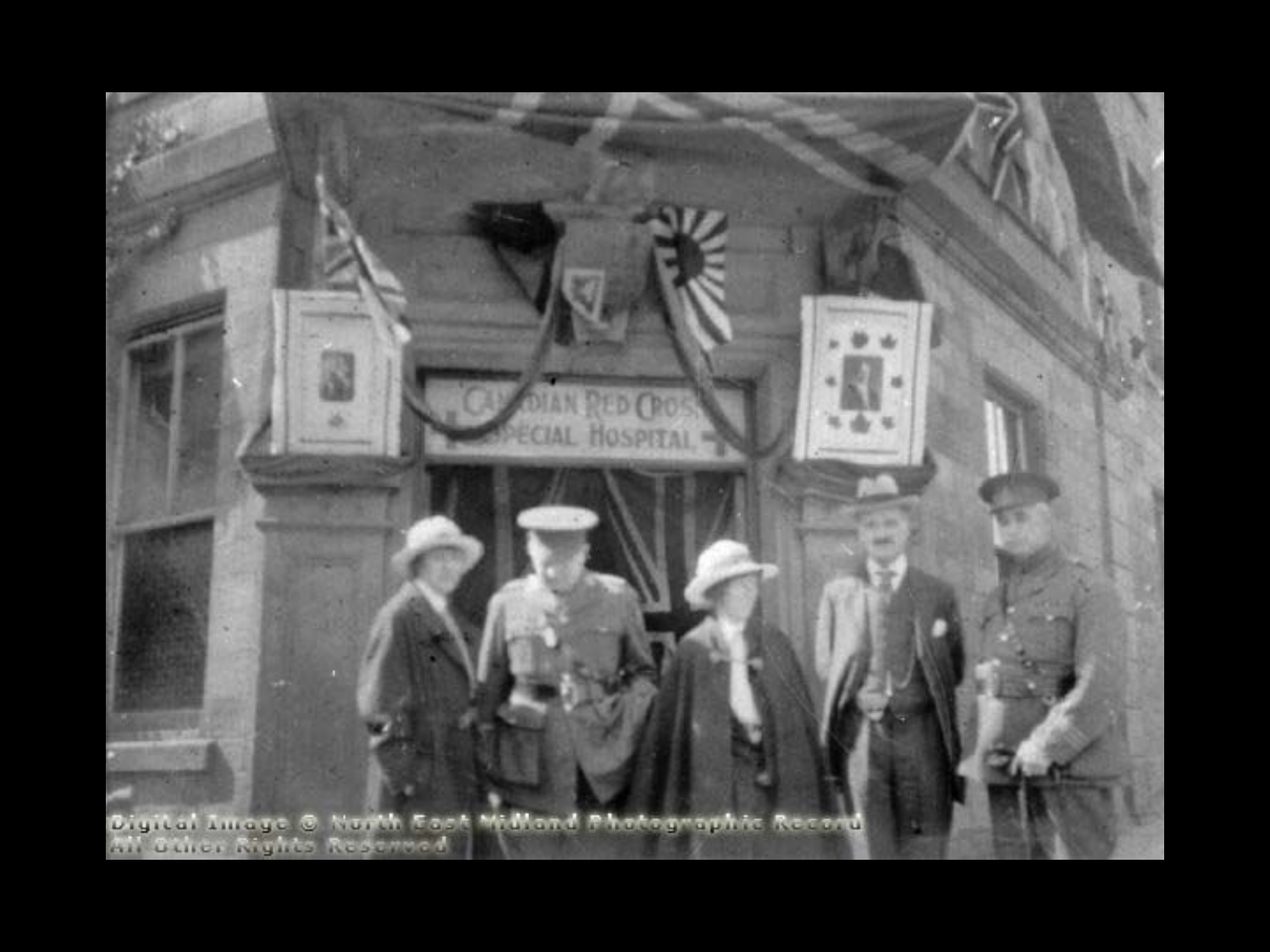

## Slide 7
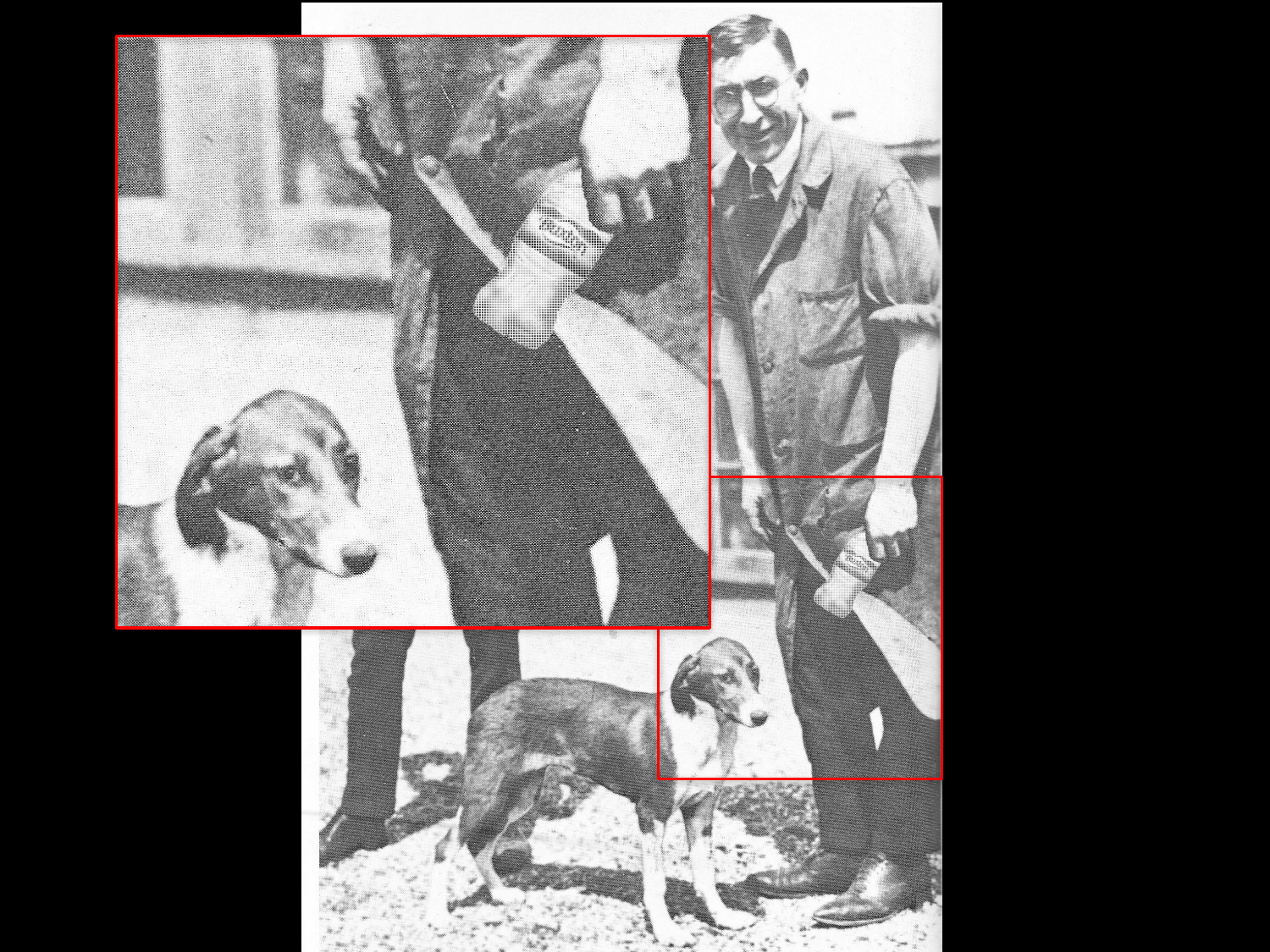

## Slide 8
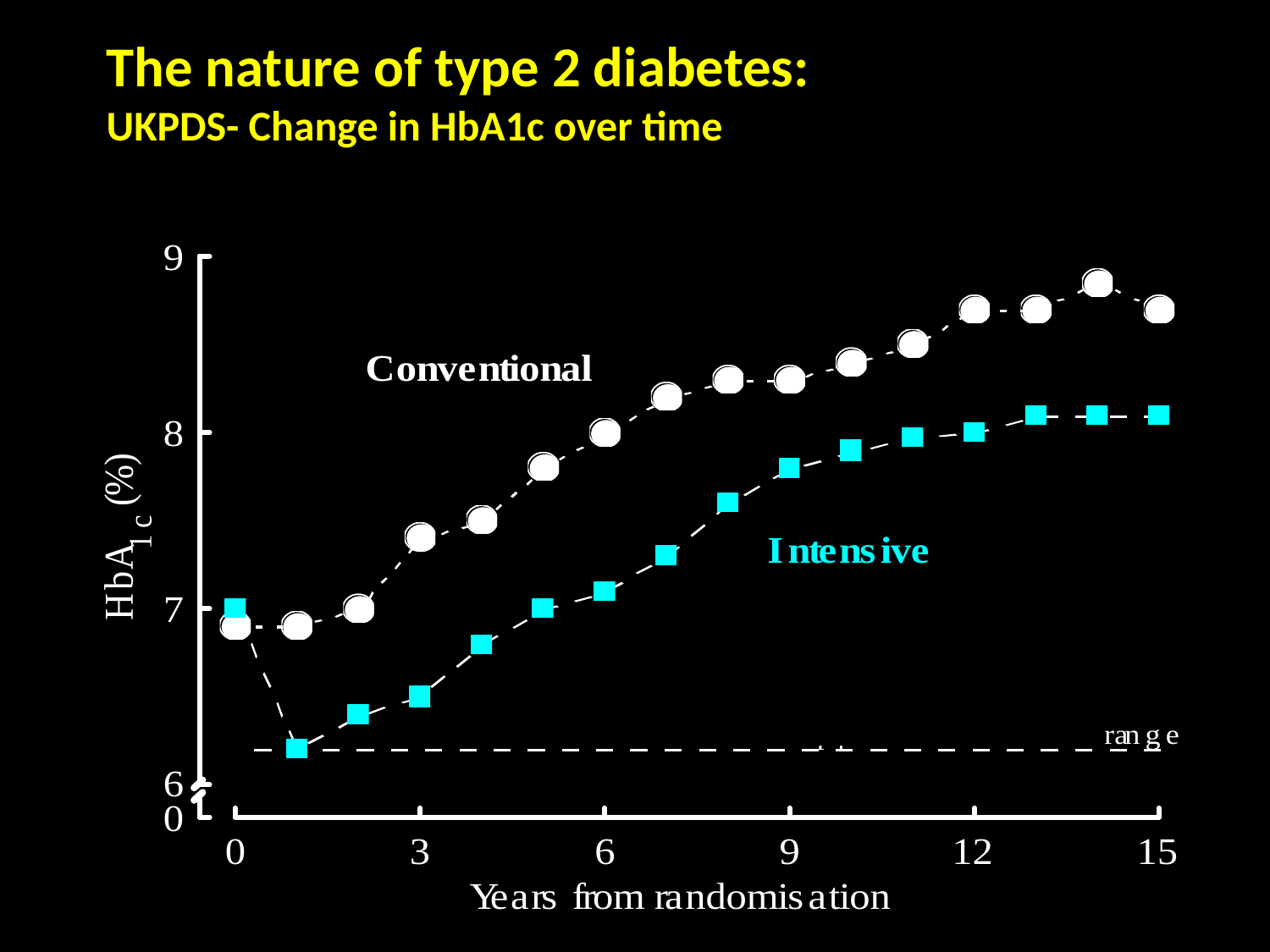

The nature of type 2 diabetes:
UKPDS- Change in HbA1c over time

## Slide 9
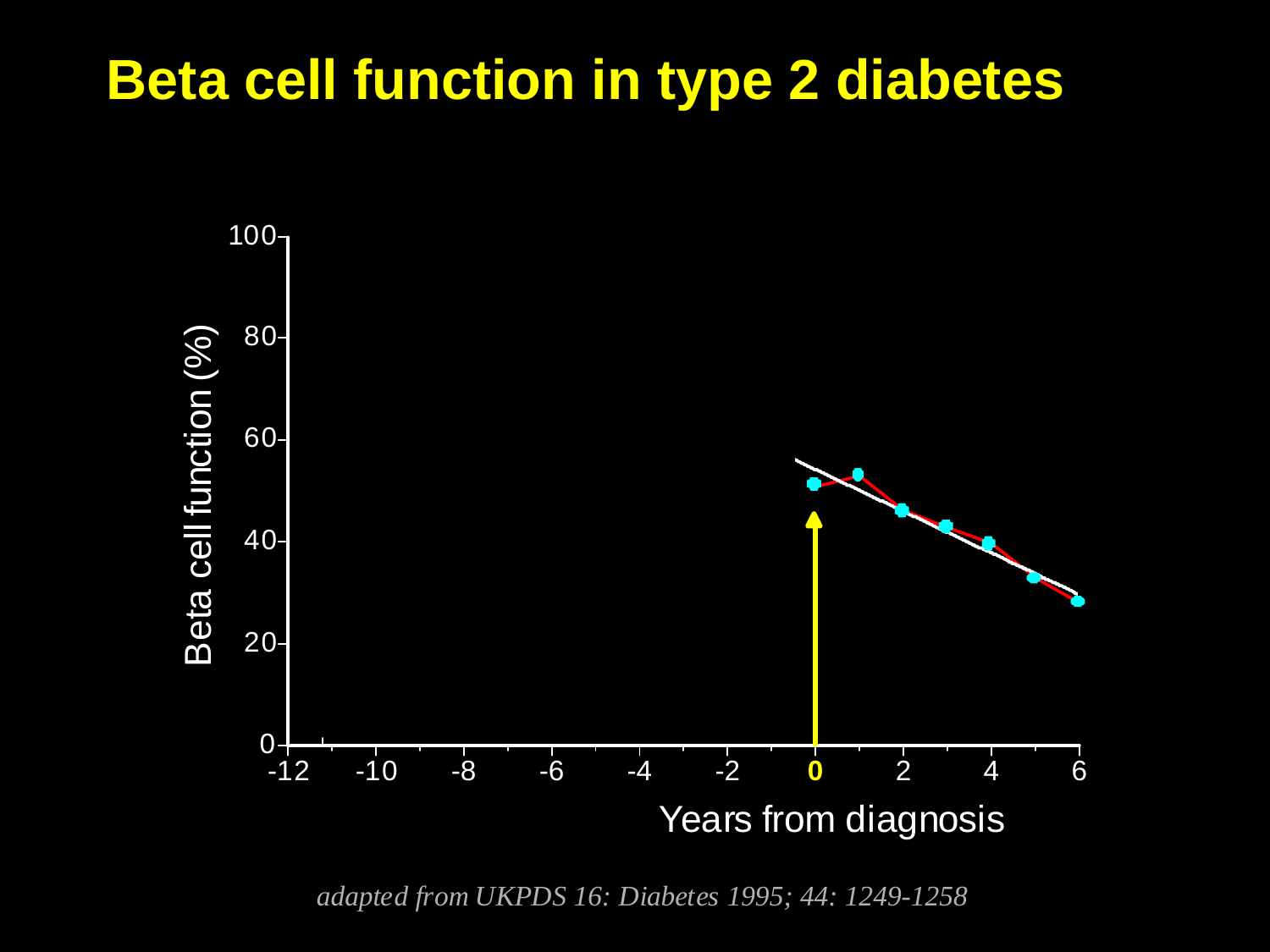

Beta cell function in type 2 diabetes

## Slide 10
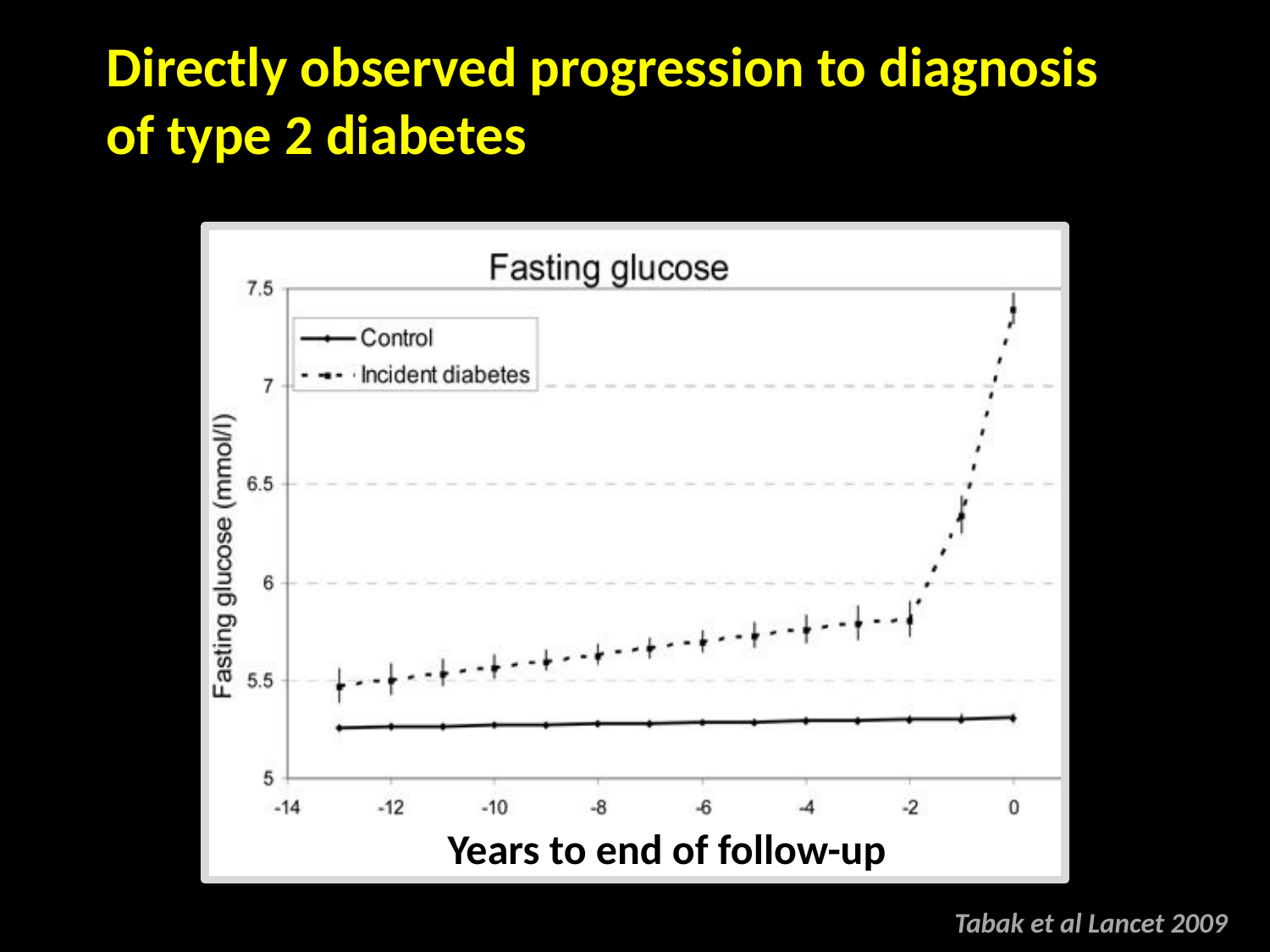

Directly observed progression to diagnosis of type 2 diabetes
Years to end of follow-up
Tabak et al Lancet 2009

## Slide 11
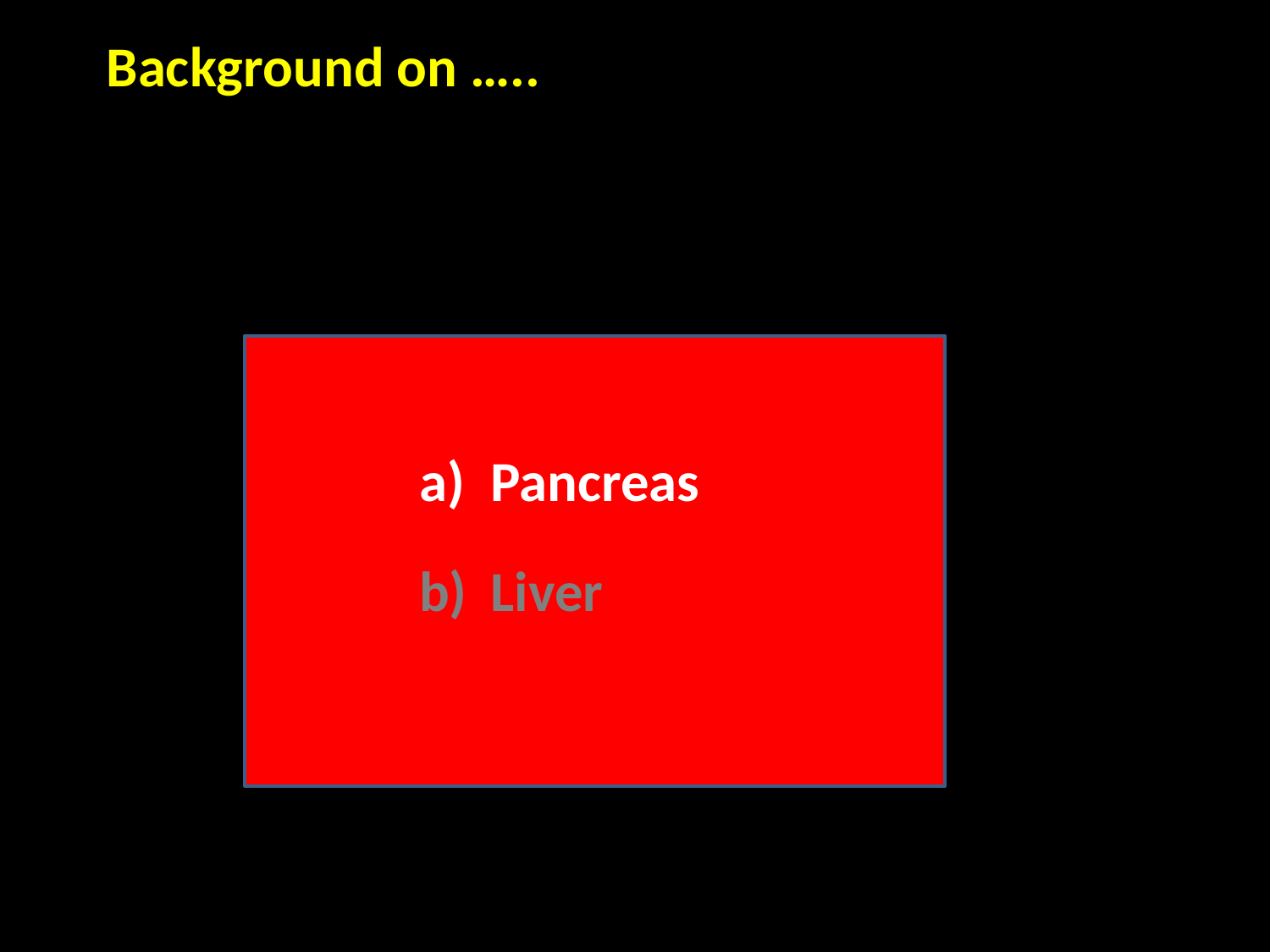

Background on …..
Pancreas
Liver

## Slide 12
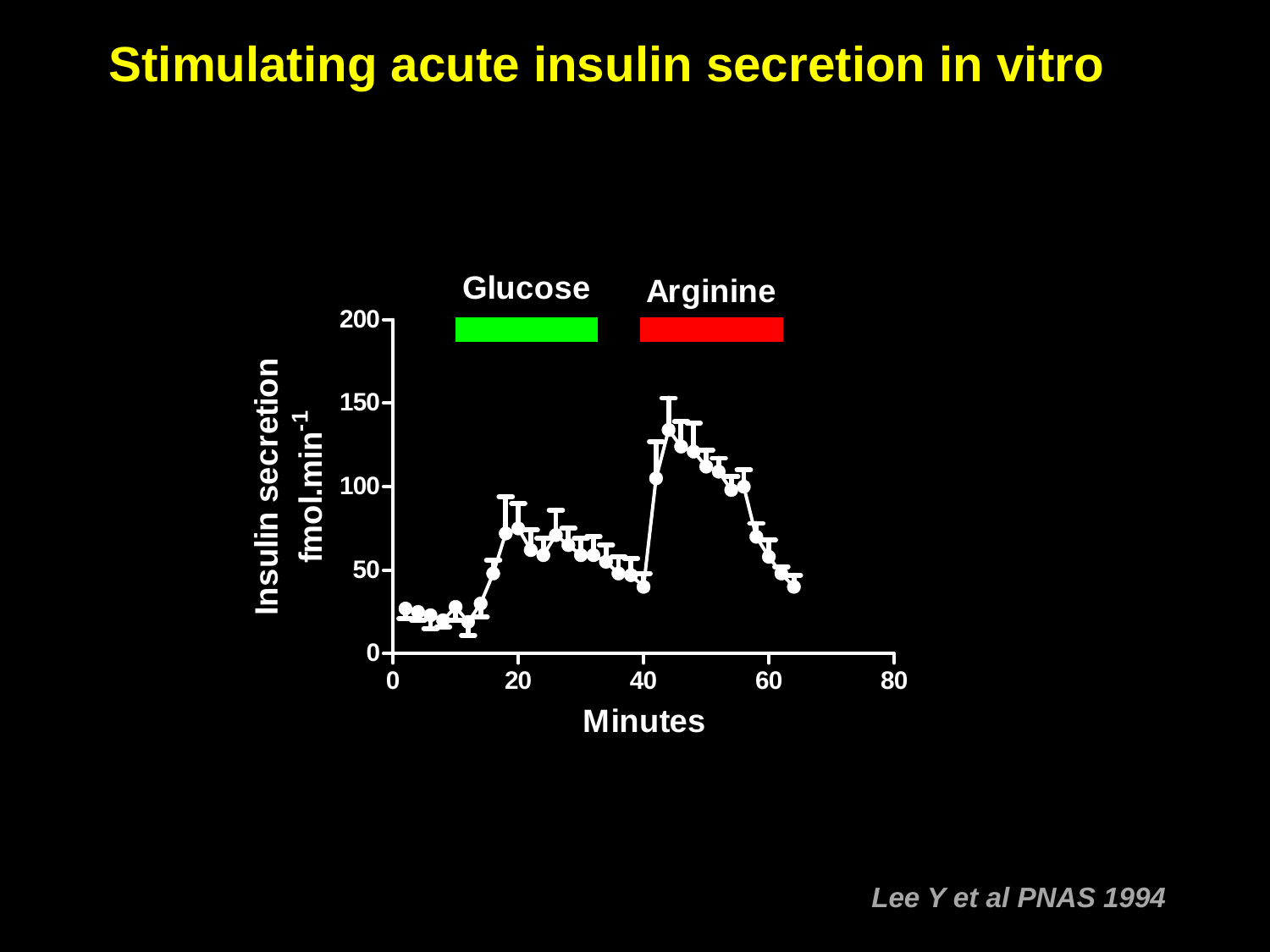

Stimulating acute insulin secretion in vitro
Lee Y et al PNAS 1994

## Slide 13
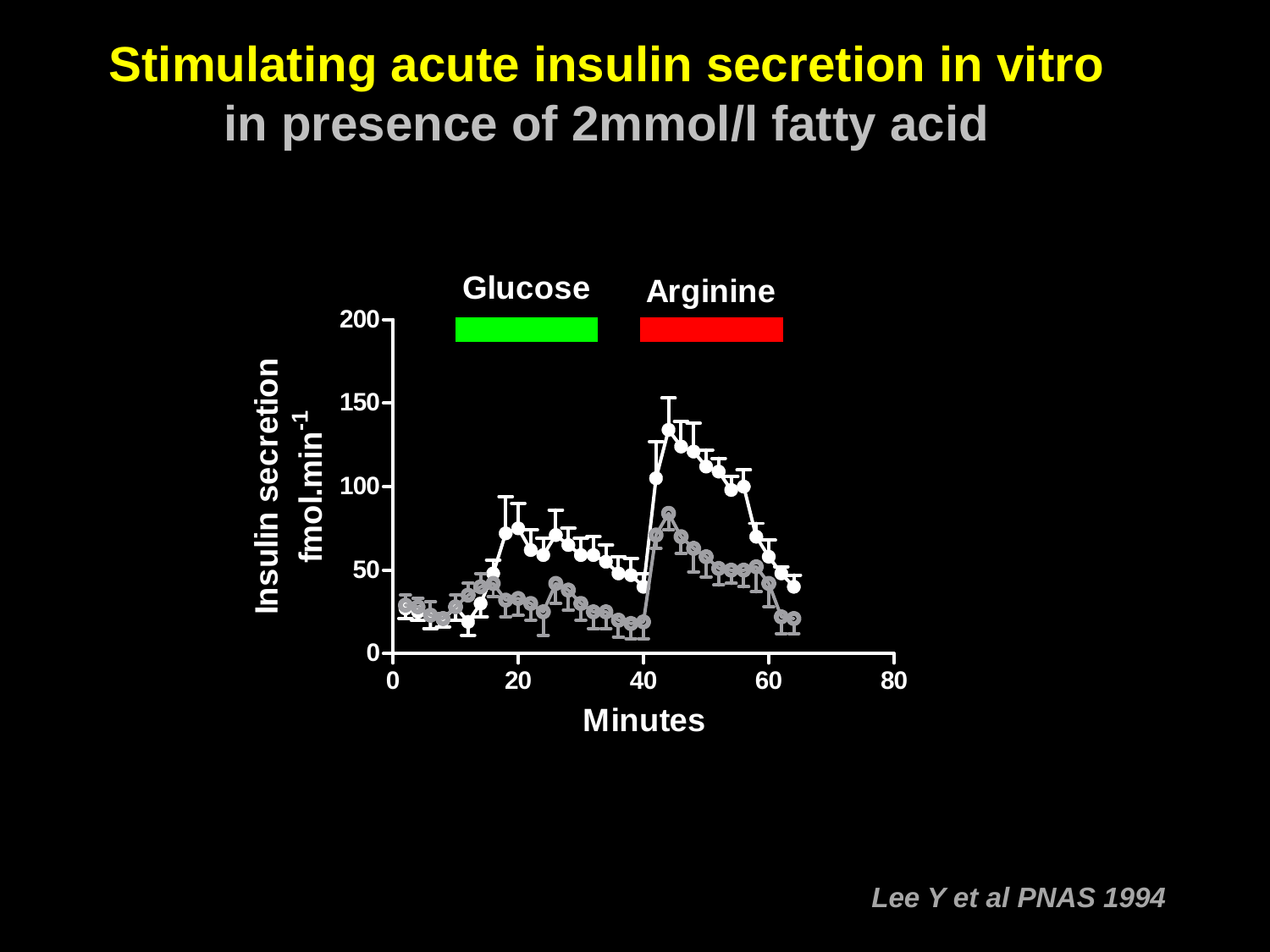

Stimulating acute insulin secretion in vitro in presence of 2mmol/l fatty acid
Lee Y et al PNAS 1994

## Slide 14
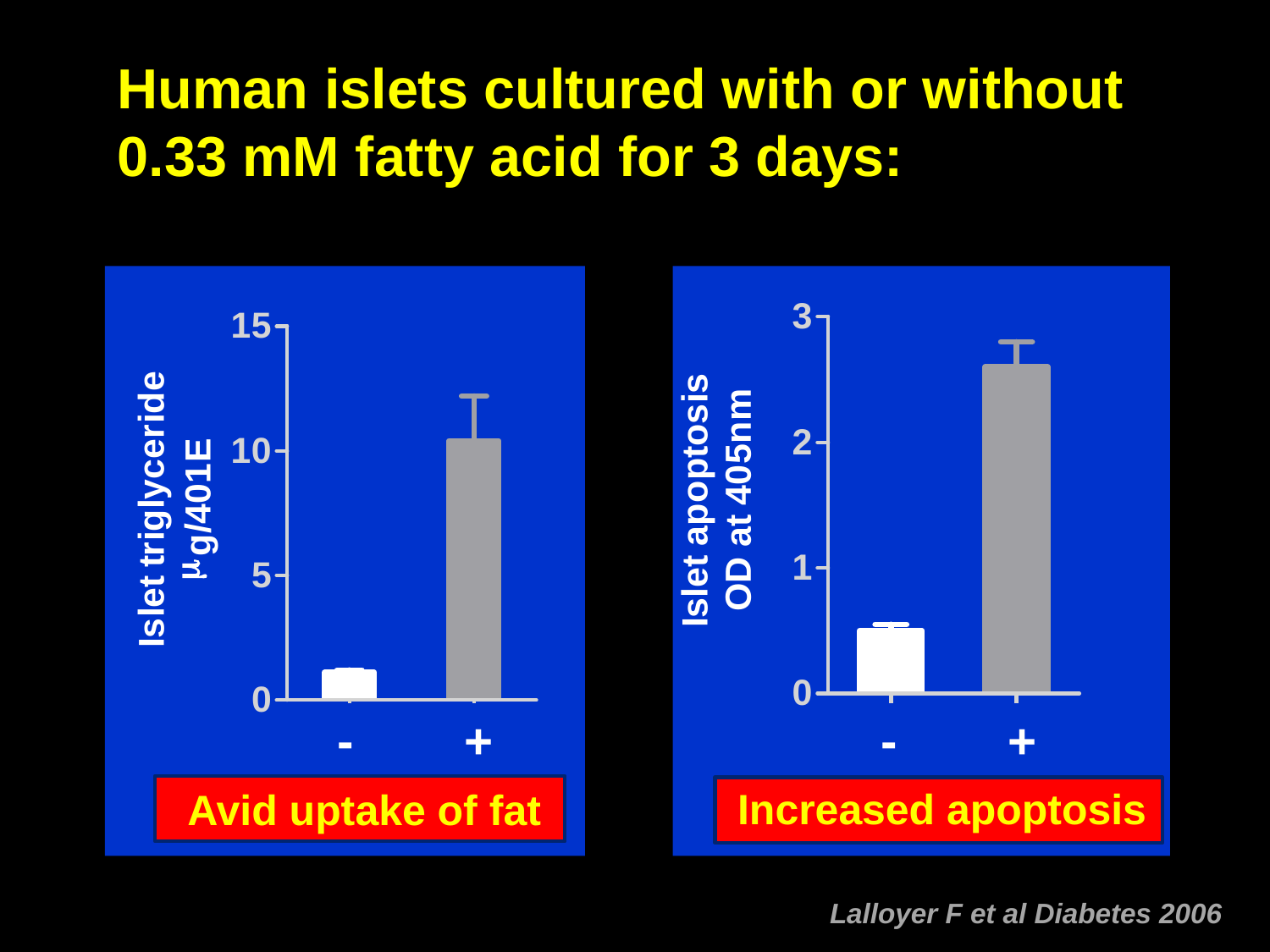

Human islets cultured with or without 0.33 mM fatty acid for 3 days:
- 	+
Increased apoptosis
- 	+
Avid uptake of fat
Lalloyer F et al Diabetes 2006

## Slide 15
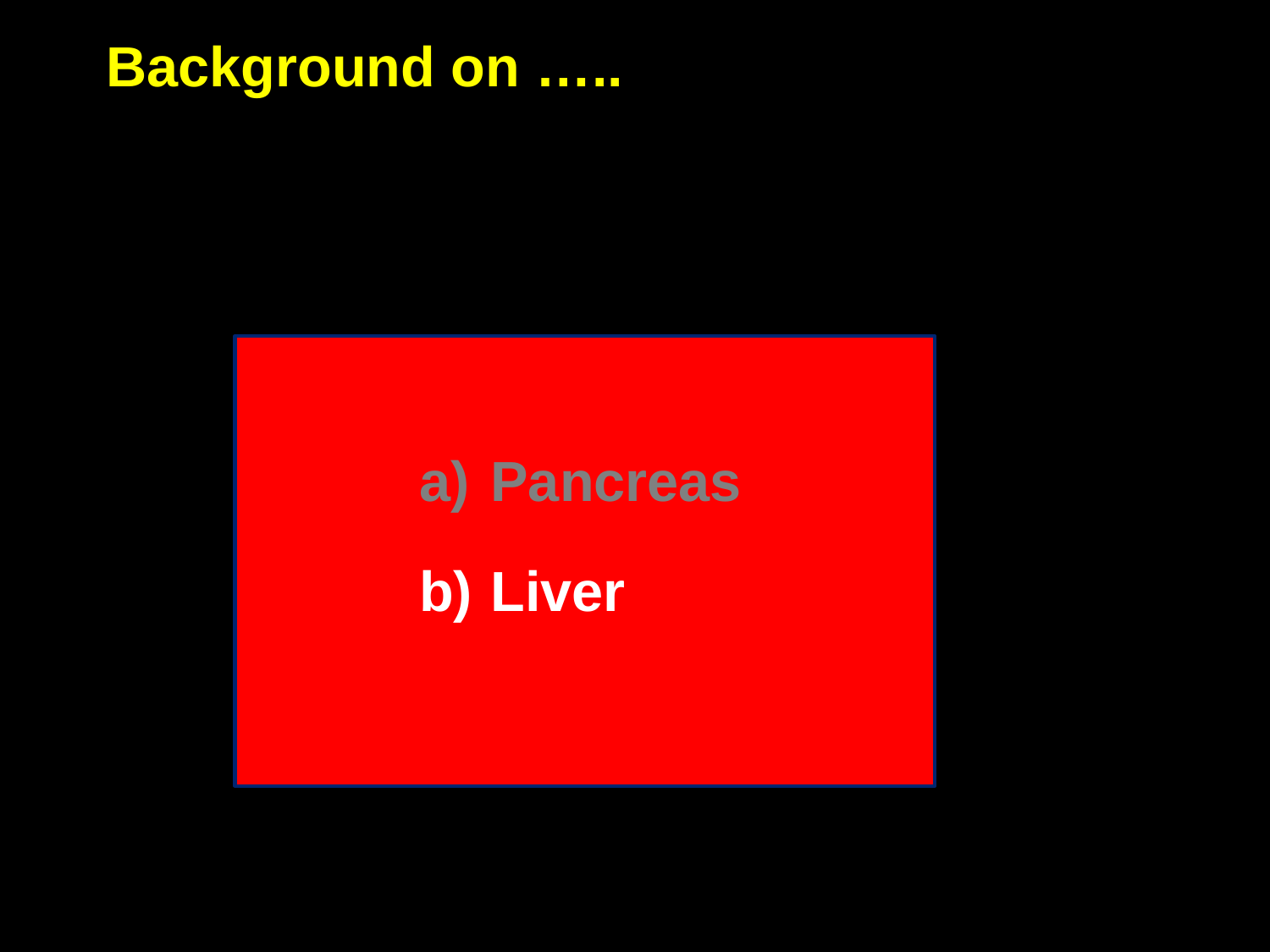

Background on …..
Pancreas
Liver

## Slide 16
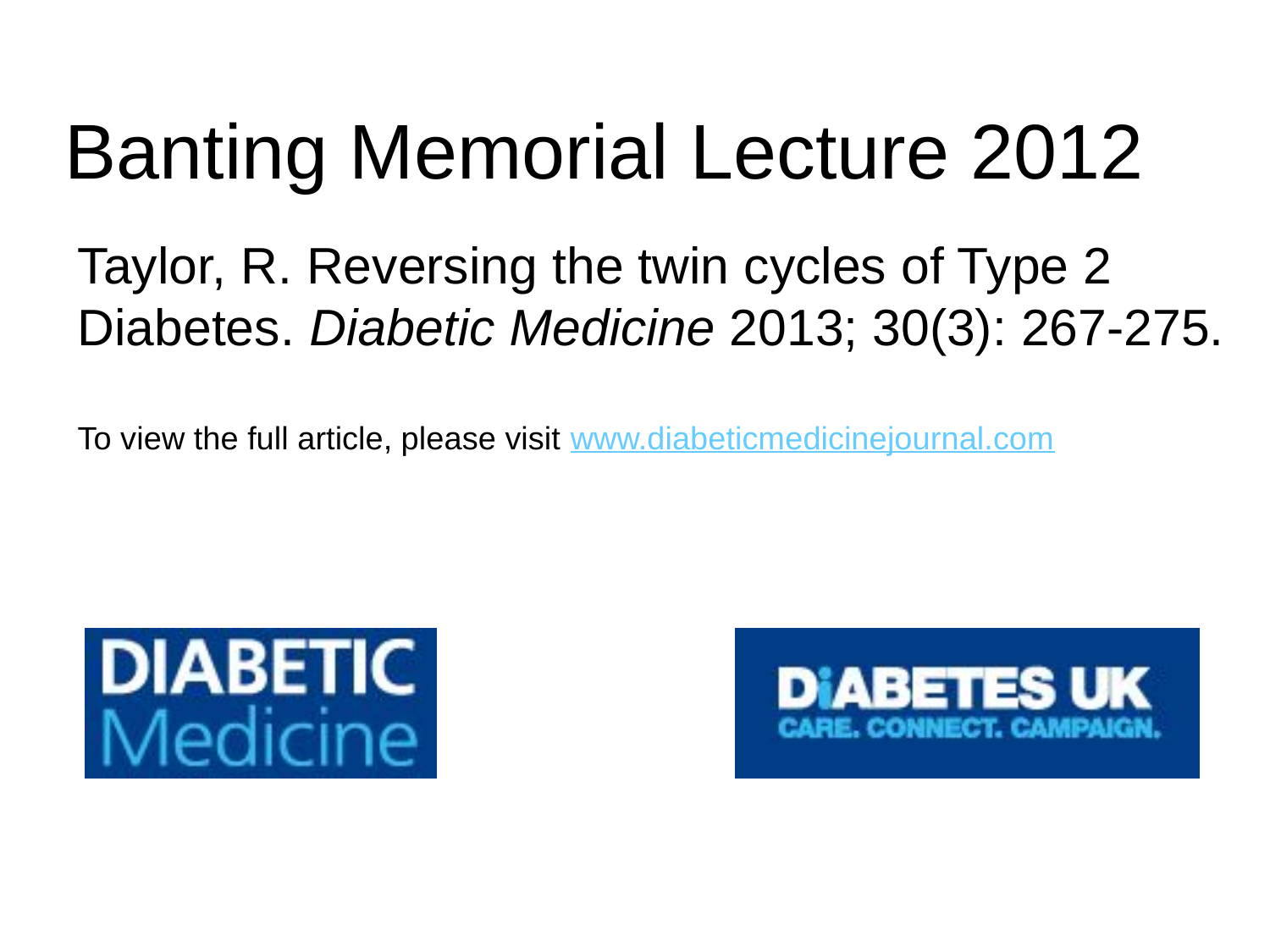

# Banting Memorial Lecture 2012
Taylor, R. Reversing the twin cycles of Type 2 Diabetes. Diabetic Medicine 2013; 30(3): 267-275.
To view the full article, please visit www.diabeticmedicinejournal.com

## Slide 17
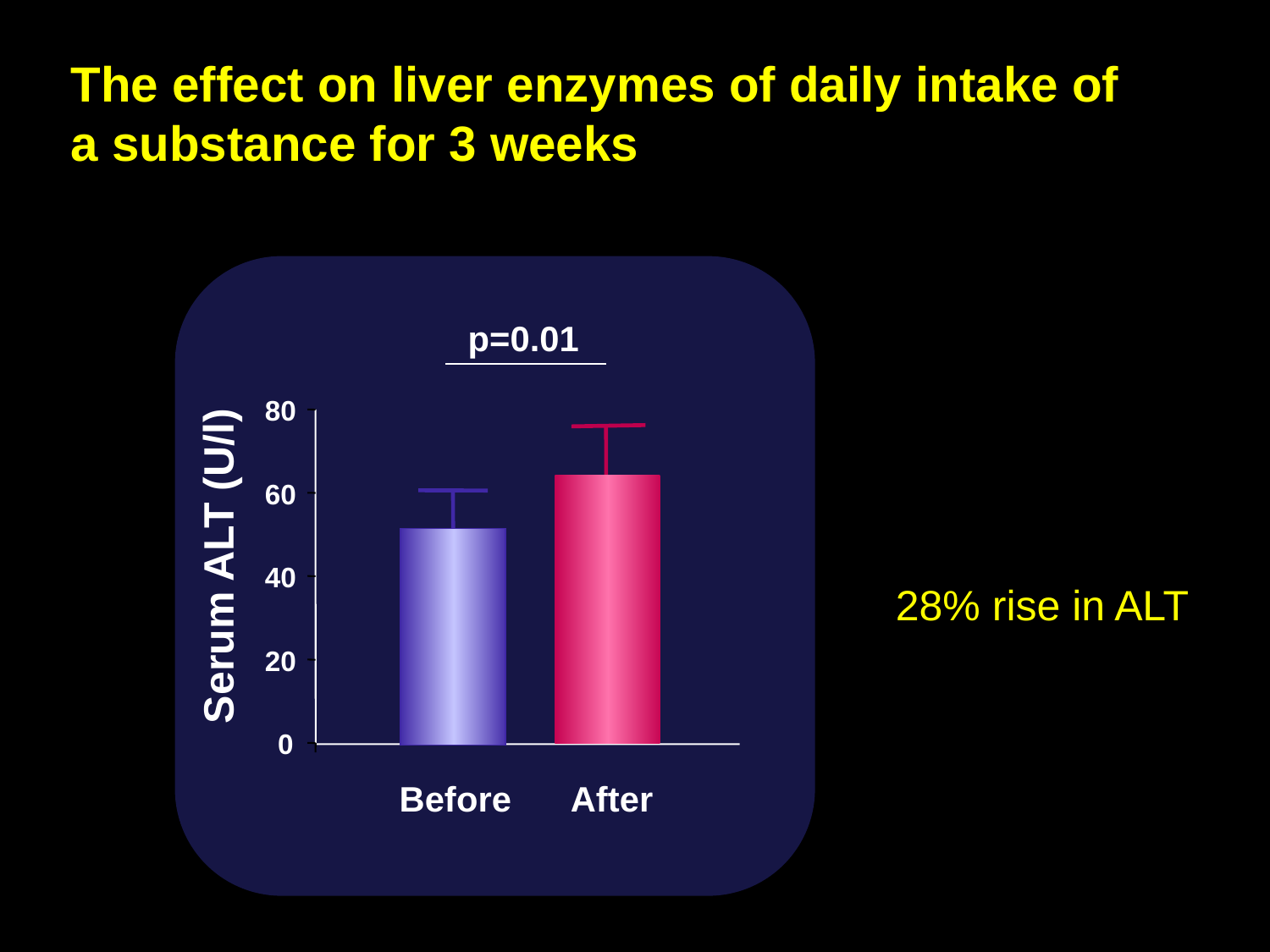

The effect on liver enzymes of daily intake of a substance for 3 weeks
p=0.01
80
60
Serum ALT (U/l)
40
28% rise in ALT
20
0
 Before
 After

## Slide 18
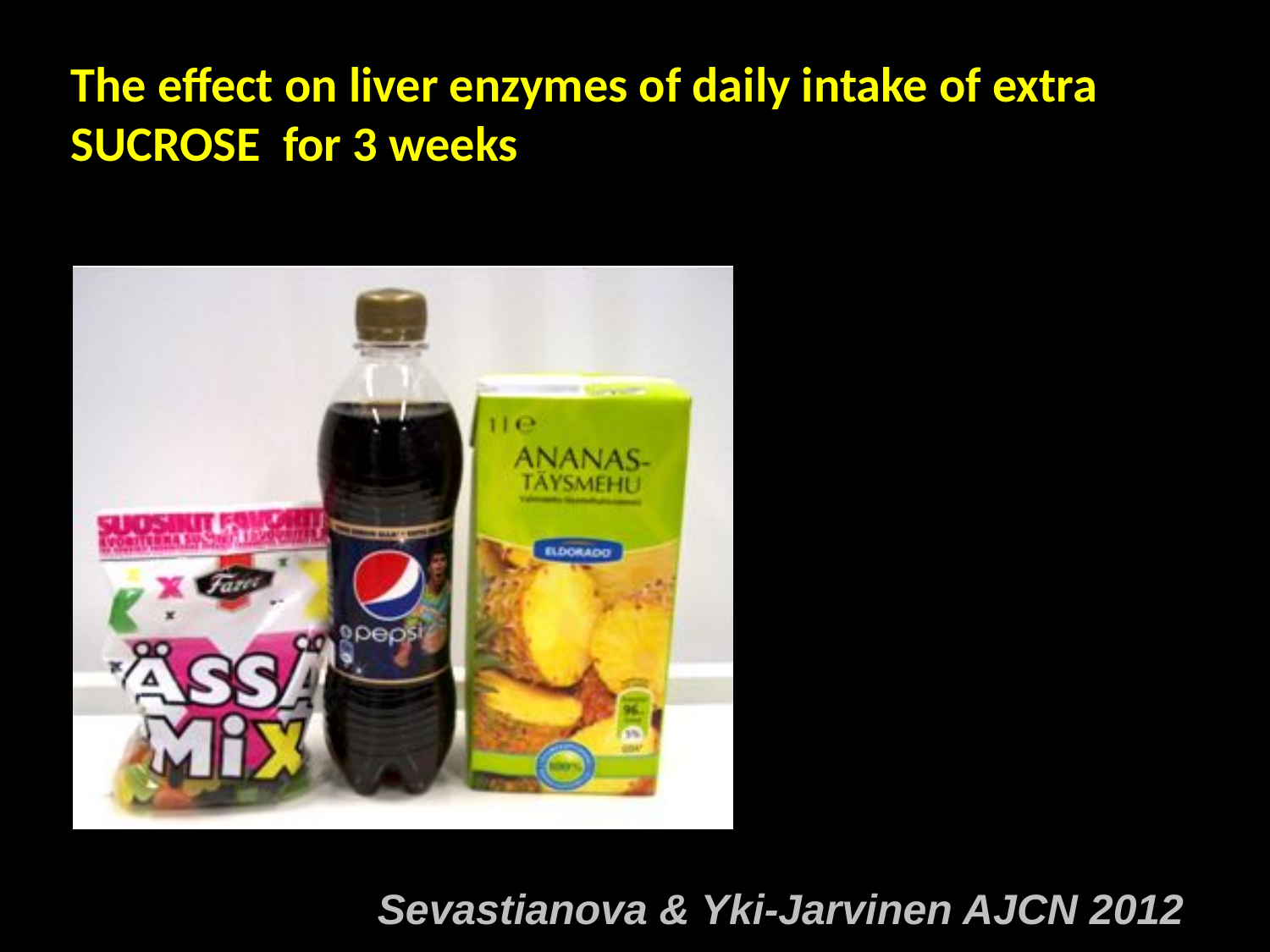

The effect on liver enzymes of daily intake of extra SUCROSE for 3 weeks
Overfeeding: 1000 extra simple sugar calories/day
Sevastianova & Yki-Jarvinen AJCN 2012

## Slide 19
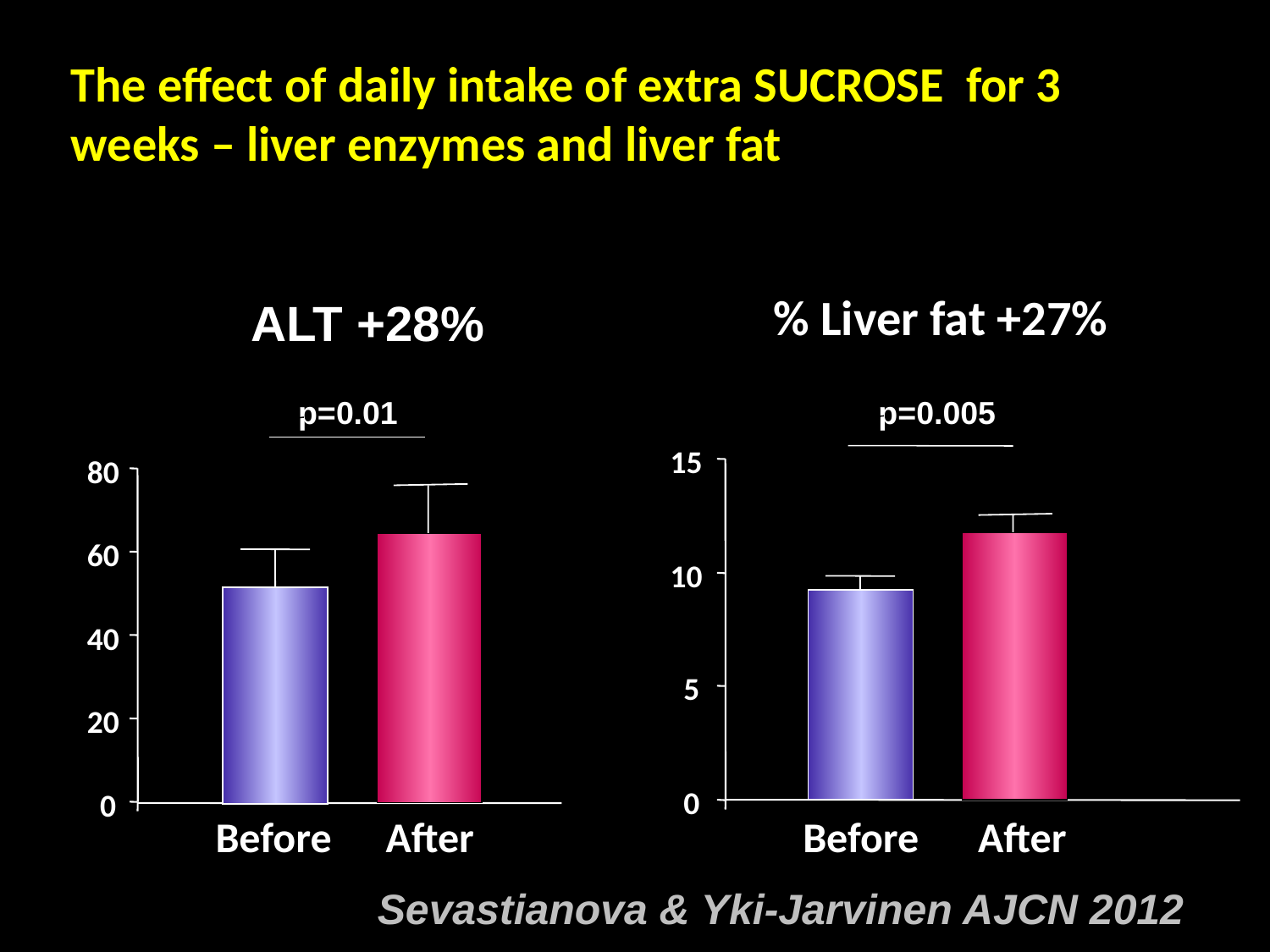

The effect of daily intake of extra SUCROSE for 3 weeks – liver enzymes and liver fat
ALT +28%
% Liver fat +27%
p=0.01
p=0.005
15
80
60
10
40
5
20
0
0
 Before
 After
 Before
 After
Sevastianova & Yki-Jarvinen AJCN 2012

## Slide 20
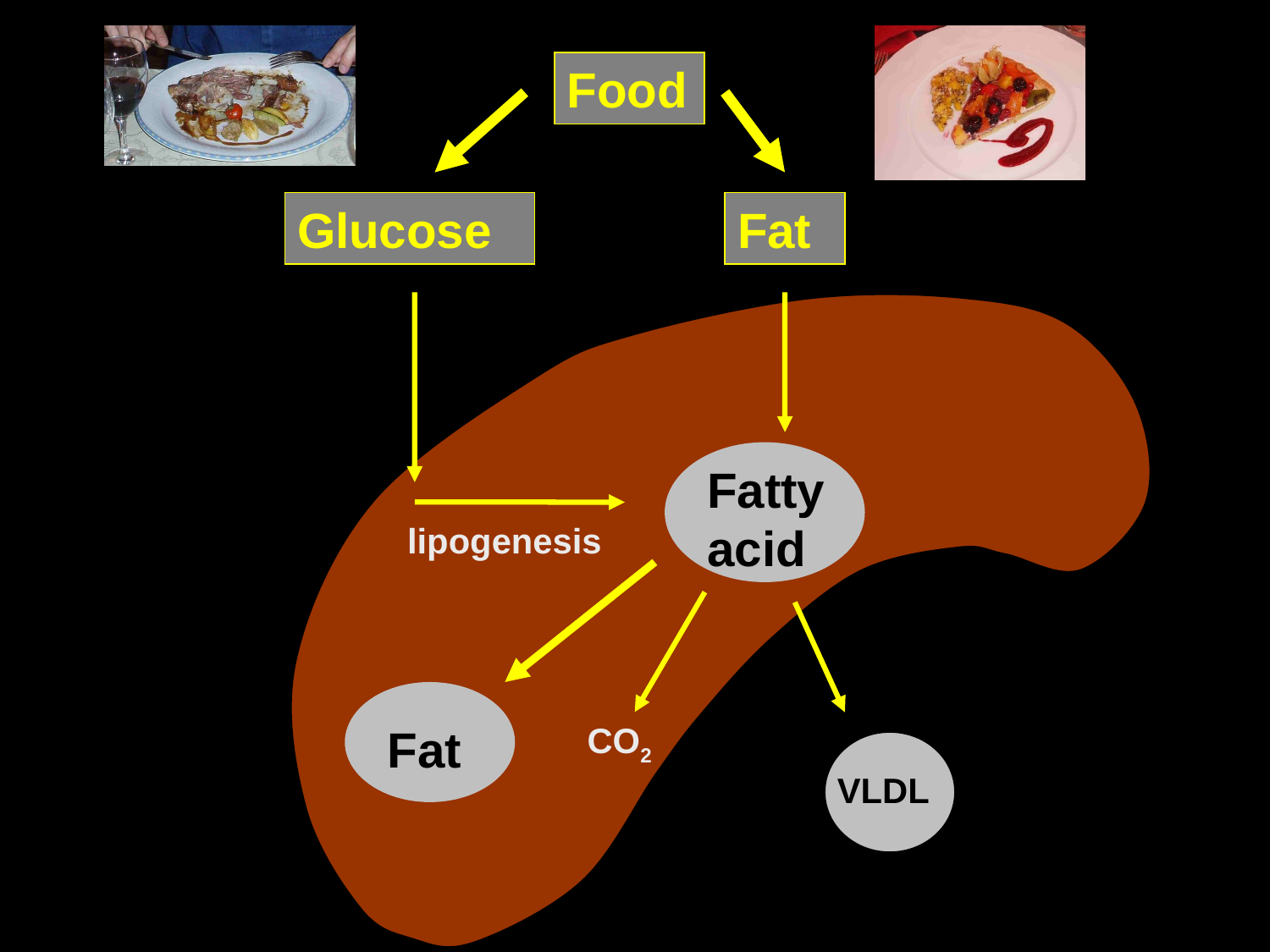

Food
Glucose
Fat
Fatty acid
lipogenesis
Fat
CO2
VLDL

## Slide 21
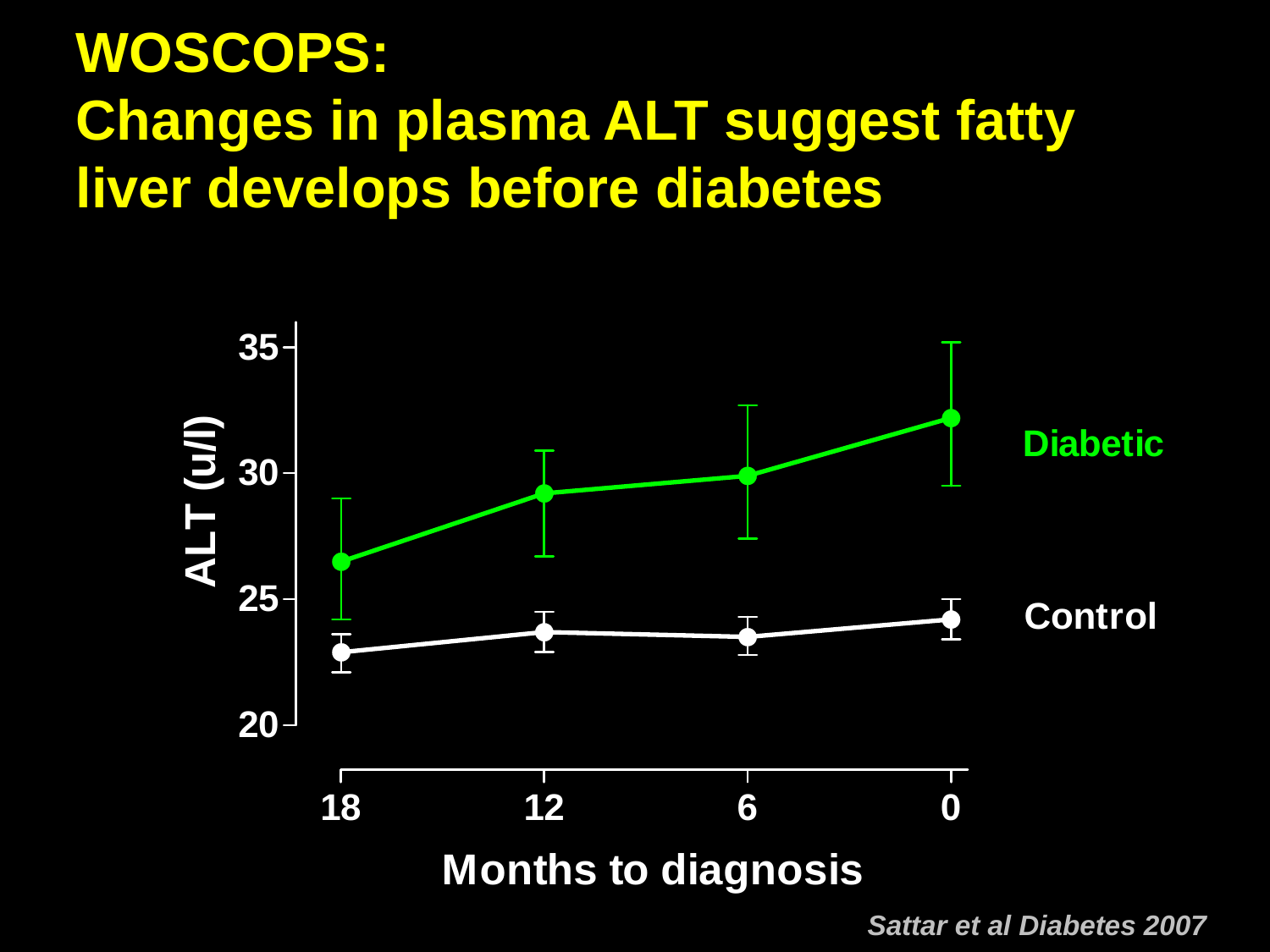

WOSCOPS:
Changes in plasma ALT suggest fatty liver develops before diabetes
Sattar et al Diabetes 2007

## Slide 22
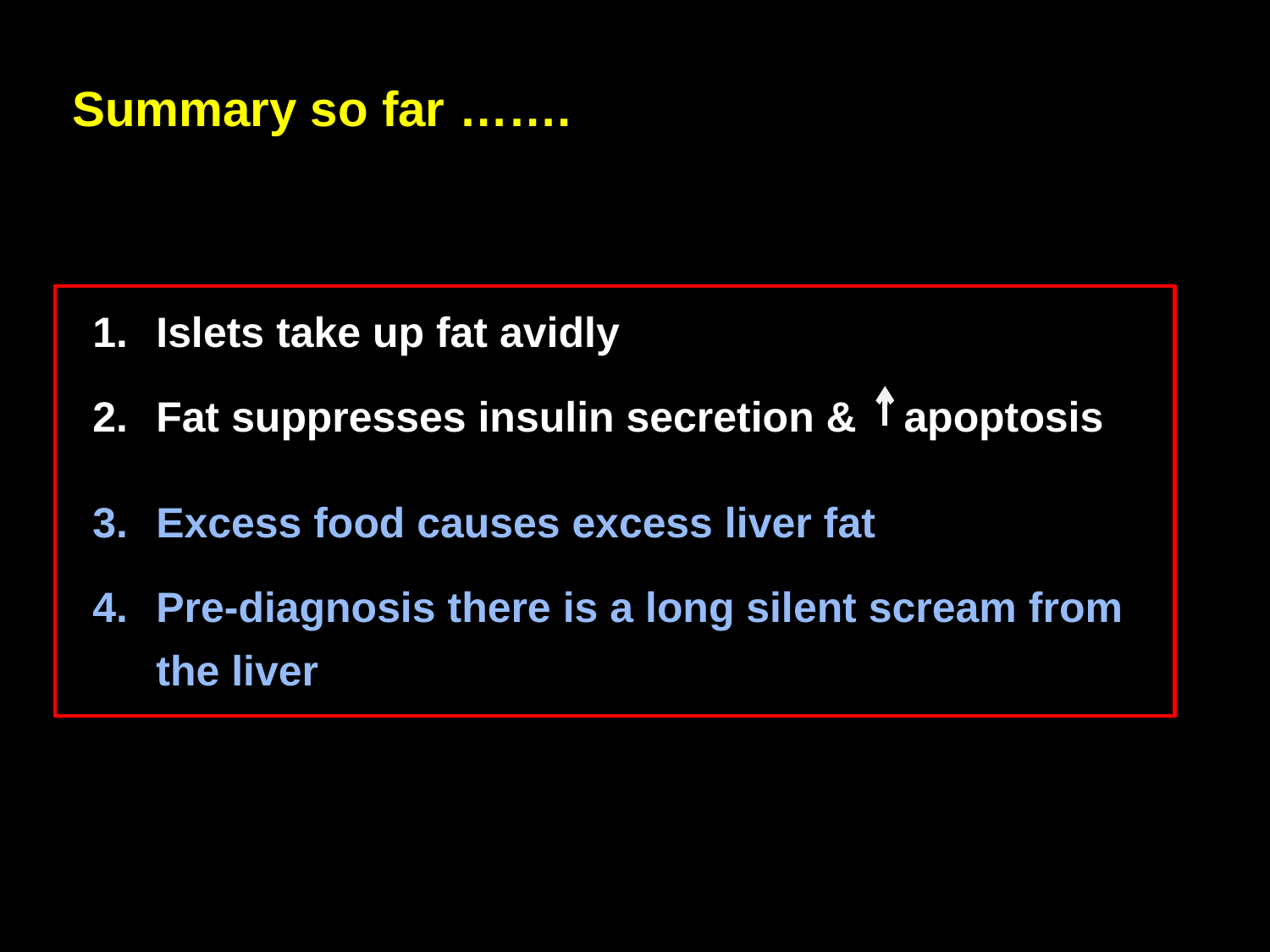

# Summary so far …….
Islets take up fat avidly
Fat suppresses insulin secretion & apoptosis
Excess food causes excess liver fat
Pre-diagnosis there is a long silent scream from the liver

## Slide 23
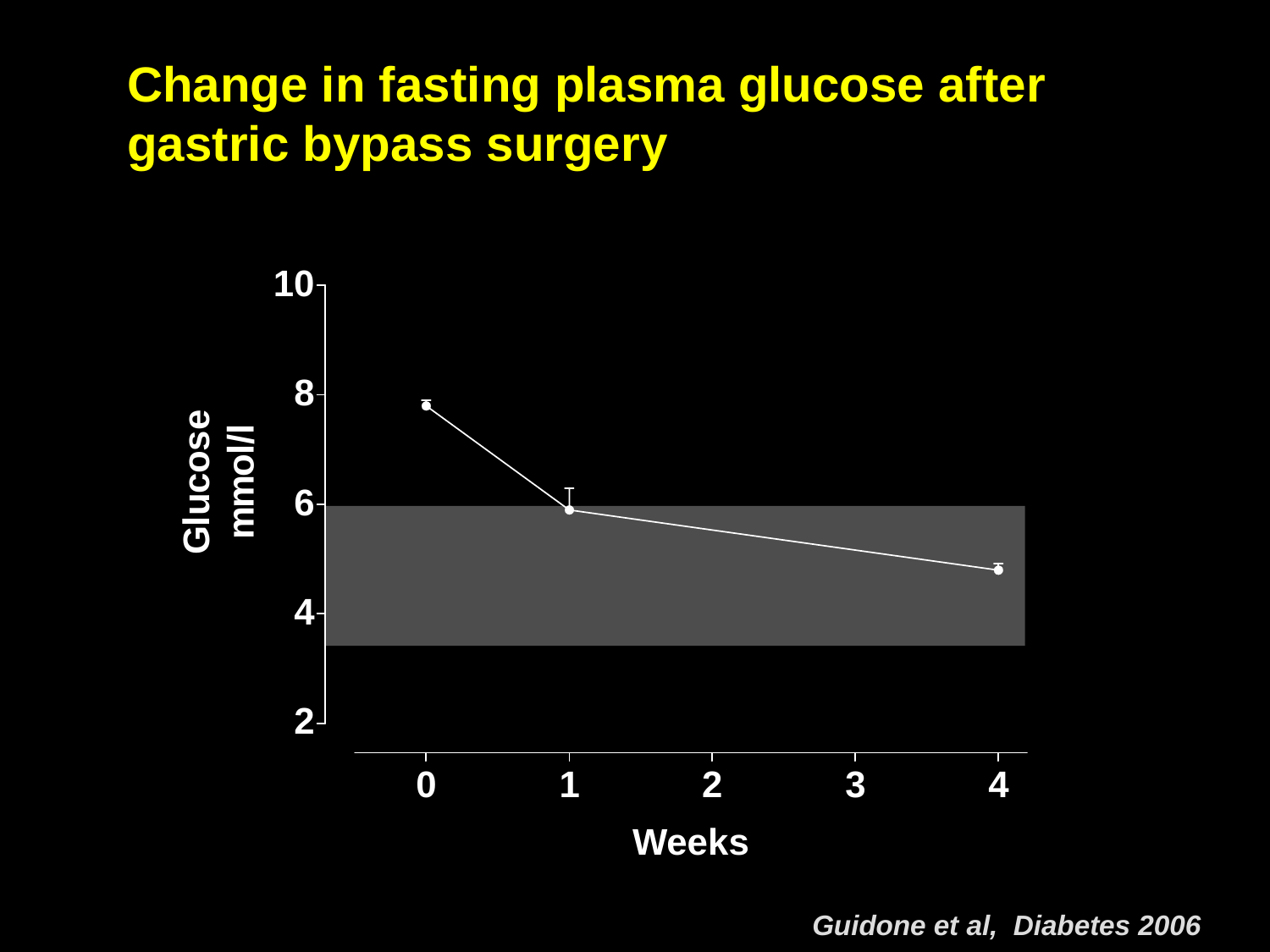

Change in fasting plasma glucose after gastric bypass surgery
Guidone et al, Diabetes 2006

## Slide 24
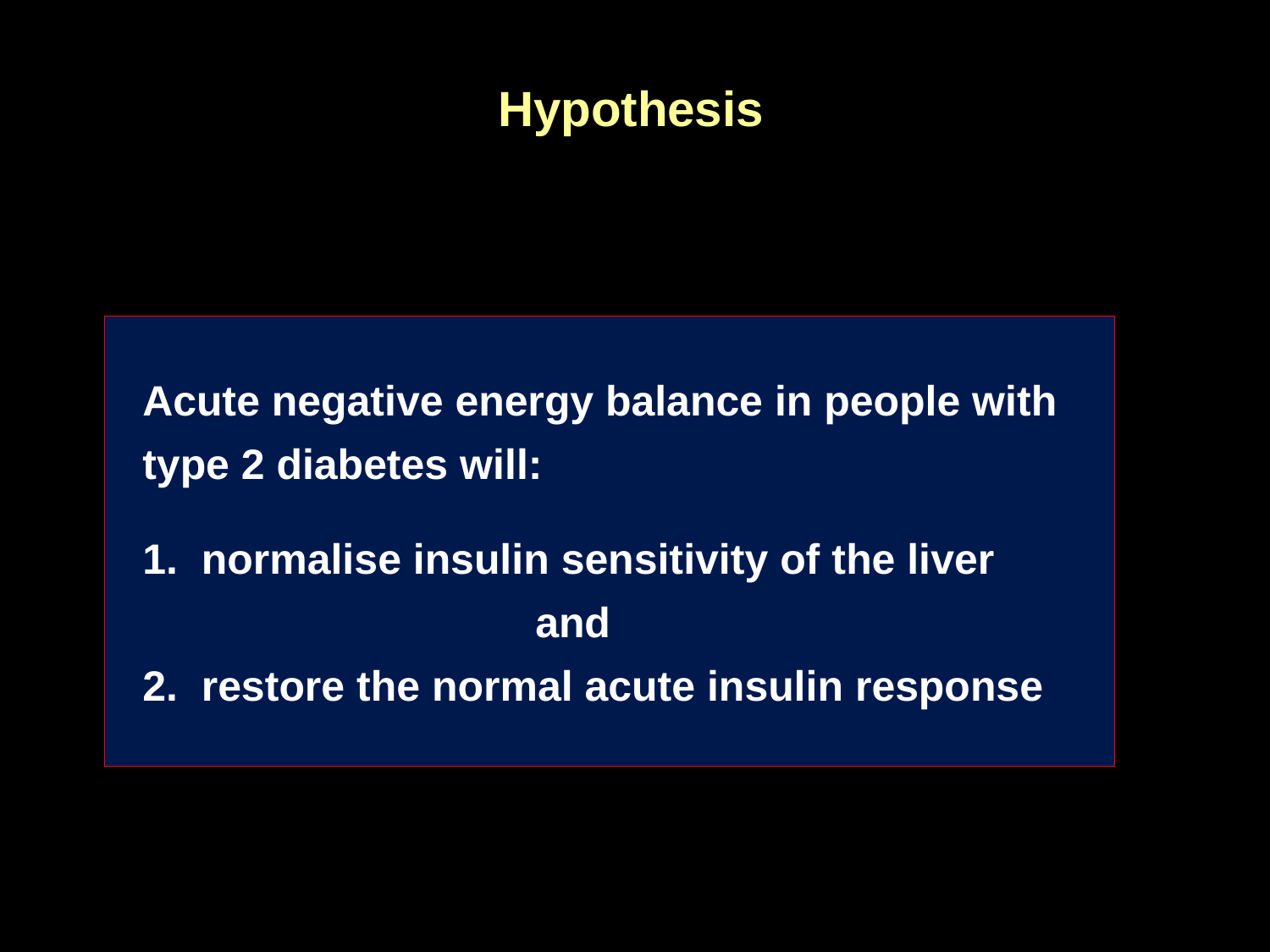

# Hypothesis
Acute negative energy balance in people with type 2 diabetes will:
1. normalise insulin sensitivity of the liver
			 and
2. restore the normal acute insulin response

## Slide 25
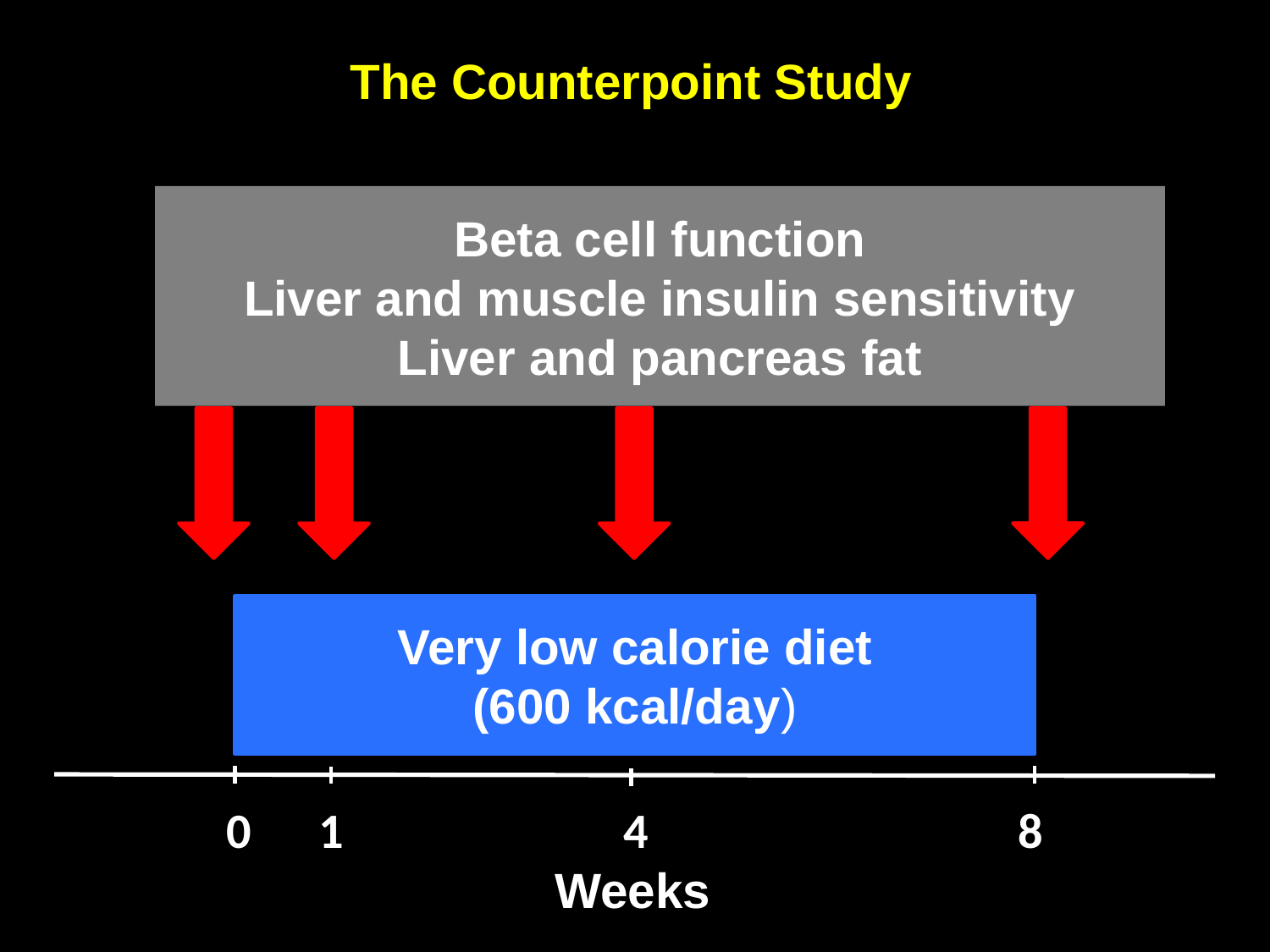

# The Counterpoint Study
Beta cell function
Liver and muscle insulin sensitivity
Liver and pancreas fat
Very low calorie diet
(600 kcal/day)
 0 1 4 8
Weeks

## Slide 26
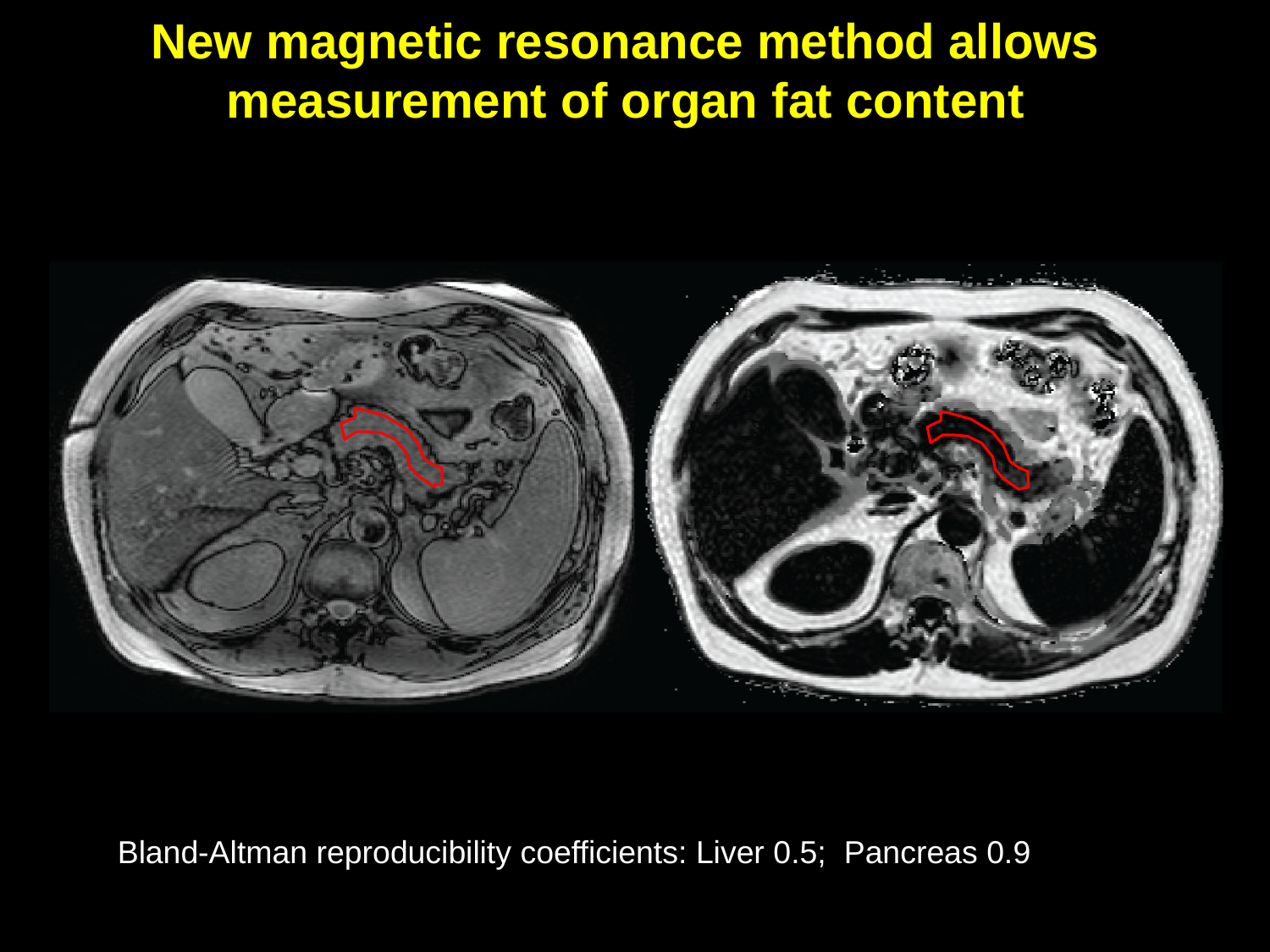

New magnetic resonance method allows measurement of organ fat content
Bland-Altman reproducibility coefficients: Liver 0.5; Pancreas 0.9

## Slide 27
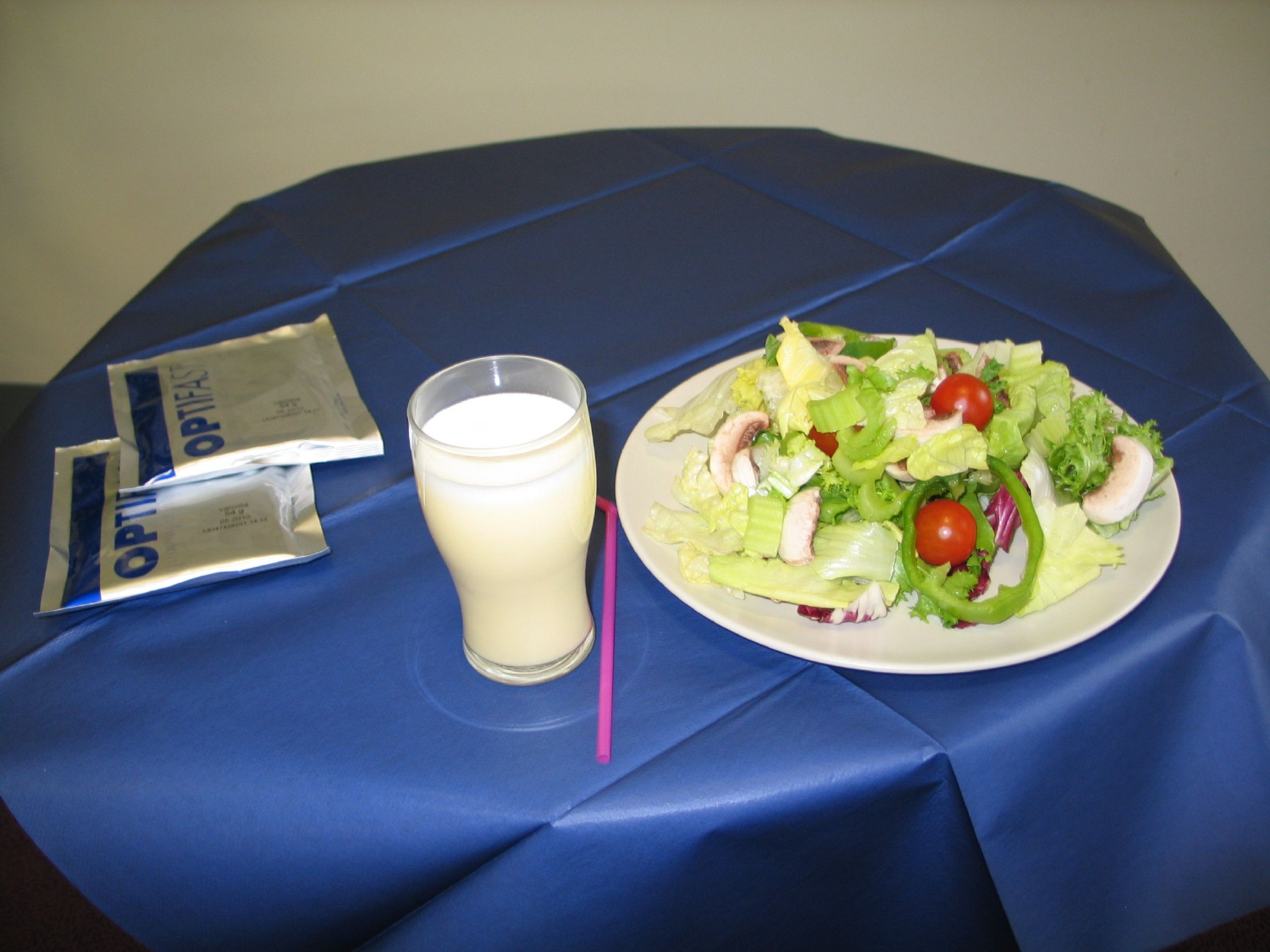

Picture of Optifast

## Slide 28
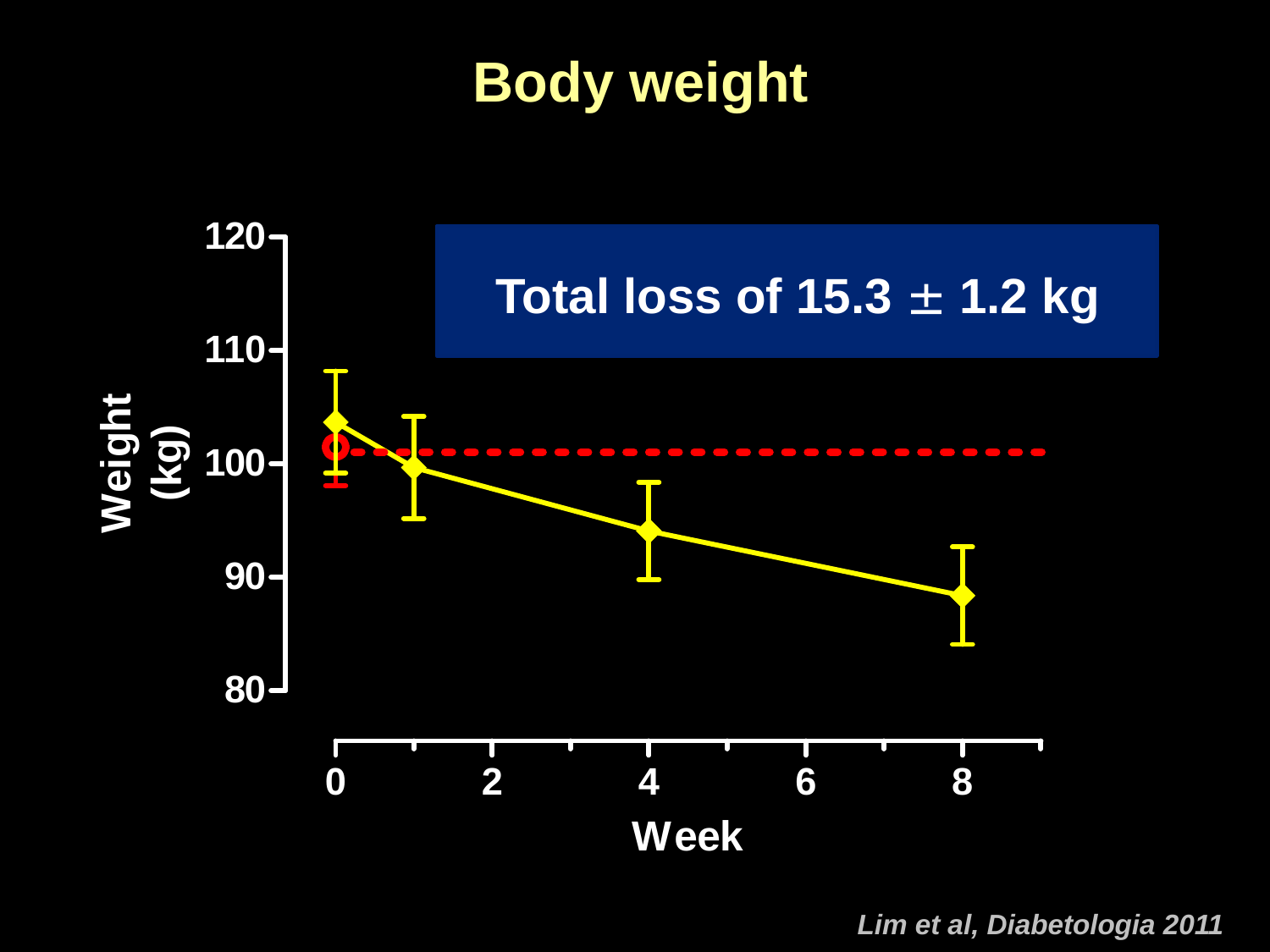

# Body weight
Total loss of 15.3 ± 1.2 kg
First week:  3.9 ± 0.2 kg
Lim et al, Diabetologia 2011

## Slide 29
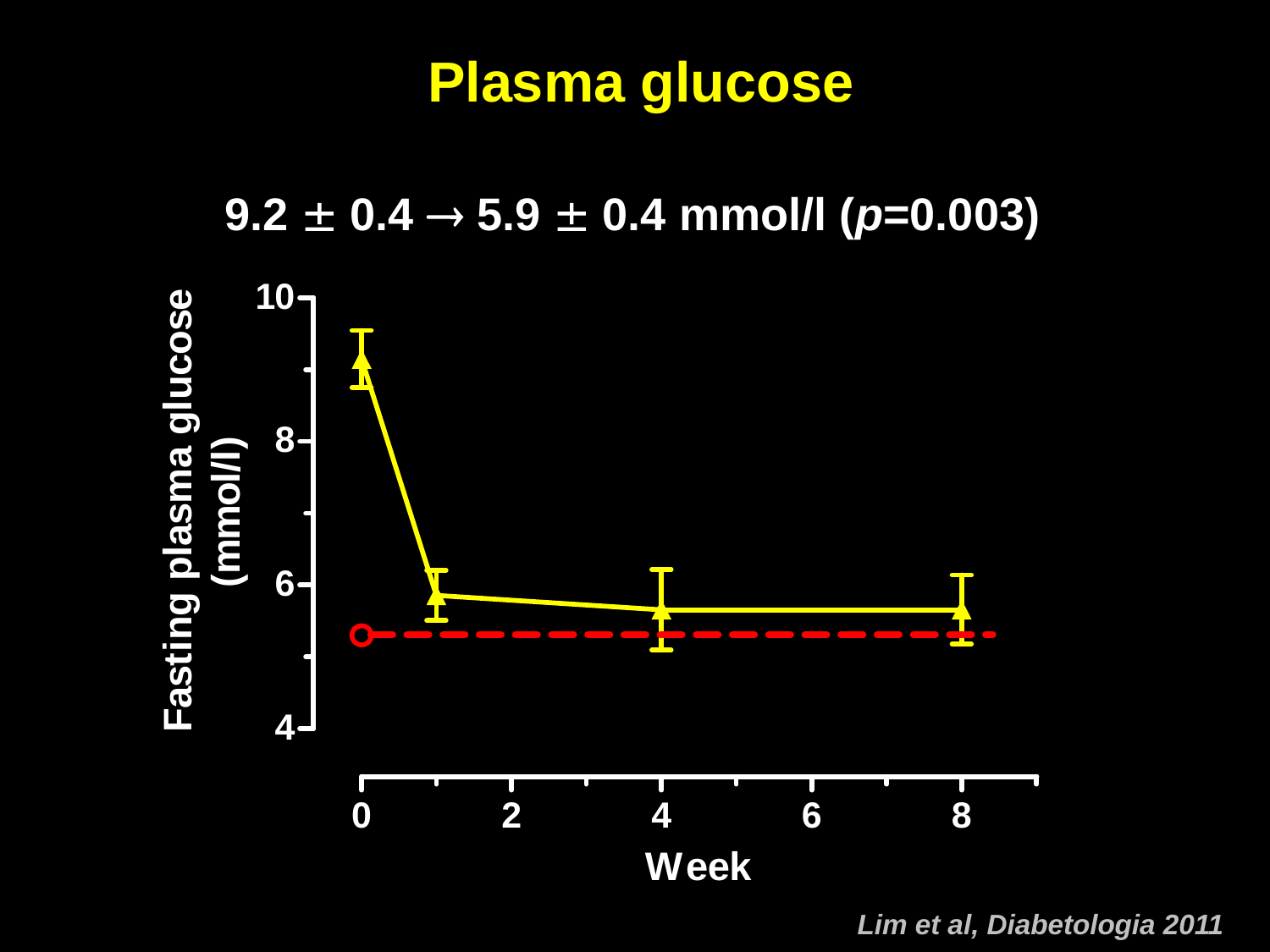

# Plasma glucose
9.2 ± 0.4  5.9 ± 0.4 mmol/l (p=0.003)
Lim et al, Diabetologia 2011

## Slide 30
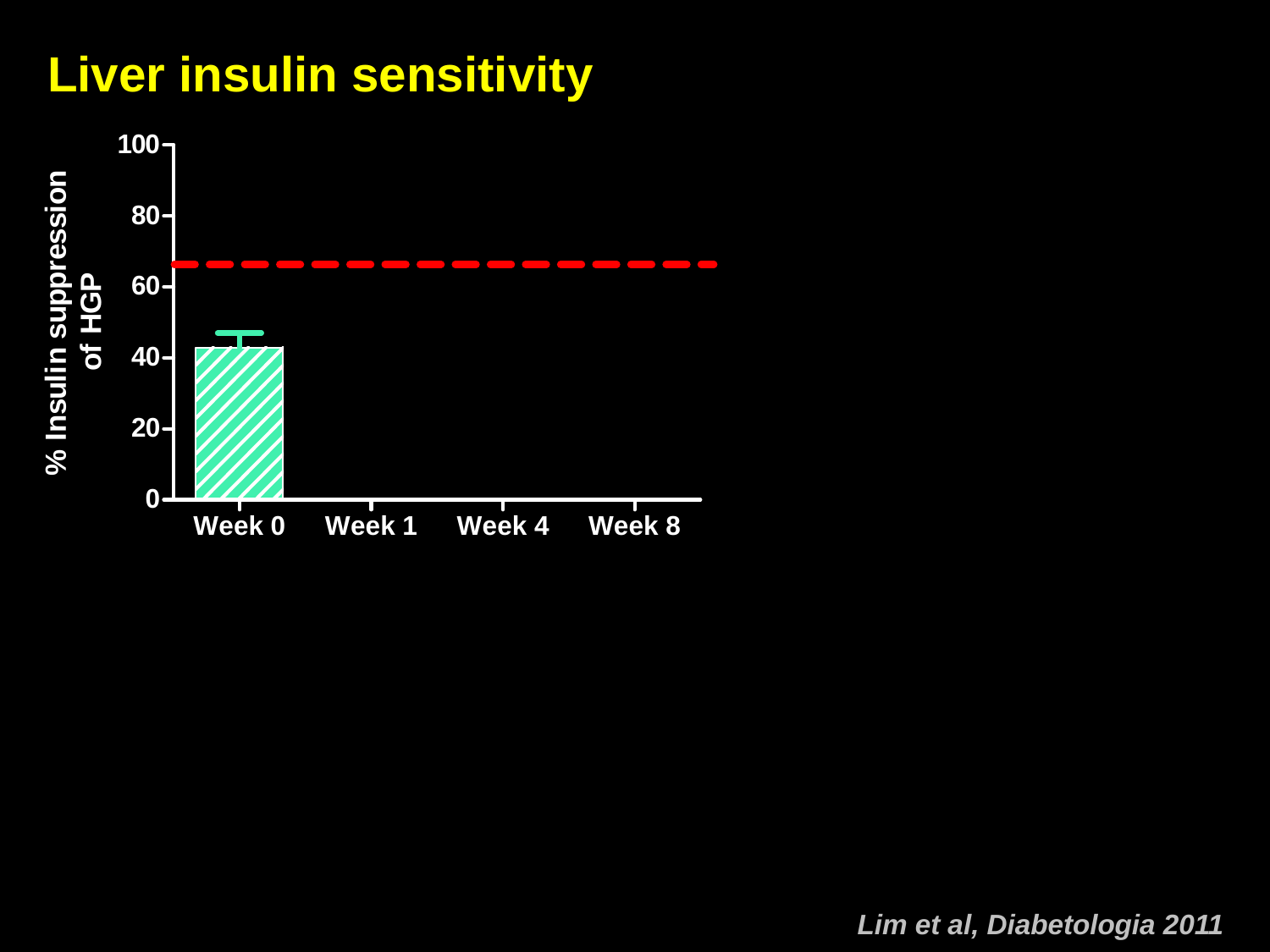

Liver insulin sensitivity
Lim et al, Diabetologia 2011

## Slide 31
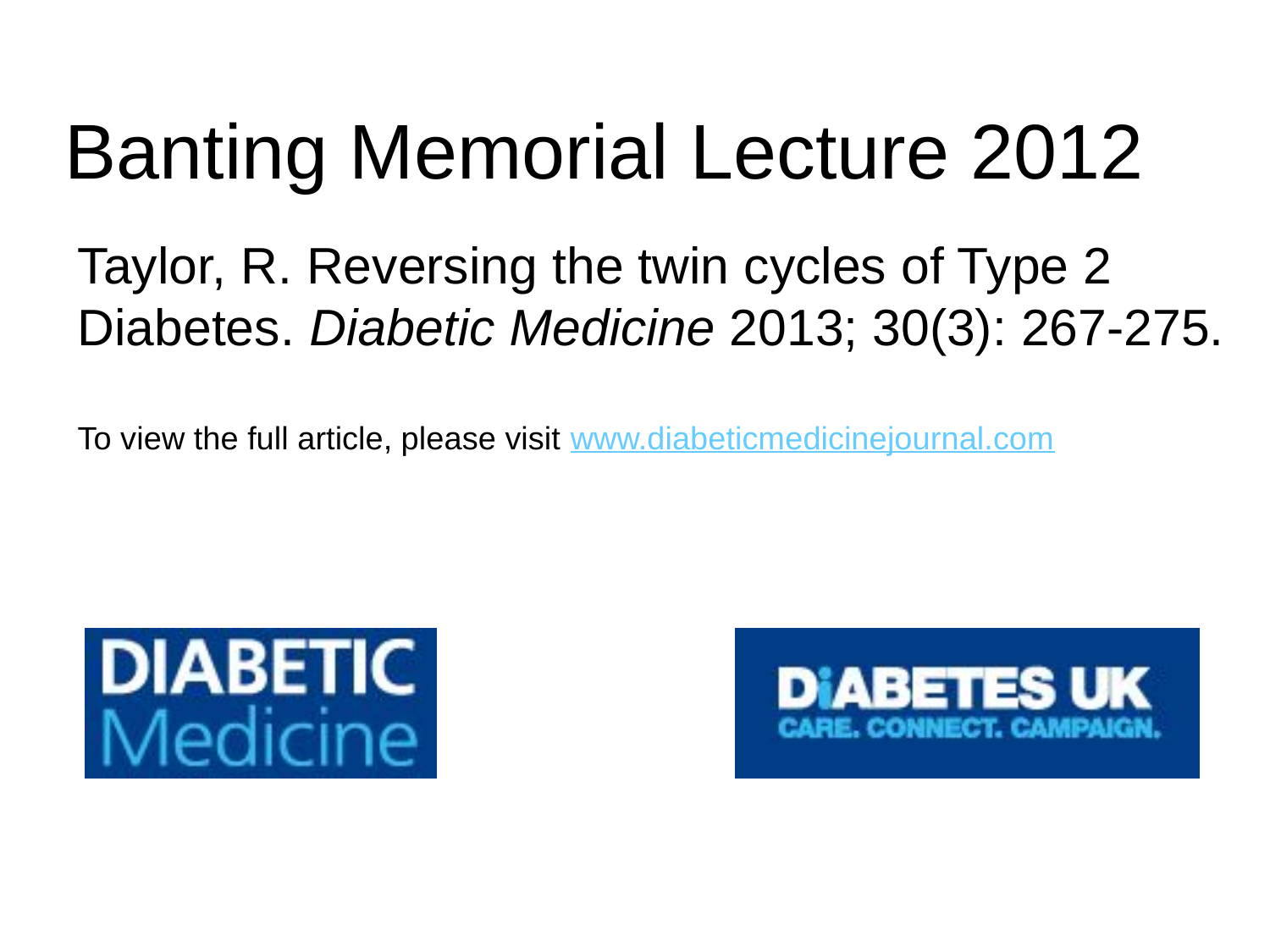

# Banting Memorial Lecture 2012
Taylor, R. Reversing the twin cycles of Type 2 Diabetes. Diabetic Medicine 2013; 30(3): 267-275.
To view the full article, please visit www.diabeticmedicinejournal.com

## Slide 32
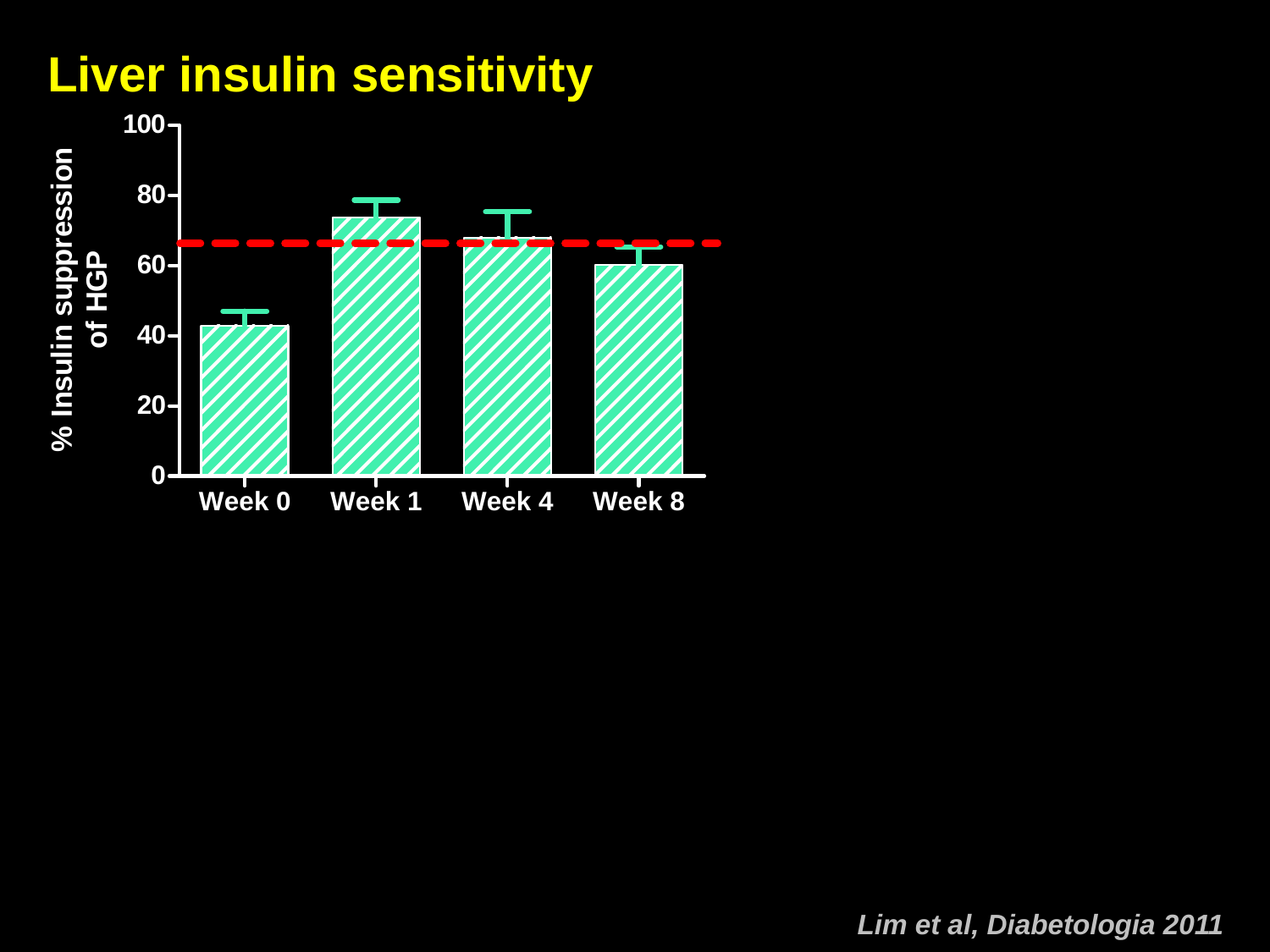

Liver insulin sensitivity
Lim et al, Diabetologia 2011

## Slide 33
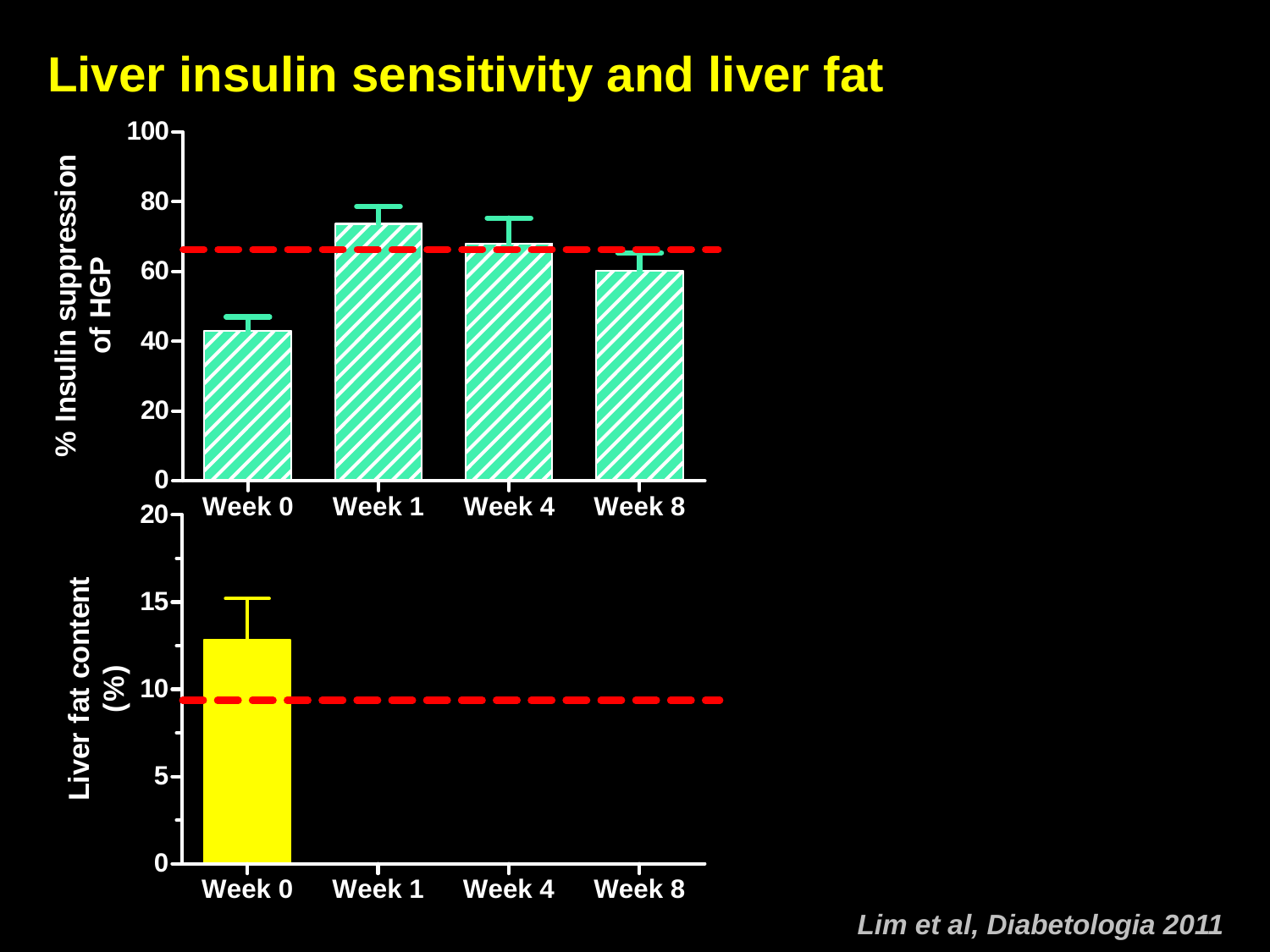

Liver insulin sensitivity and liver fat
Lim et al, Diabetologia 2011

## Slide 34
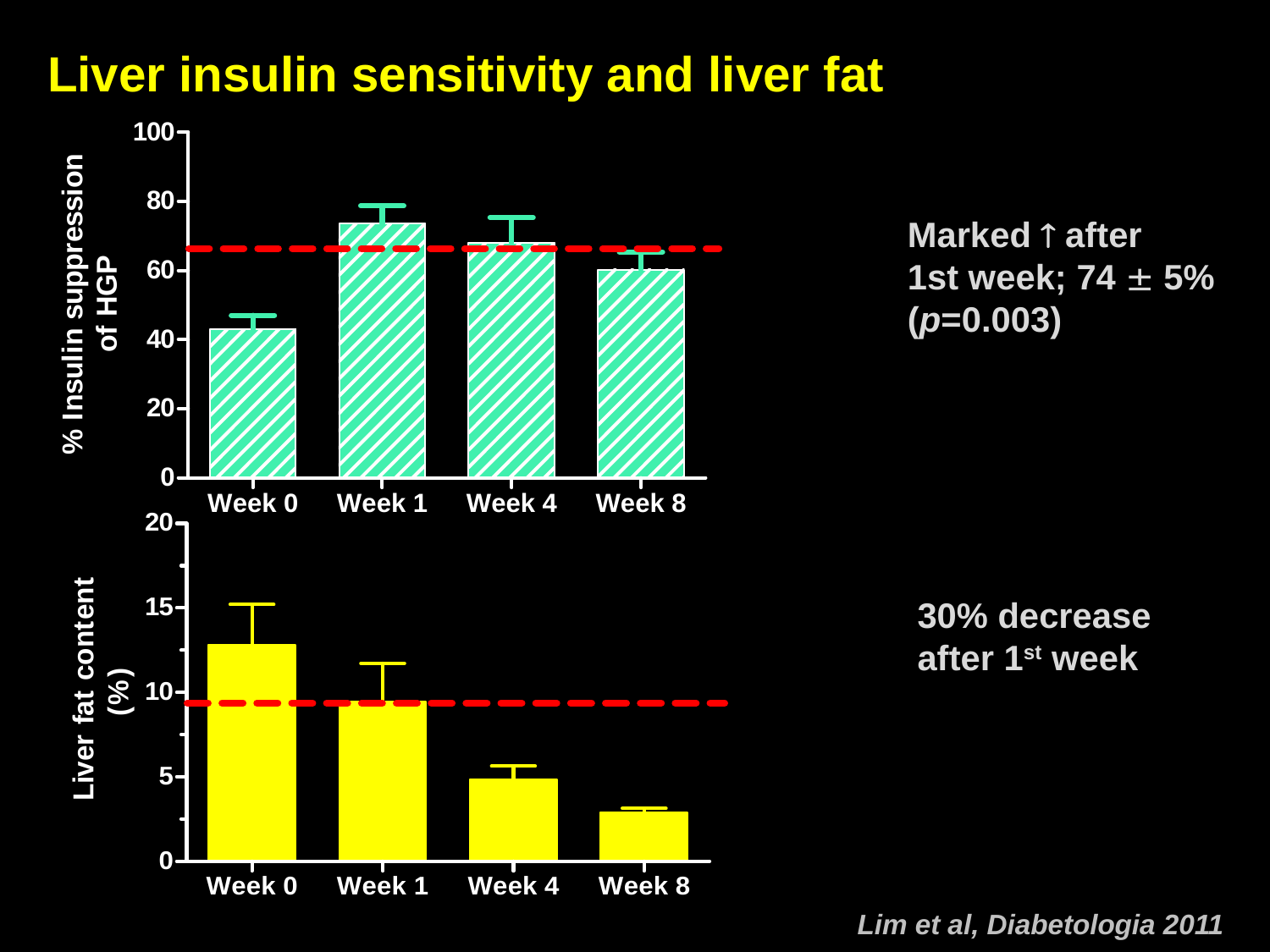

Liver insulin sensitivity and liver fat
Marked  after
1st week; 74 ± 5%
(p=0.003)
30% decrease after 1st week
Lim et al, Diabetologia 2011

## Slide 35
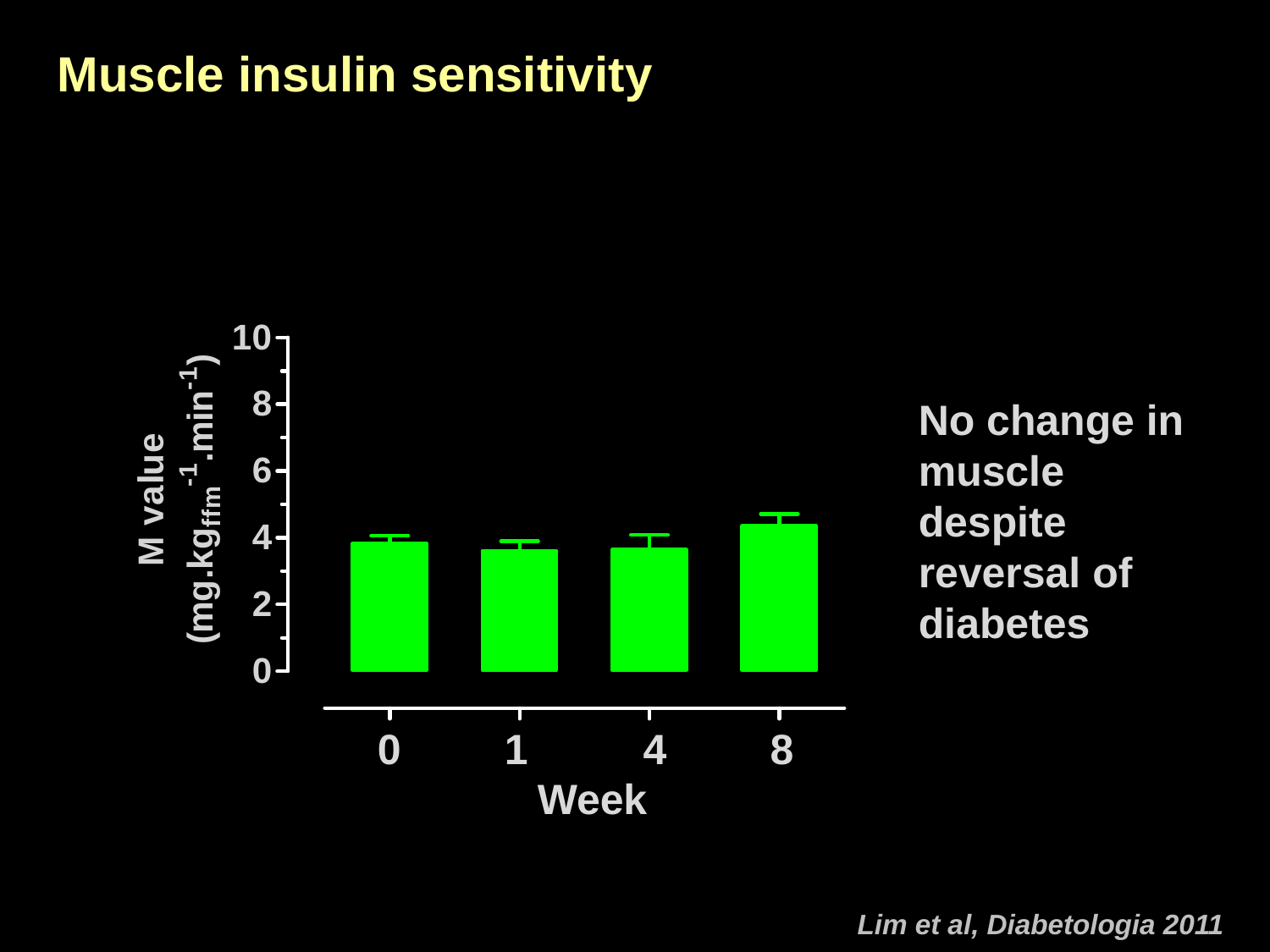

Muscle insulin sensitivity
No change in muscle despite reversal of diabetes
0 	1 	 4 	 8
Week
Lim et al, Diabetologia 2011

## Slide 36
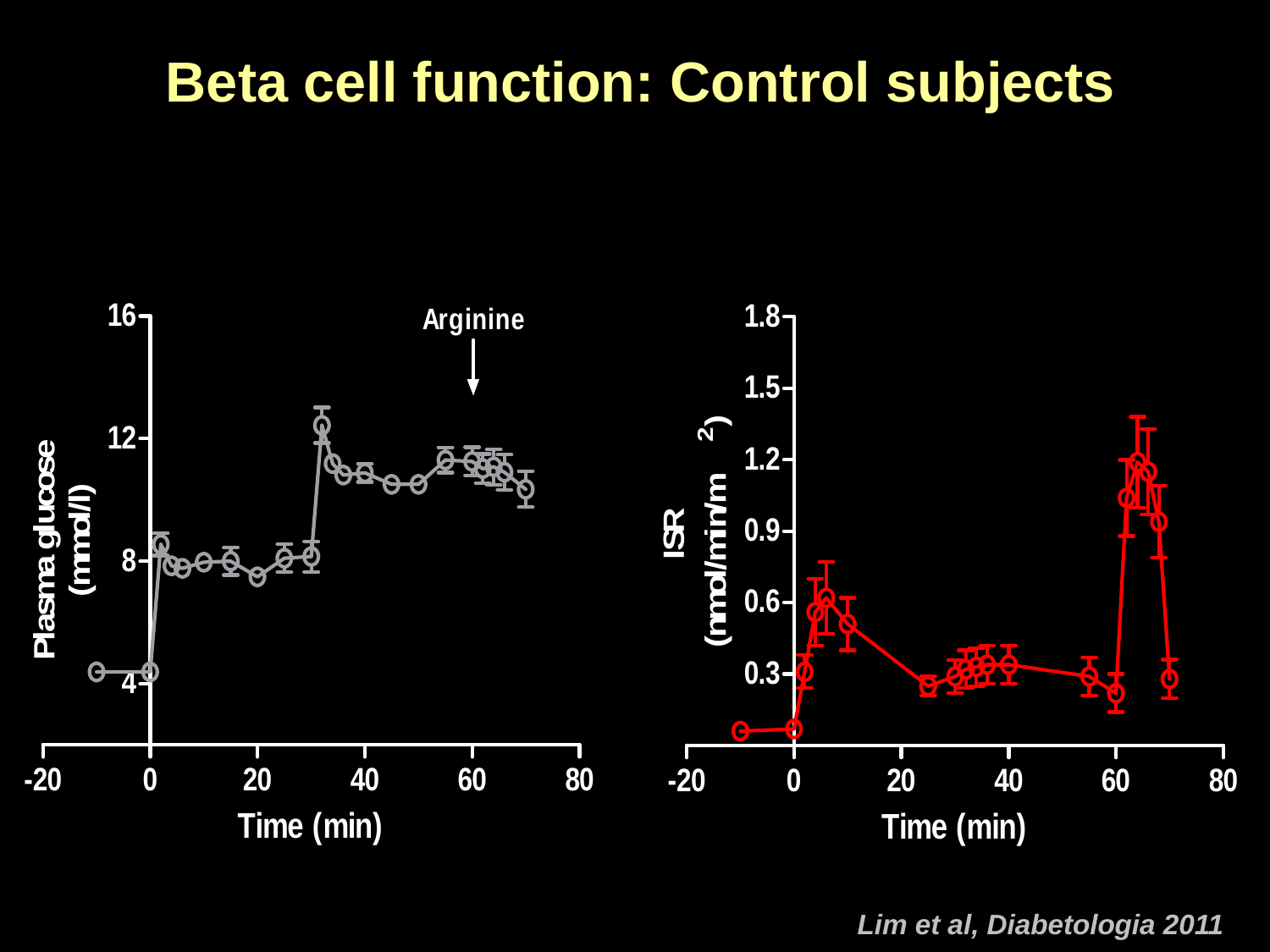

# Beta cell function: Control subjects
Lim et al, Diabetologia 2011

## Slide 37
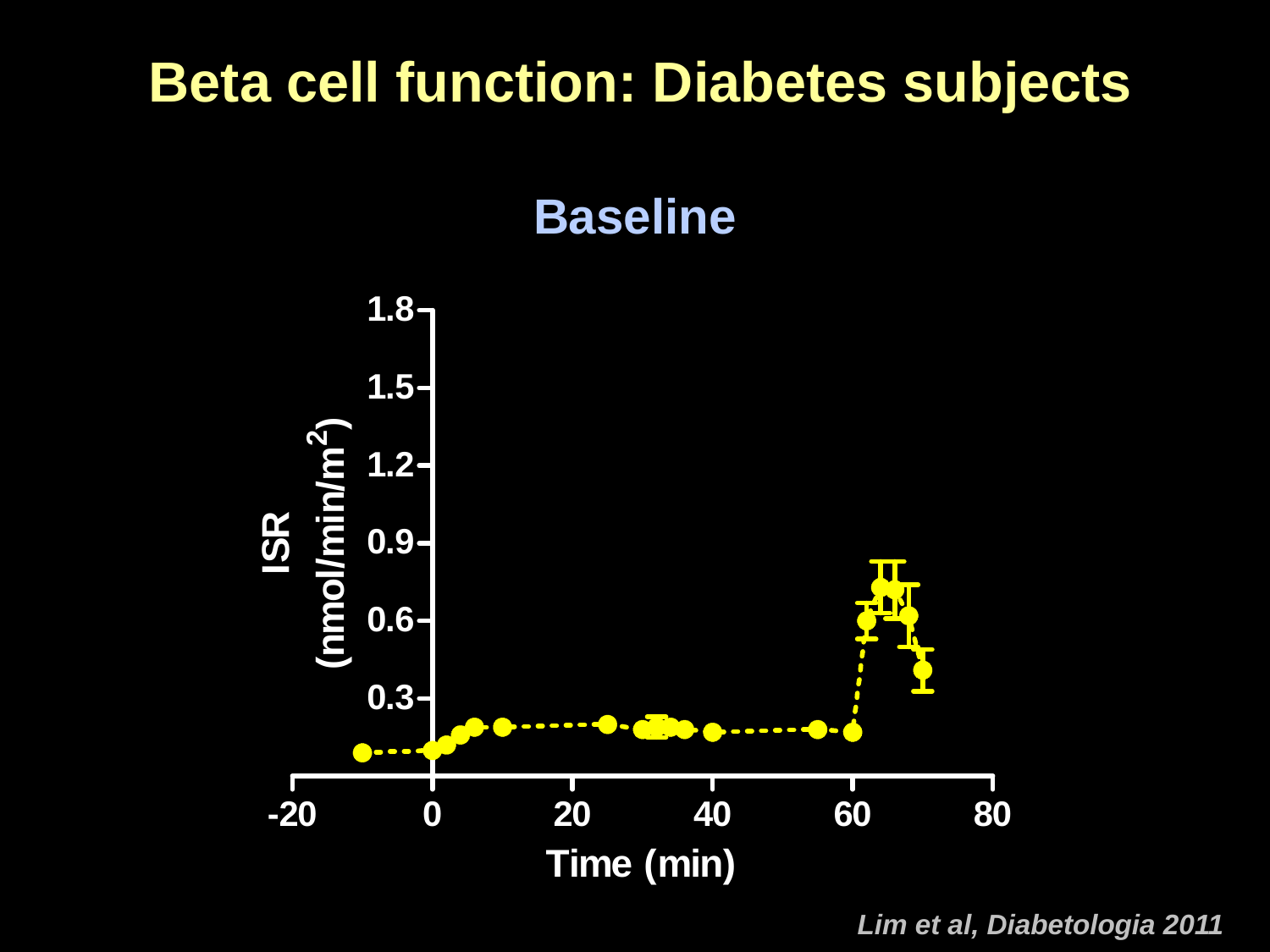

# Beta cell function: Diabetes subjects
Baseline
Lim et al, Diabetologia 2011

## Slide 38
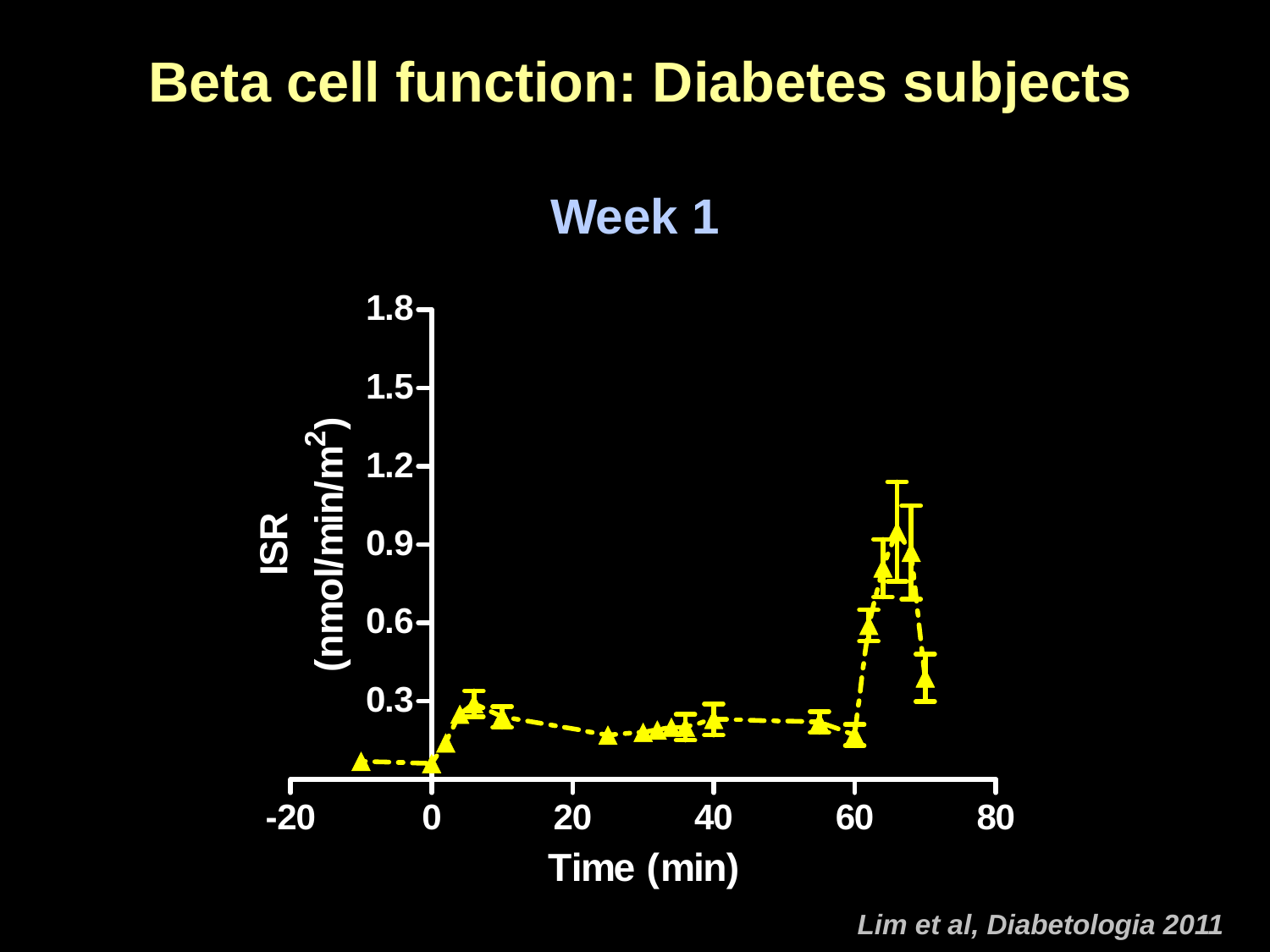

# Beta cell function: Diabetes subjects
Week 1
Lim et al, Diabetologia 2011

## Slide 39
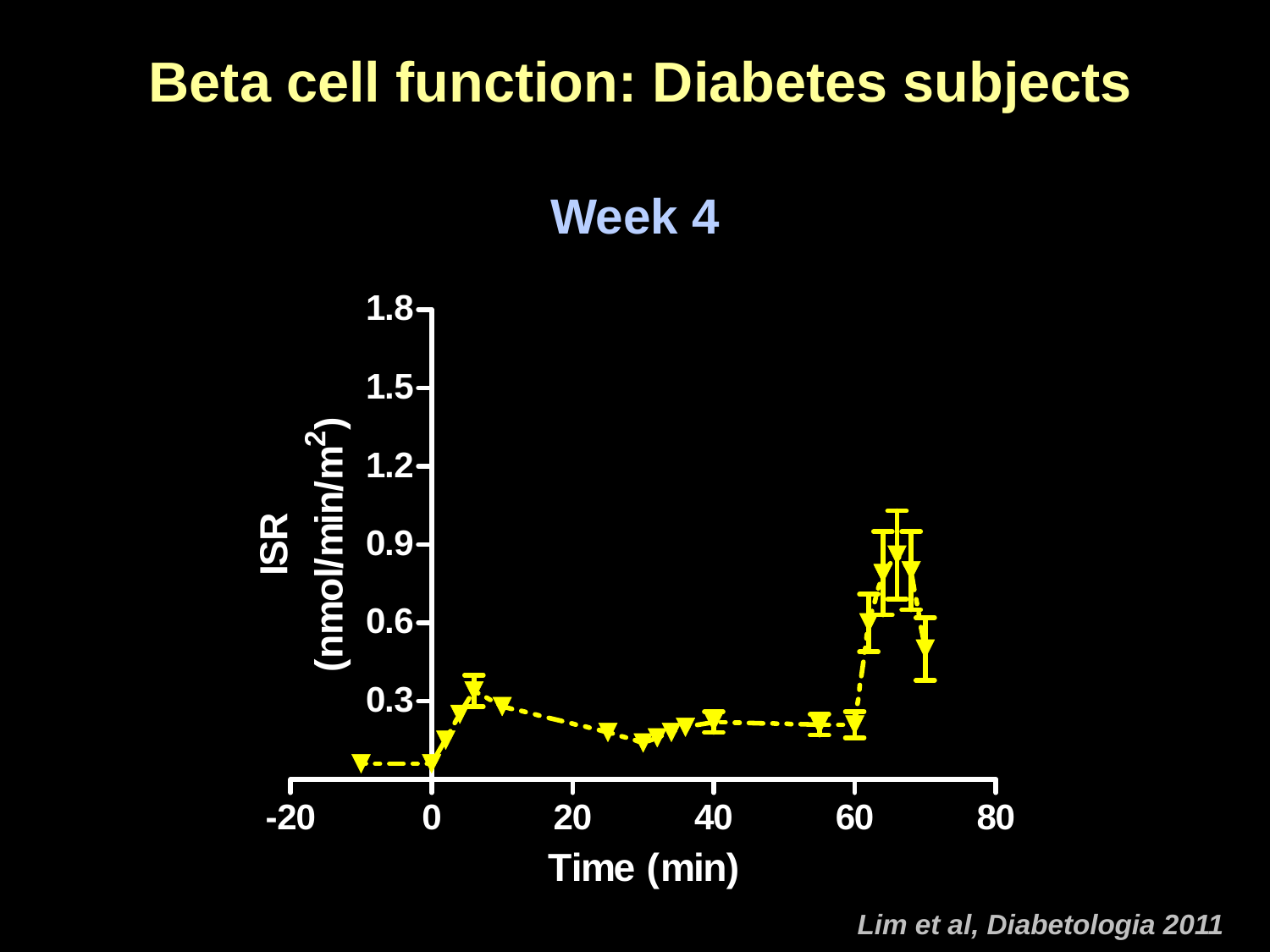

# Beta cell function: Diabetes subjects
Week 4
Lim et al, Diabetologia 2011

## Slide 40
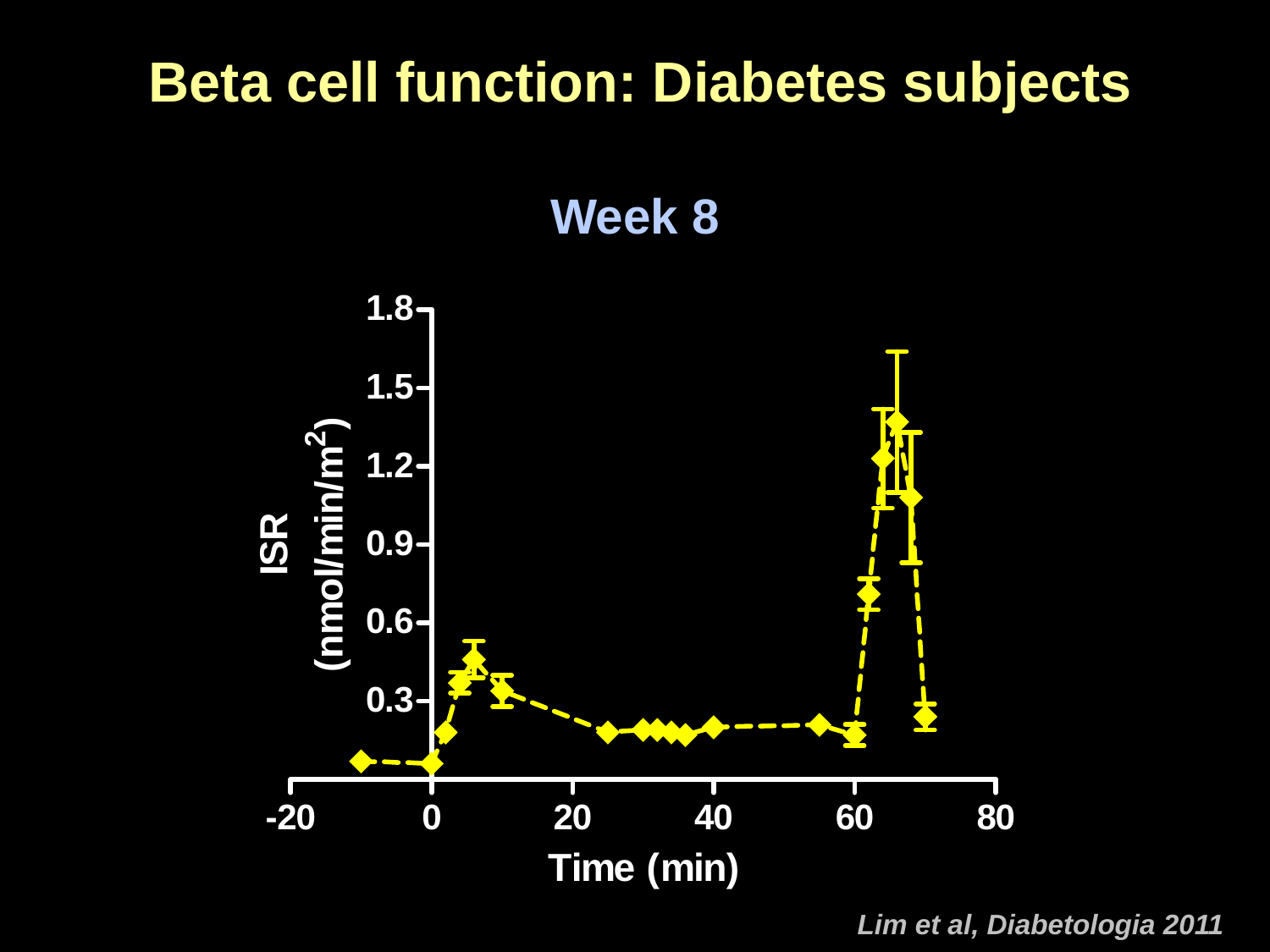

# Beta cell function: Diabetes subjects
Week 8
Lim et al, Diabetologia 2011

## Slide 41
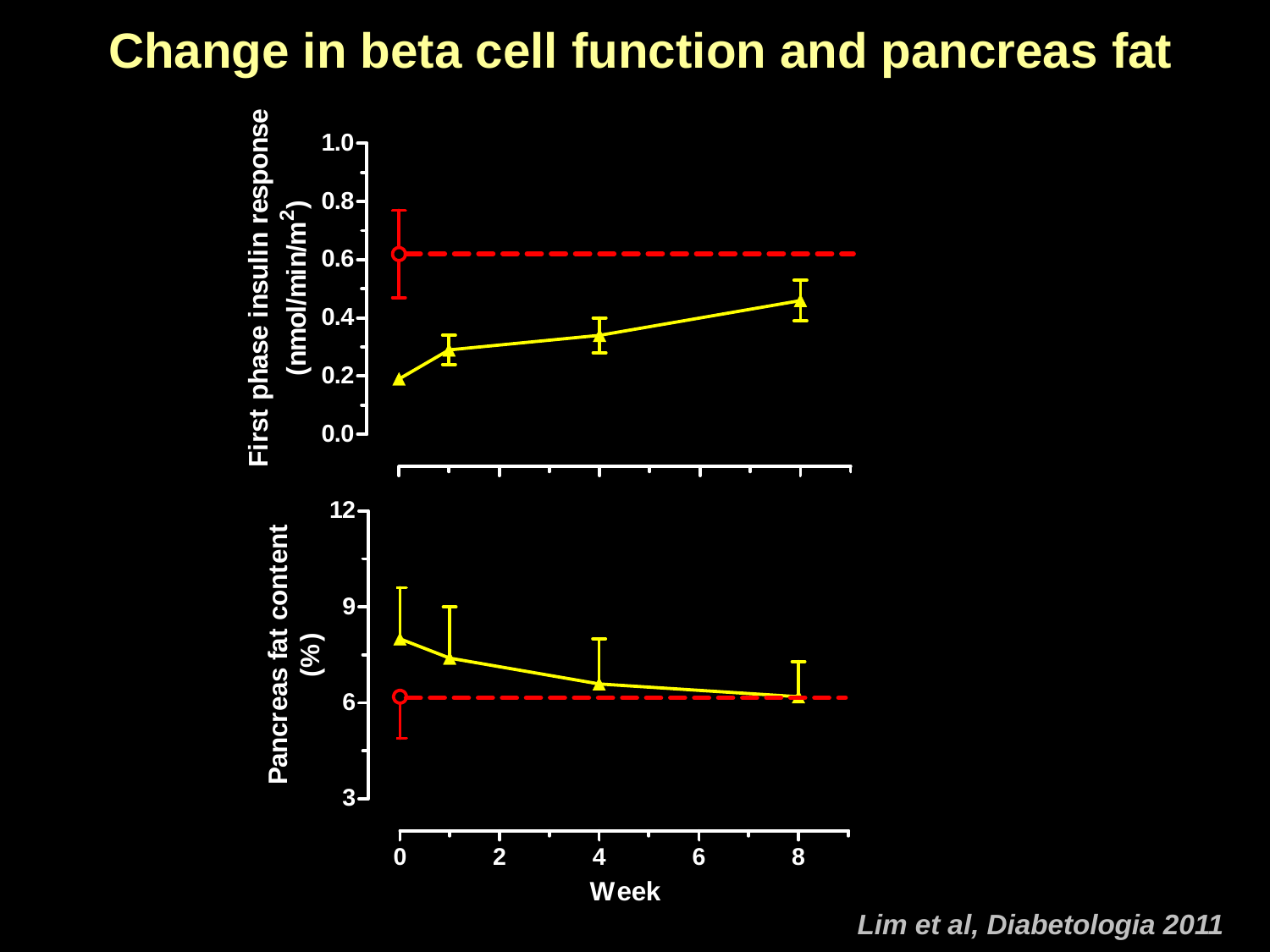

# Change in beta cell function and pancreas fat
Lim et al, Diabetologia 2011

## Slide 42
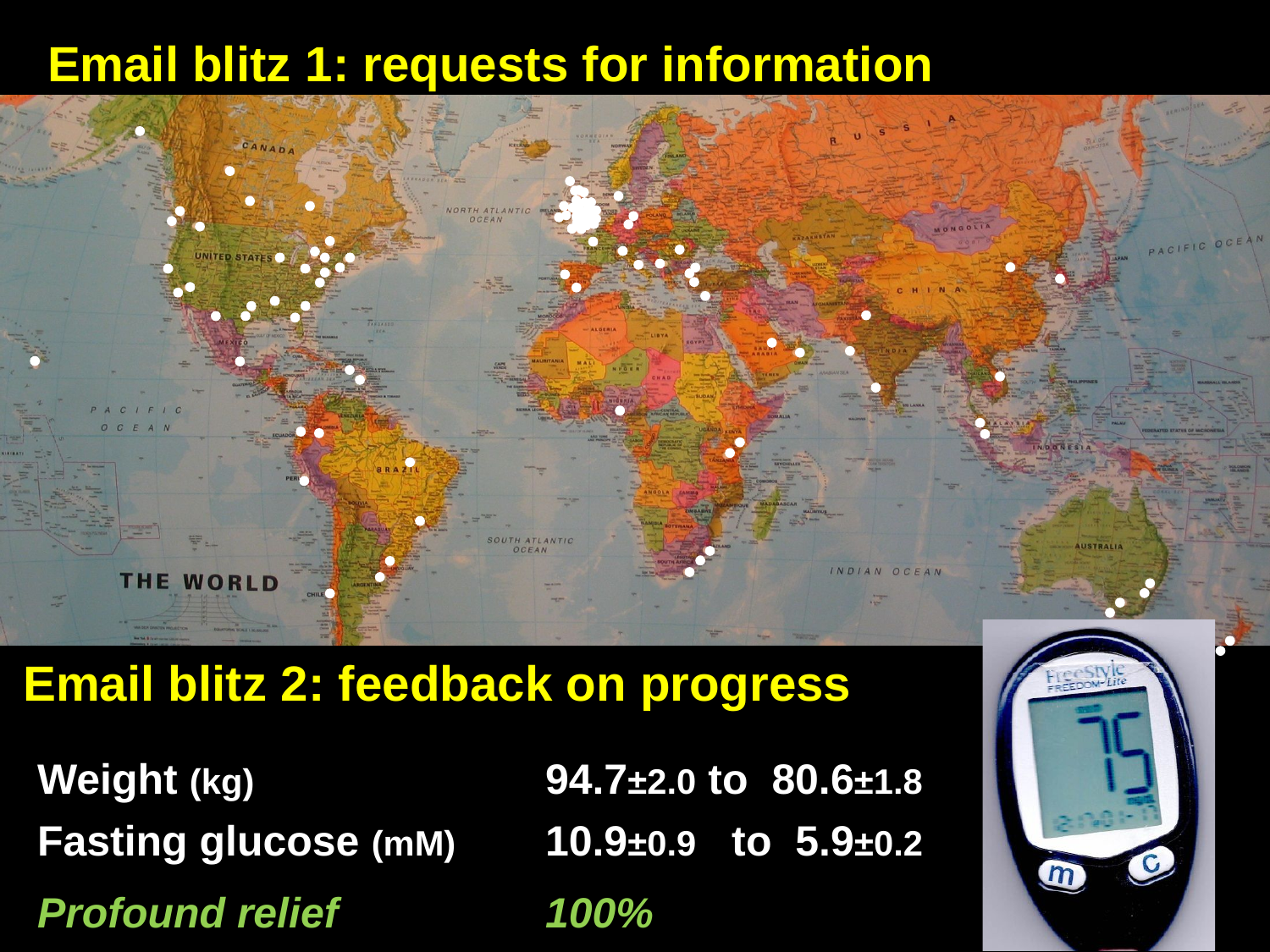

Email blitz 1: requests for information
1,000 email FU
Email blitz 2: feedback on progress
Weight (kg)			94.7±2.0 to 80.6±1.8
Fasting glucose (mM)	10.9±0.9 to 5.9±0.2
Profound relief 		100%

## Slide 43
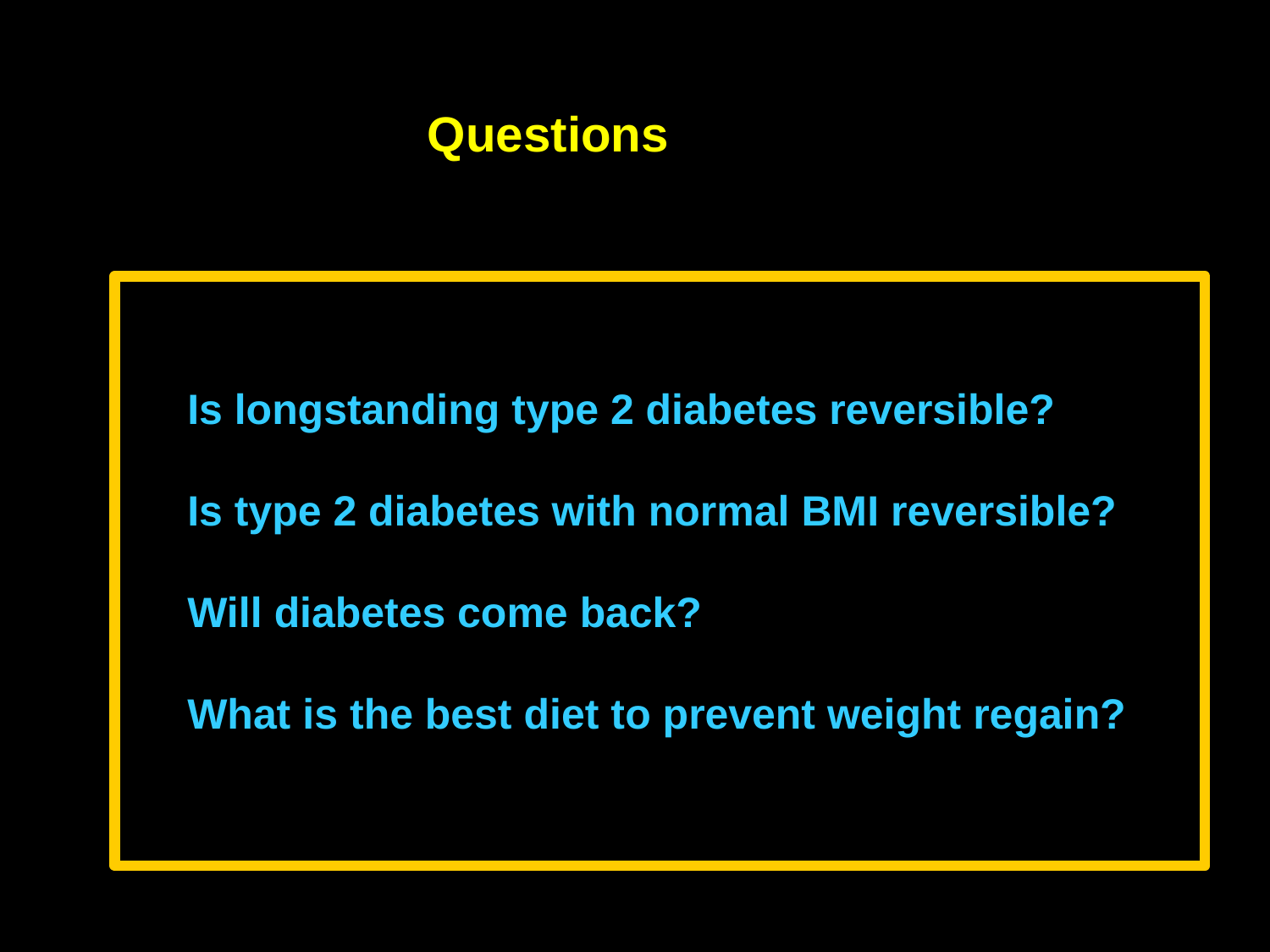

Questions
Is longstanding type 2 diabetes reversible?
Is type 2 diabetes with normal BMI reversible?
Will diabetes come back?
What is the best diet to prevent weight regain?

## Slide 44
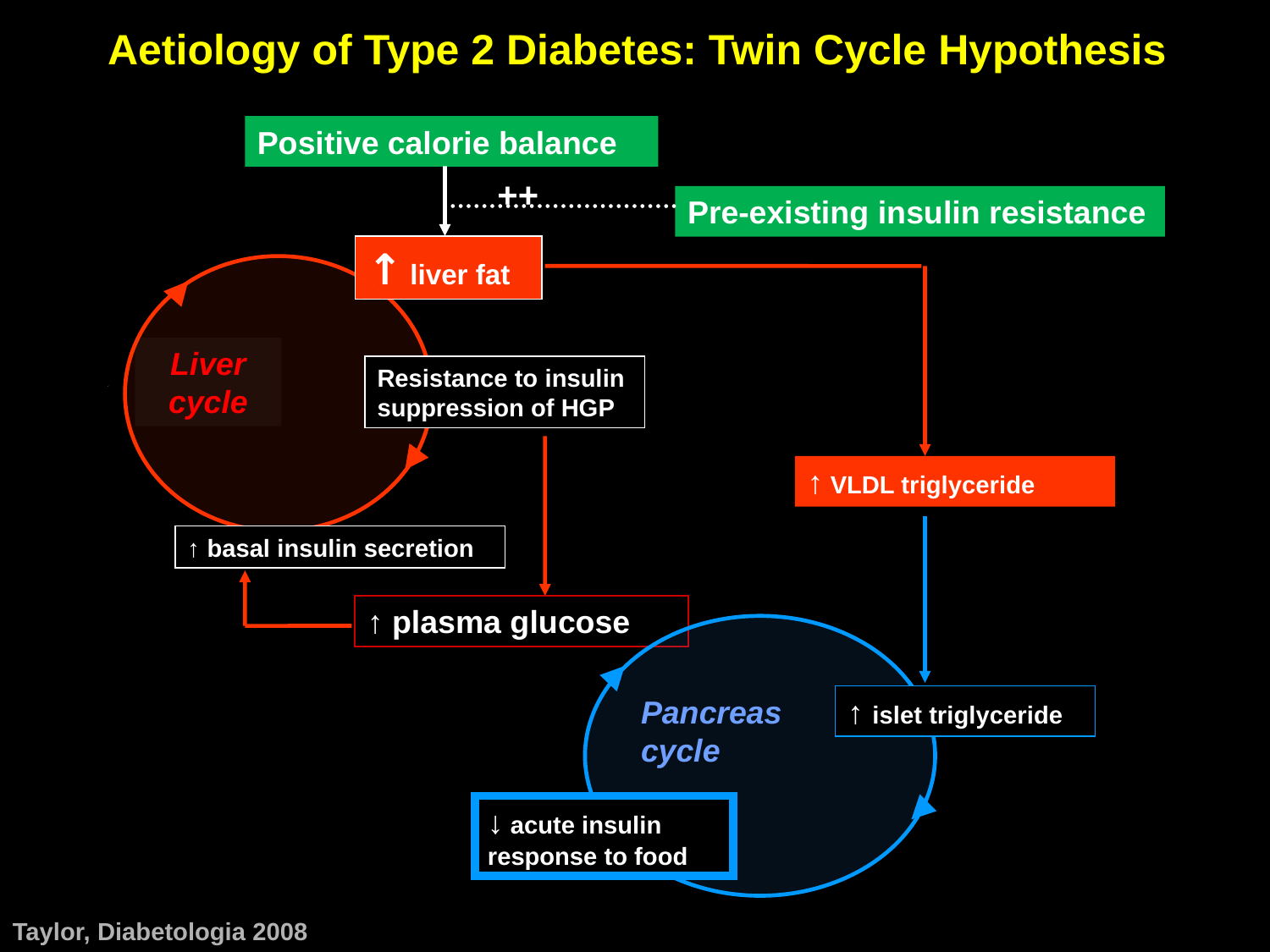

Aetiology of Type 2 Diabetes: Twin Cycle Hypothesis
Positive calorie balance
++
Pre-existing insulin resistance
↑ liver fat
Liver cycle
Resistance to insulin suppression of HGP
↑ VLDL triglyceride
↑ basal insulin secretion
↑ plasma glucose
Pancreas cycle
↑ islet triglyceride
↓ acute insulin response to food
Taylor, Diabetologia 2008

## Slide 45
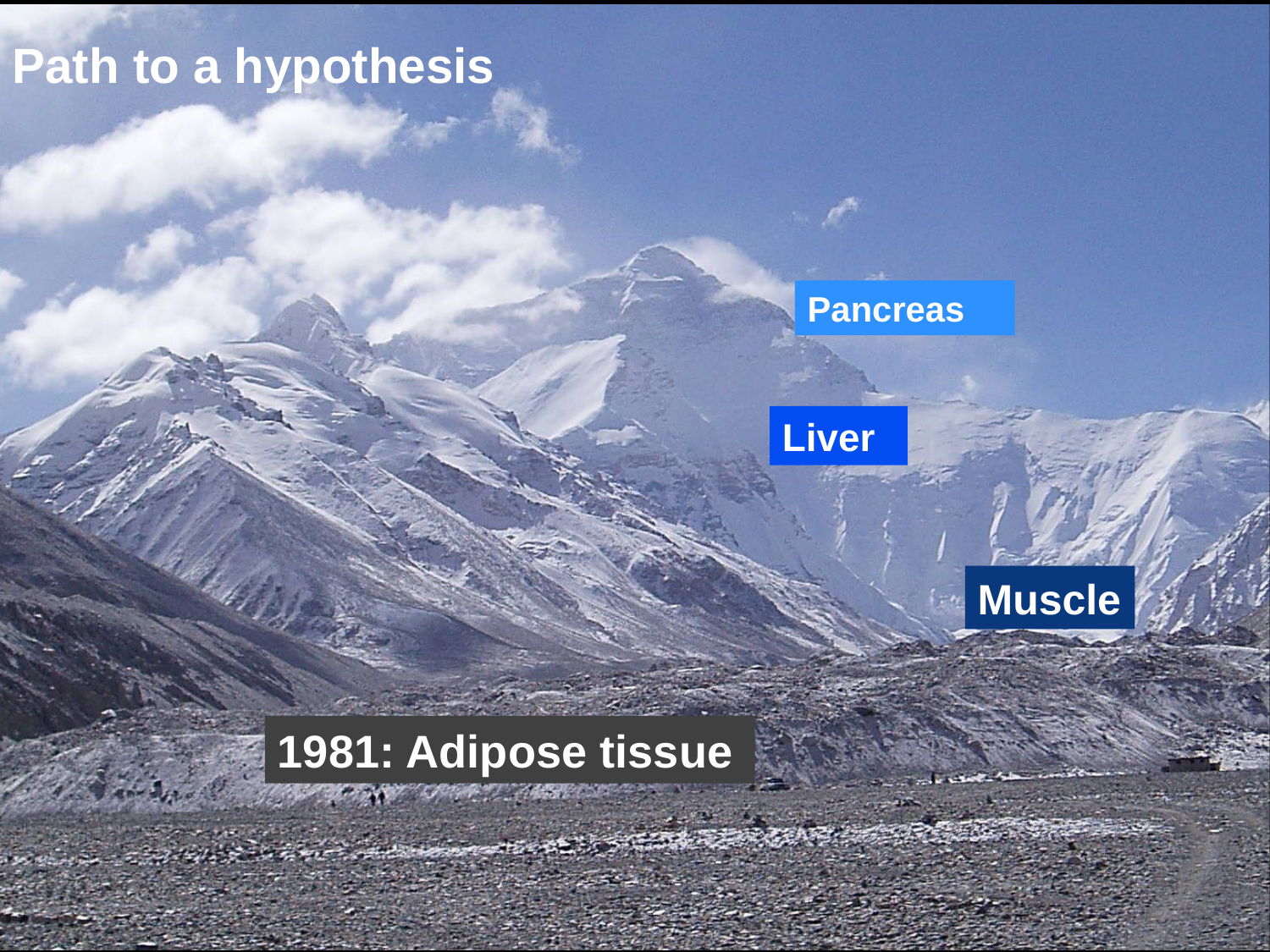

Path to a hypothesis
Pancreas
Liver
Muscle
1981: Adipose tissue

## Slide 46
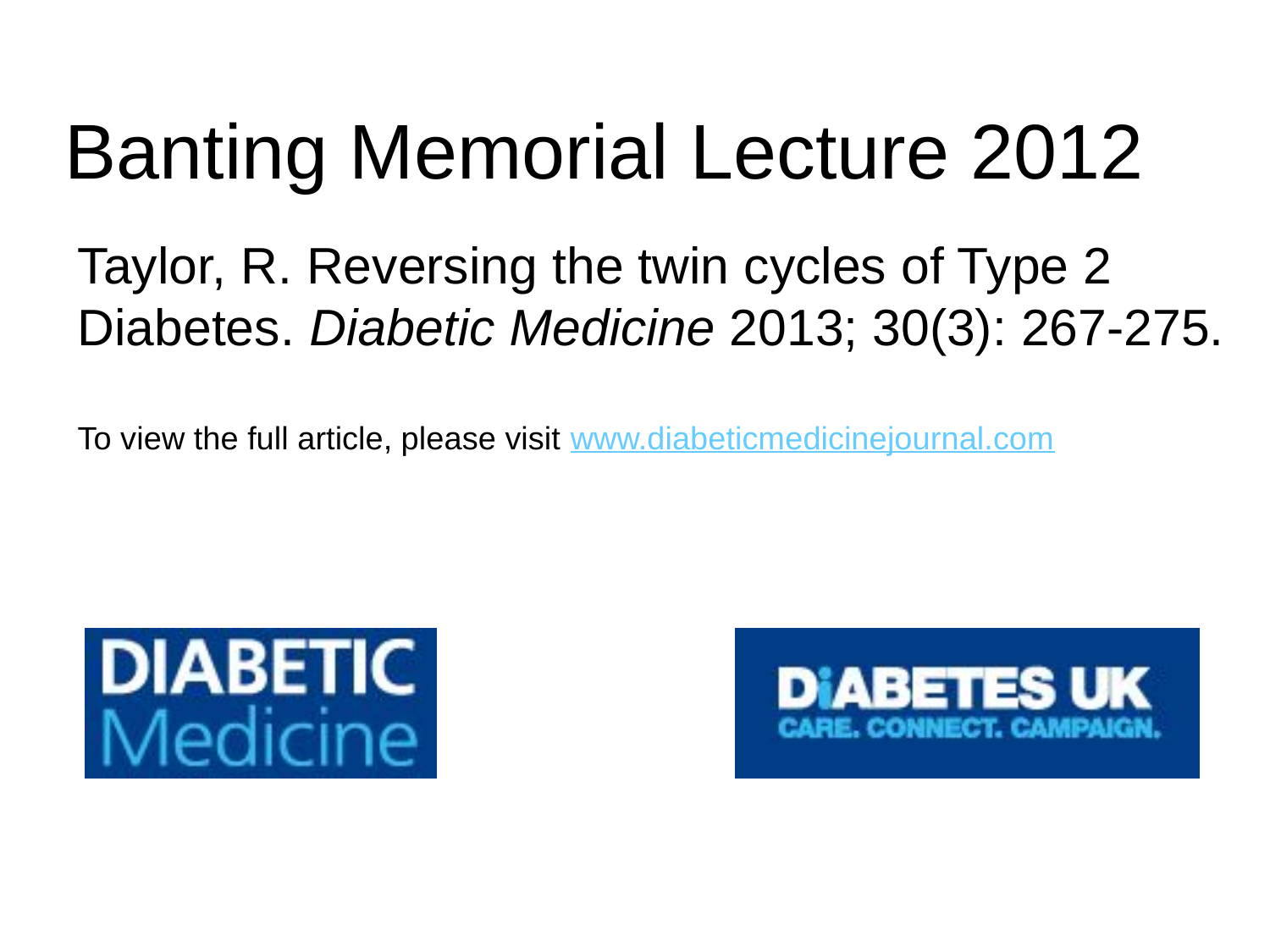

# Banting Memorial Lecture 2012
Taylor, R. Reversing the twin cycles of Type 2 Diabetes. Diabetic Medicine 2013; 30(3): 267-275.
To view the full article, please visit www.diabeticmedicinejournal.com

## Slide 47
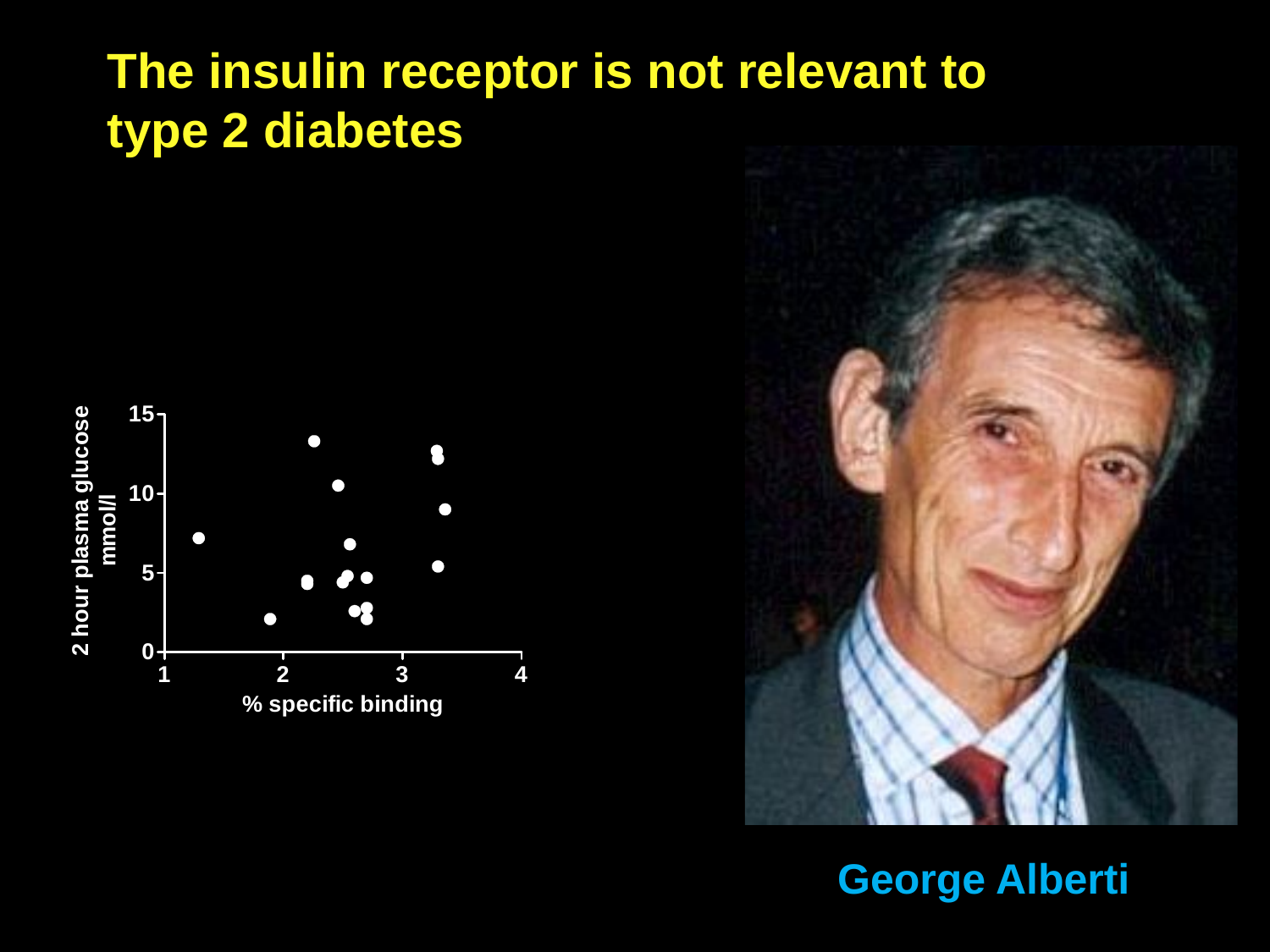

The insulin receptor is not relevant to type 2 diabetes
George Alberti

## Slide 48
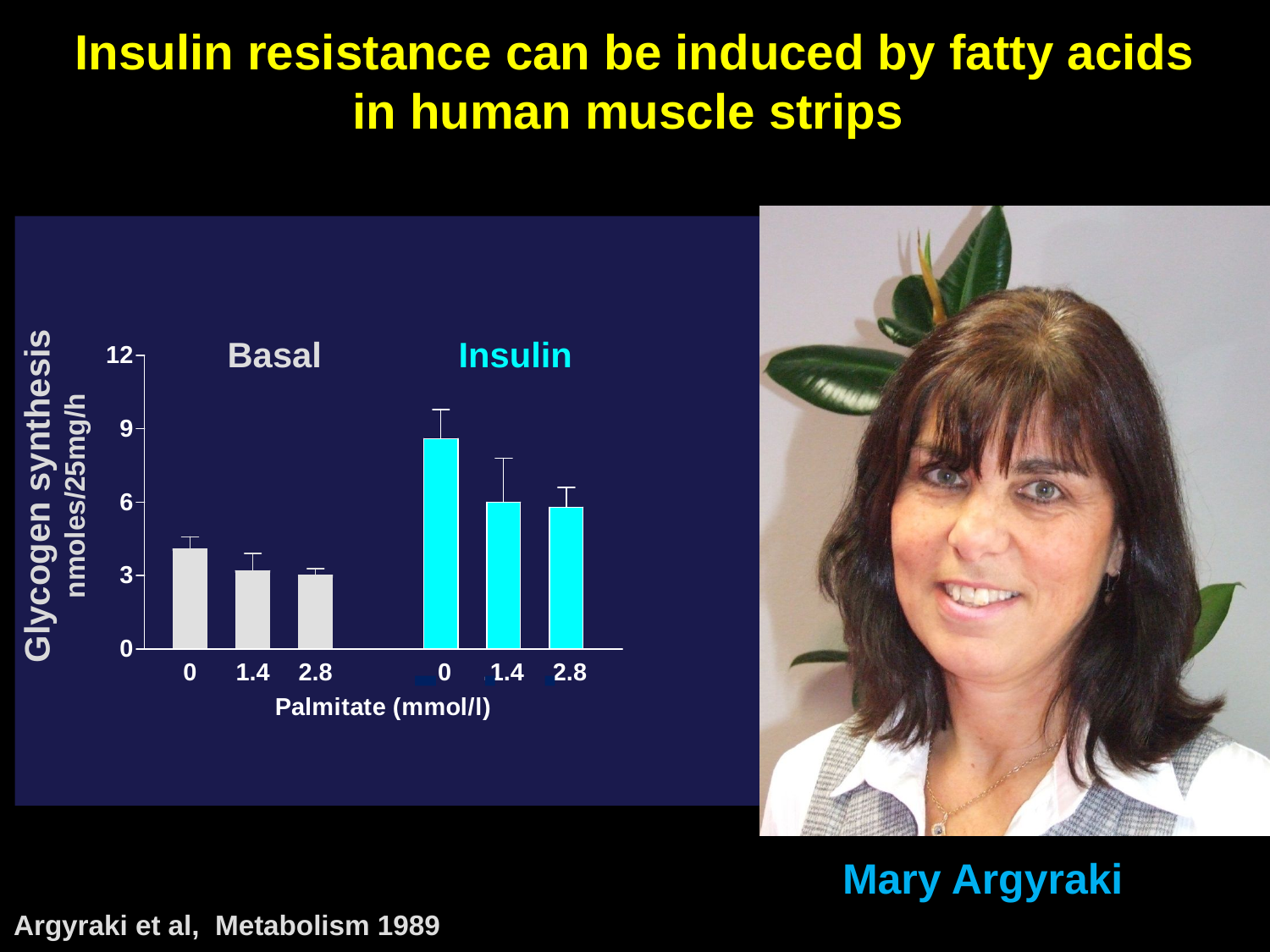

# Insulin resistance can be induced by fatty acids in human muscle strips
Basal
Insulin
Glycogen synthesis
nmoles/25mg/h
Mary Argyraki
Argyraki et al, Metabolism 1989

## Slide 49
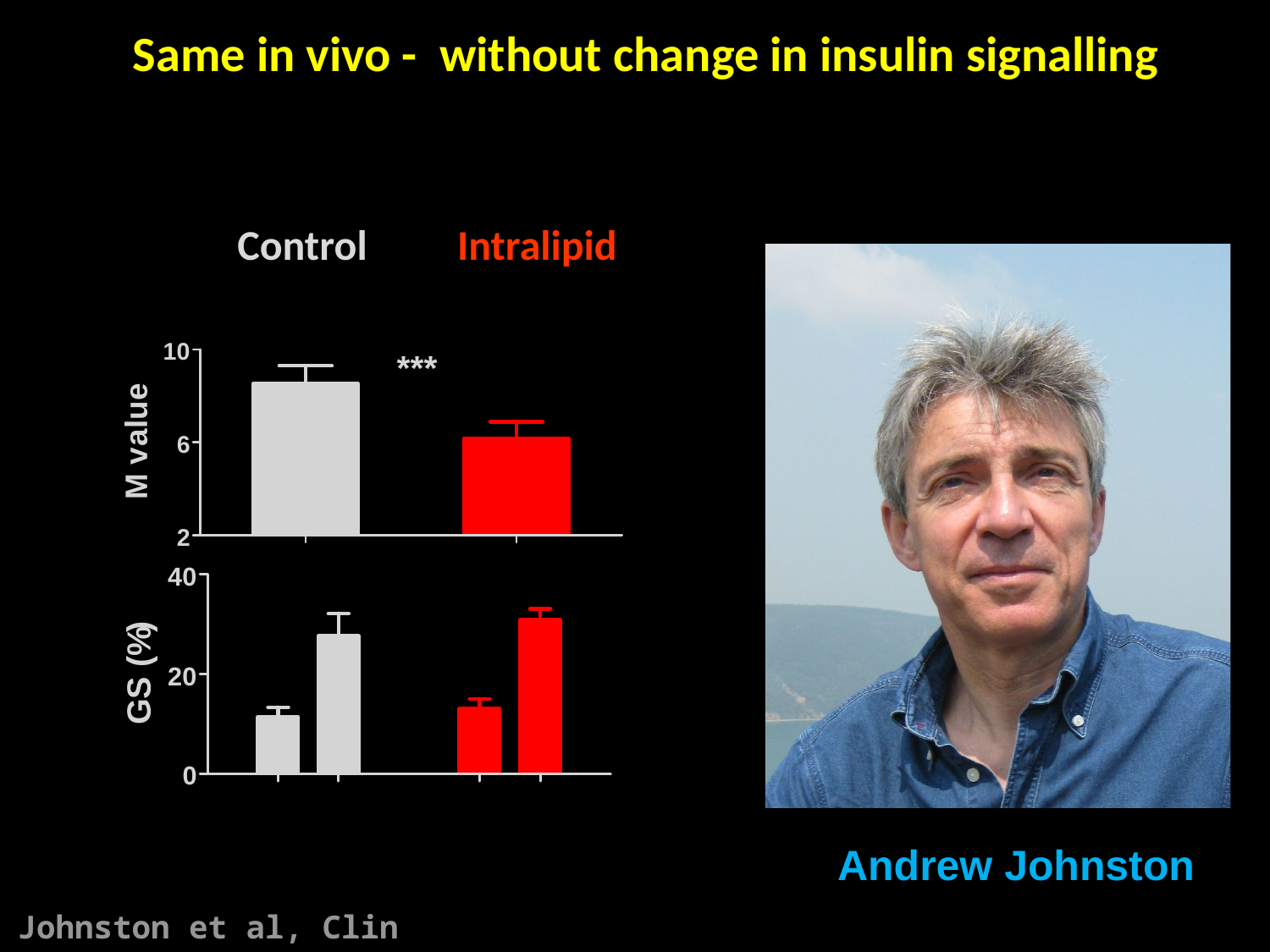

Same in vivo - without change in insulin signalling
Control
Intralipid
Andrew Johnston
Johnston et al, Clin Sci 1996

## Slide 50
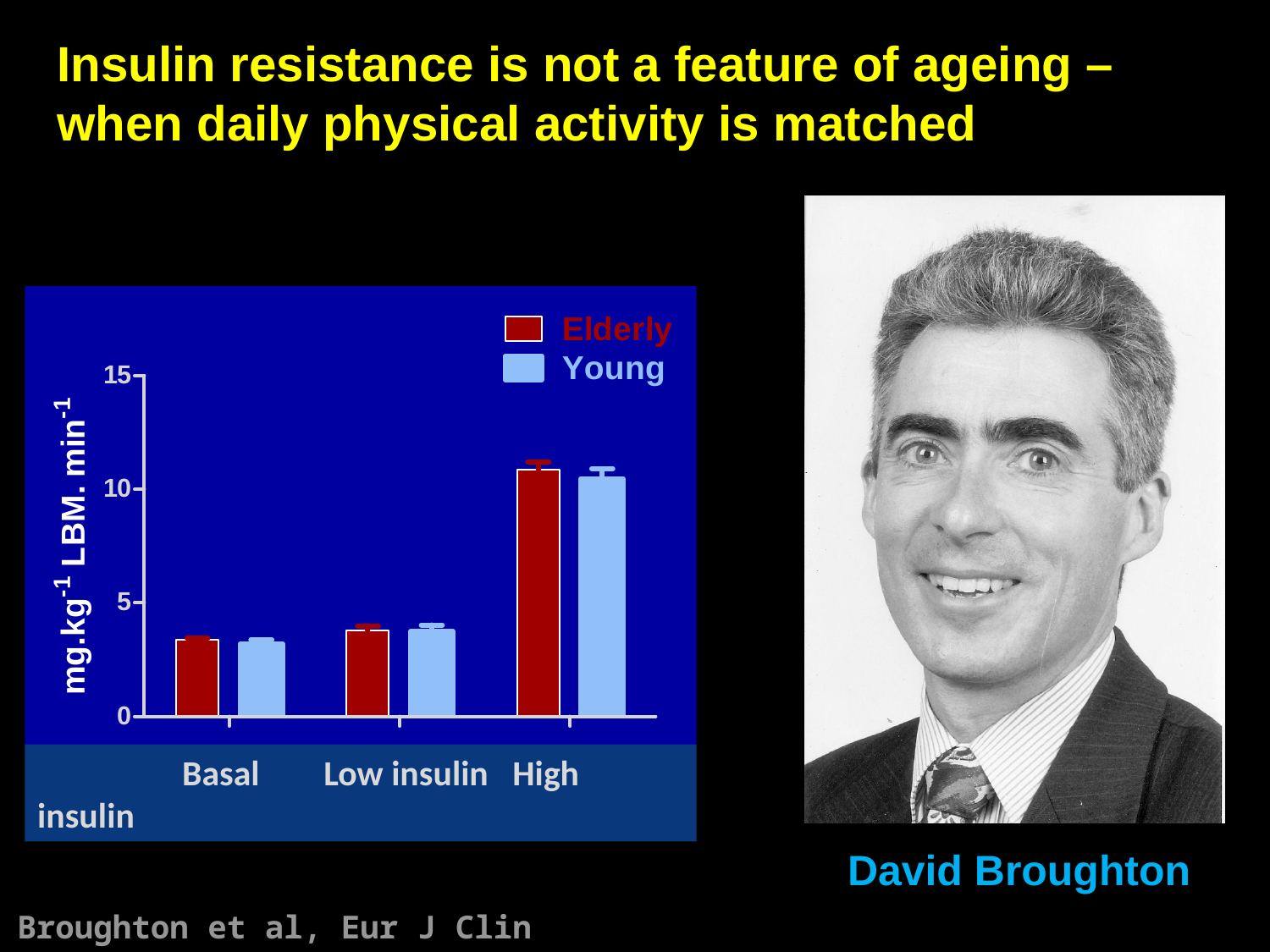

Insulin resistance is not a feature of ageing – when daily physical activity is matched
 Basal Low insulin High insulin
David Broughton
Broughton et al, Eur J Clin Invest 1991

## Slide 51
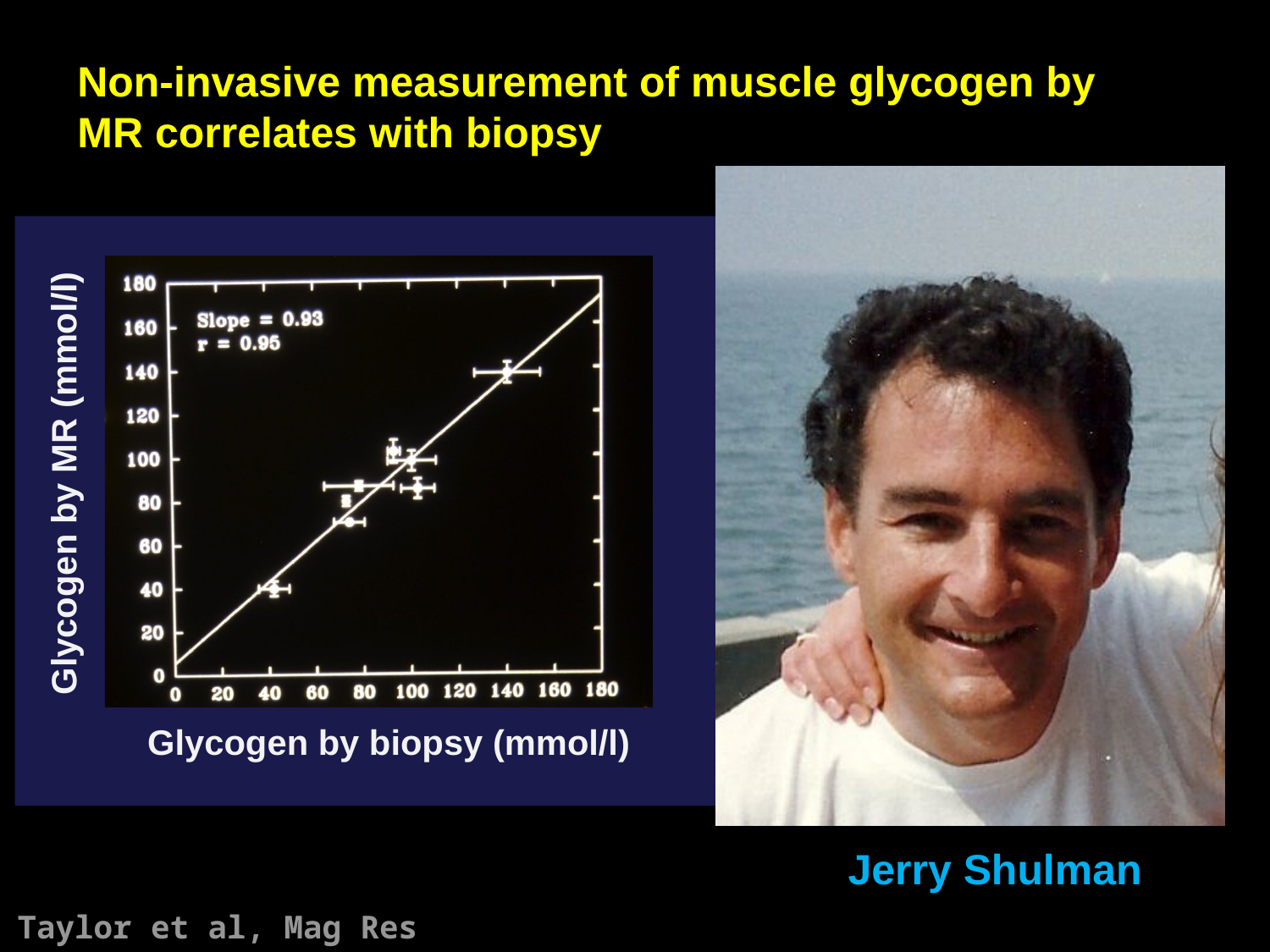

Non-invasive measurement of muscle glycogen by MR correlates with biopsy
Glycogen by MR (mmol/l)
Glycogen by biopsy (mmol/l)
Jerry Shulman
Taylor et al, Mag Res Med 1992

## Slide 52
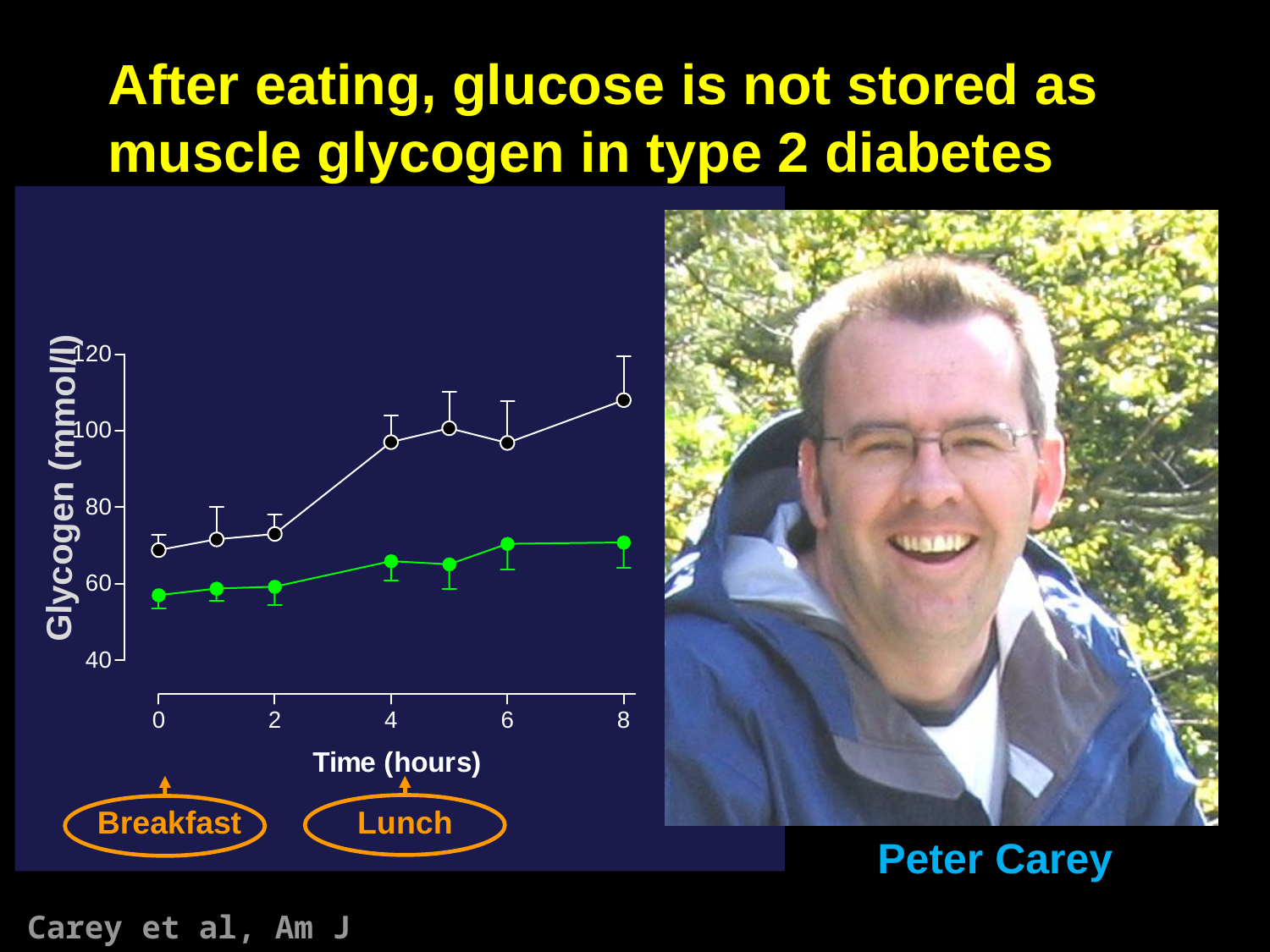

After eating, glucose is not stored as muscle glycogen in type 2 diabetes
Glycogen (mmol/l)
Breakfast
Lunch
Peter Carey
Carey et al, Am J Physiol 2002

## Slide 53
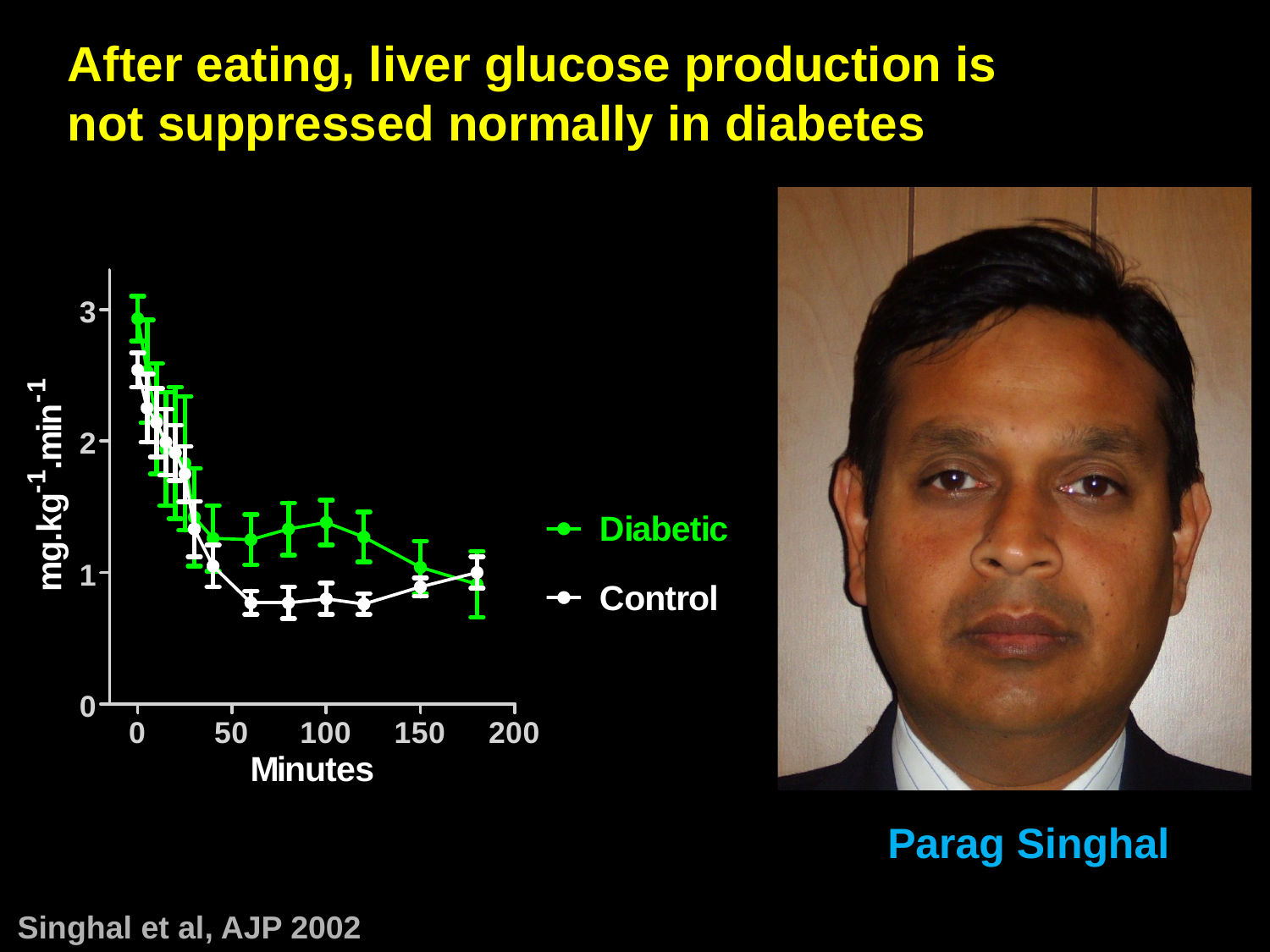

After eating, liver glucose production is not suppressed normally in diabetes
Parag Singhal
Singhal et al, AJP 2002

## Slide 54
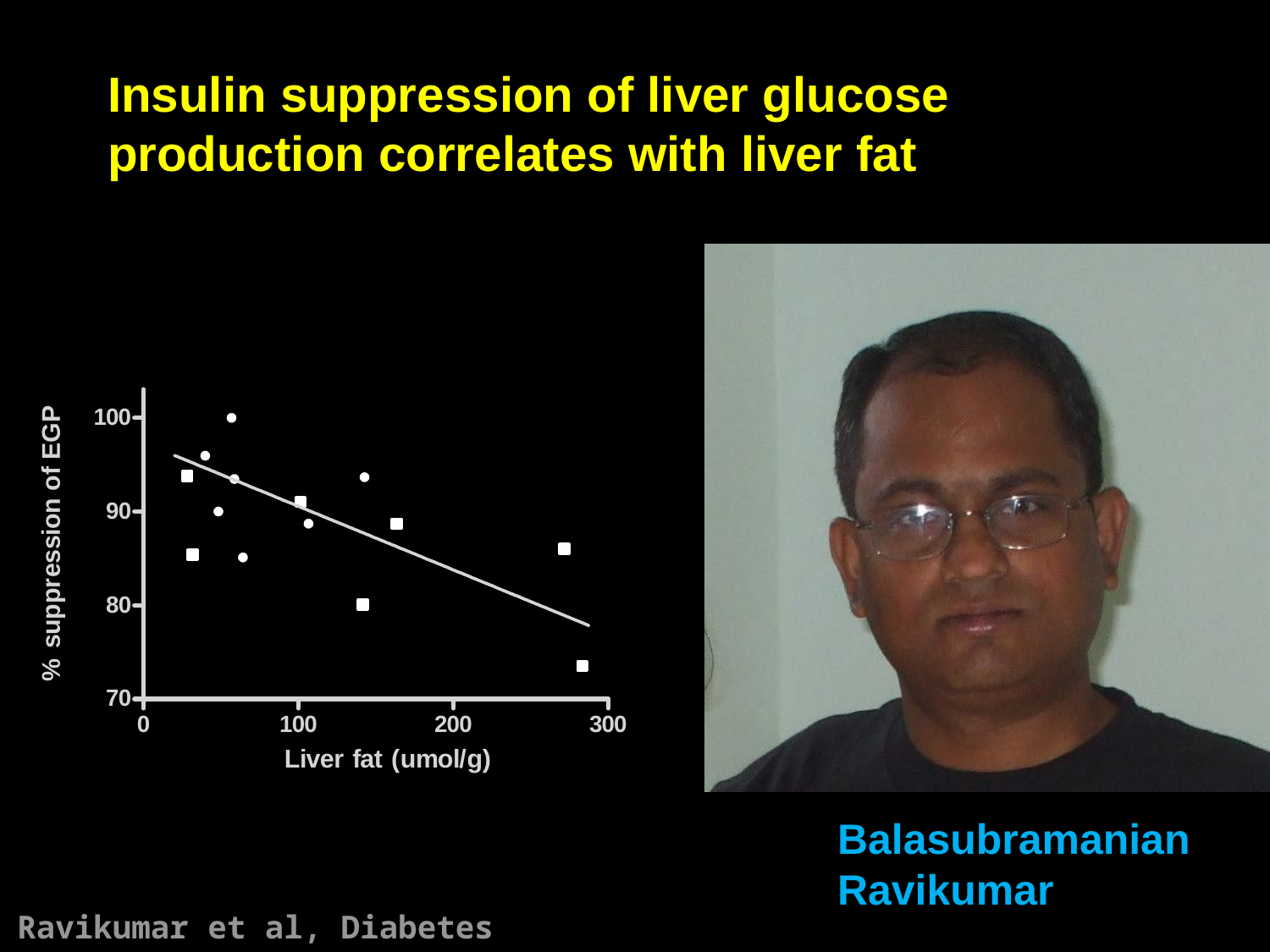

Insulin suppression of liver glucose production correlates with liver fat
Balasubramanian Ravikumar
Ravikumar et al, Diabetes 2008

## Slide 55
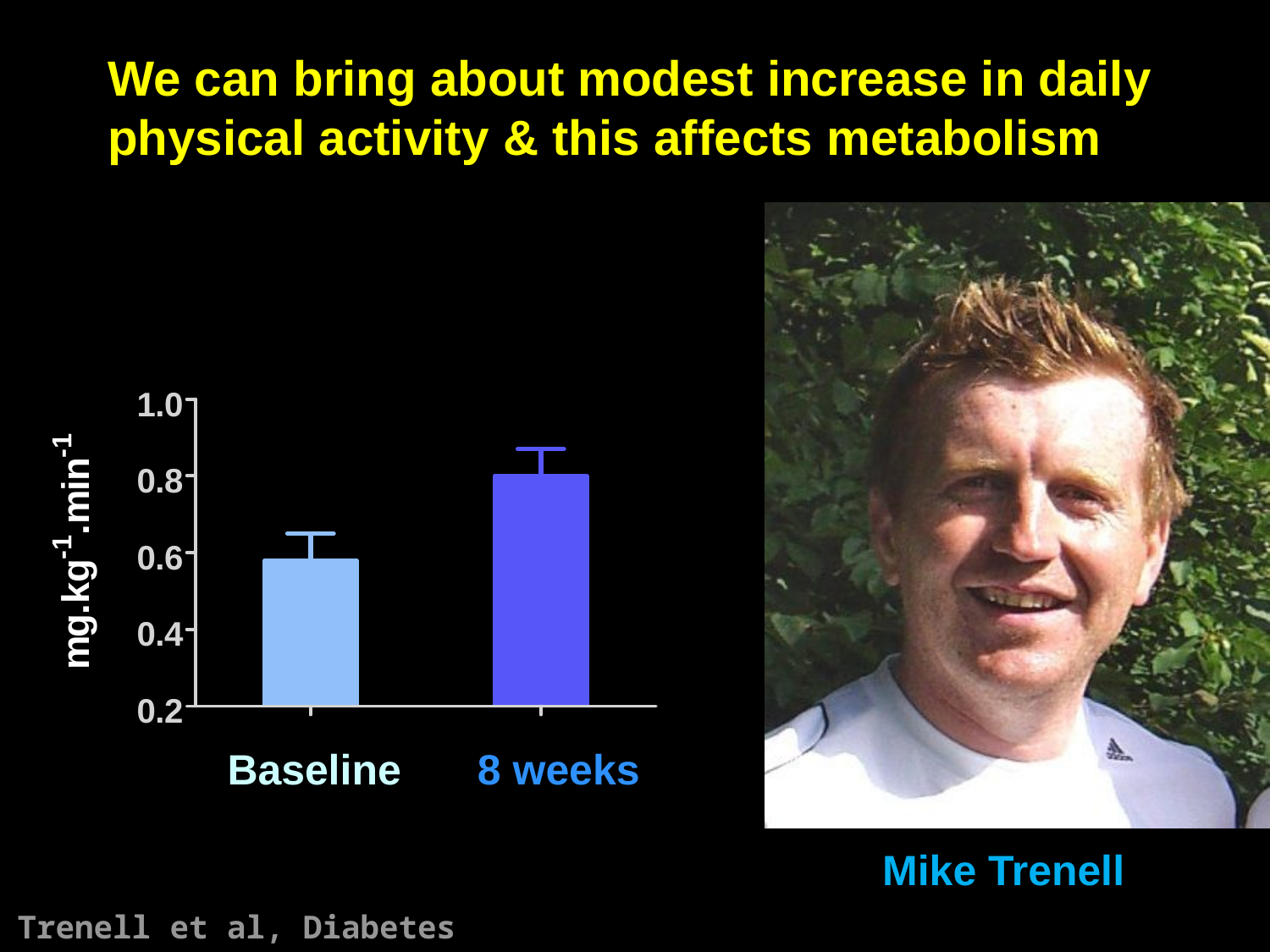

Results
We can bring about modest increase in daily physical activity & this affects metabolism
Baseline
8 weeks
Mike Trenell
Trenell et al, Diabetes Care 2008

## Slide 56
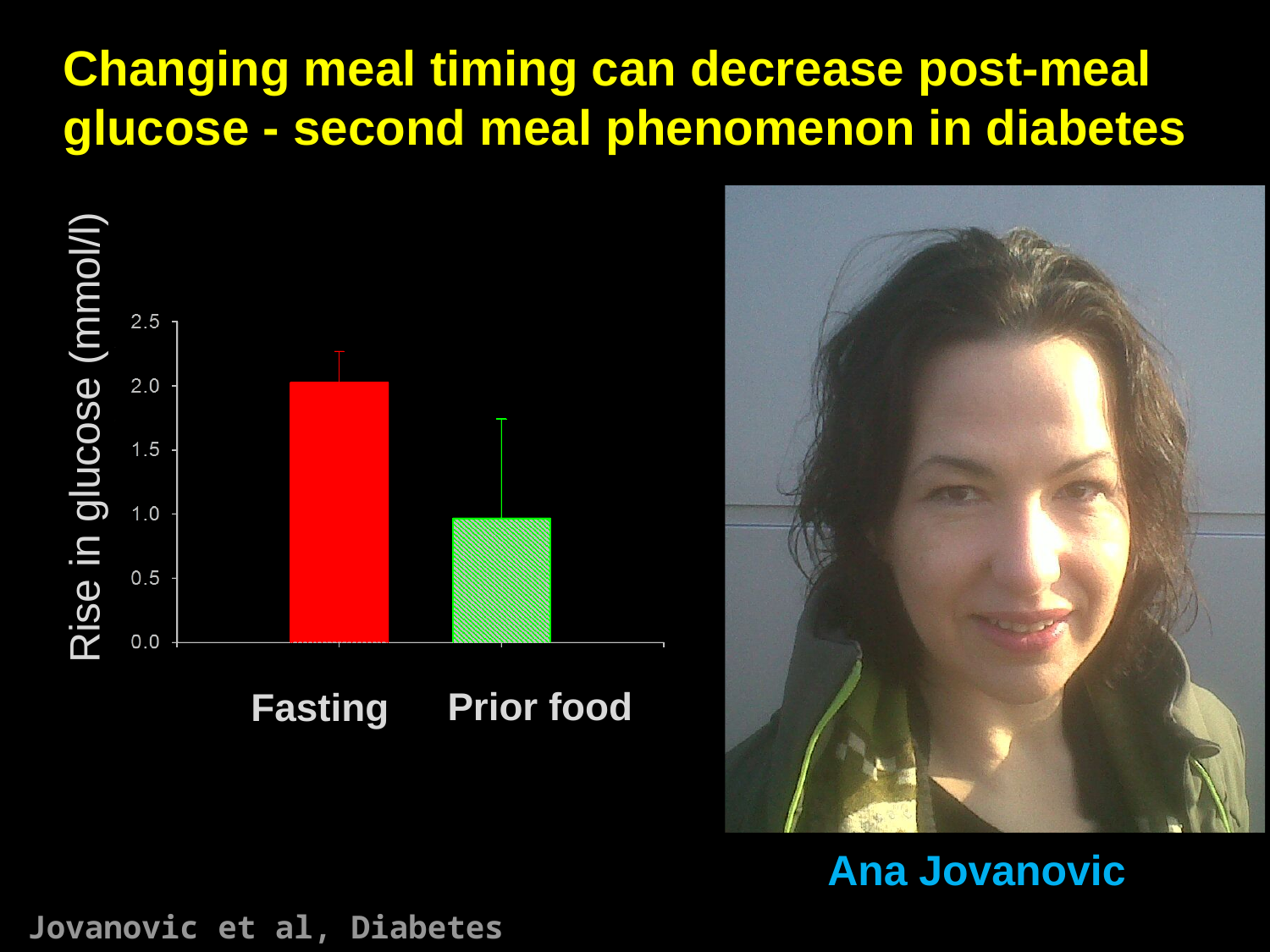

Changing meal timing can decrease post-meal glucose - second meal phenomenon in diabetes
Rise in glucose (mmol/l)
Prior food
Fasting
Ana Jovanovic
Jovanovic et al, Diabetes Care 2009

## Slide 57
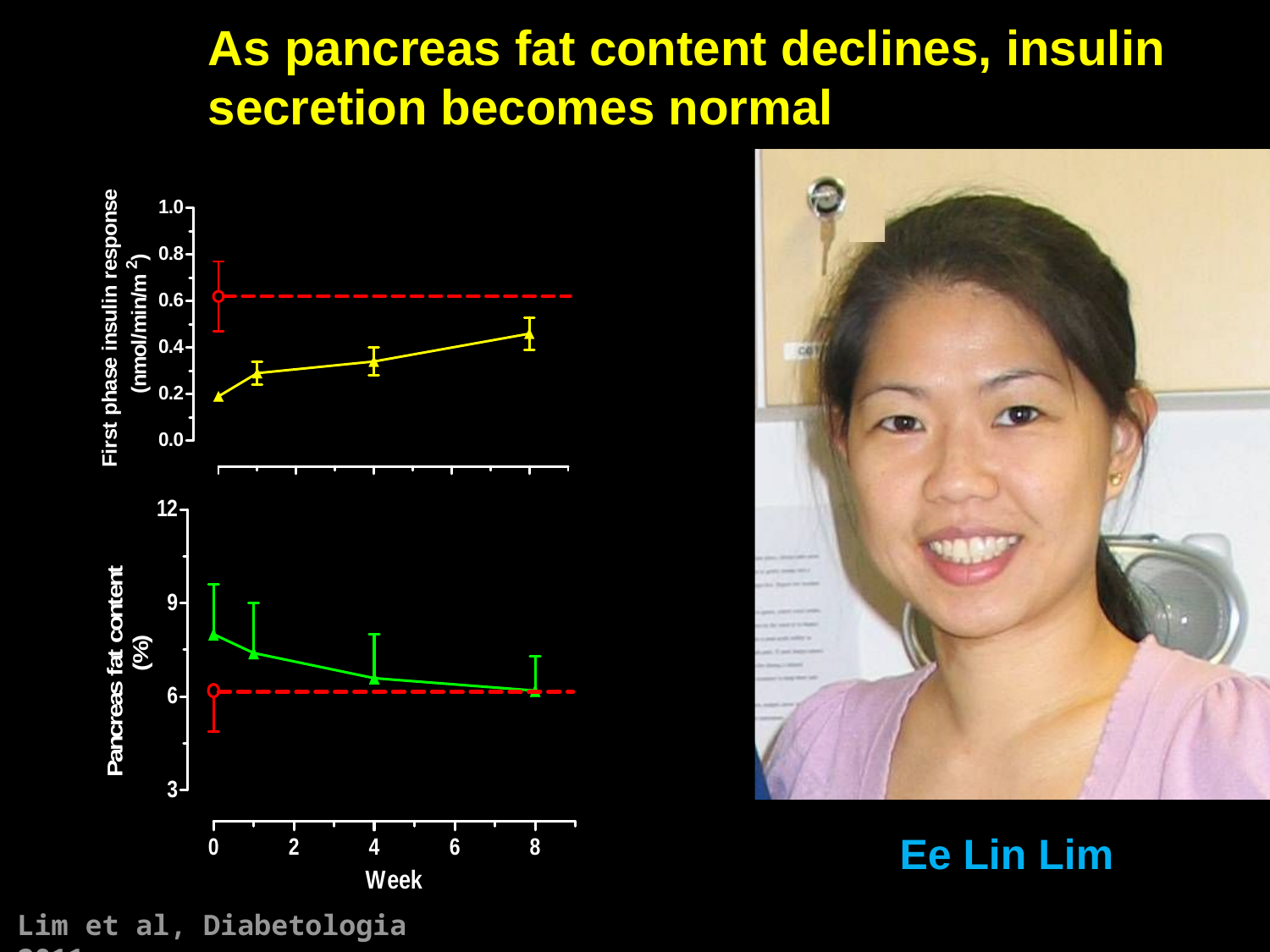

As pancreas fat content declines, insulin secretion becomes normal
Ee Lin Lim
Lim et al, Diabetologia 2011

## Slide 58
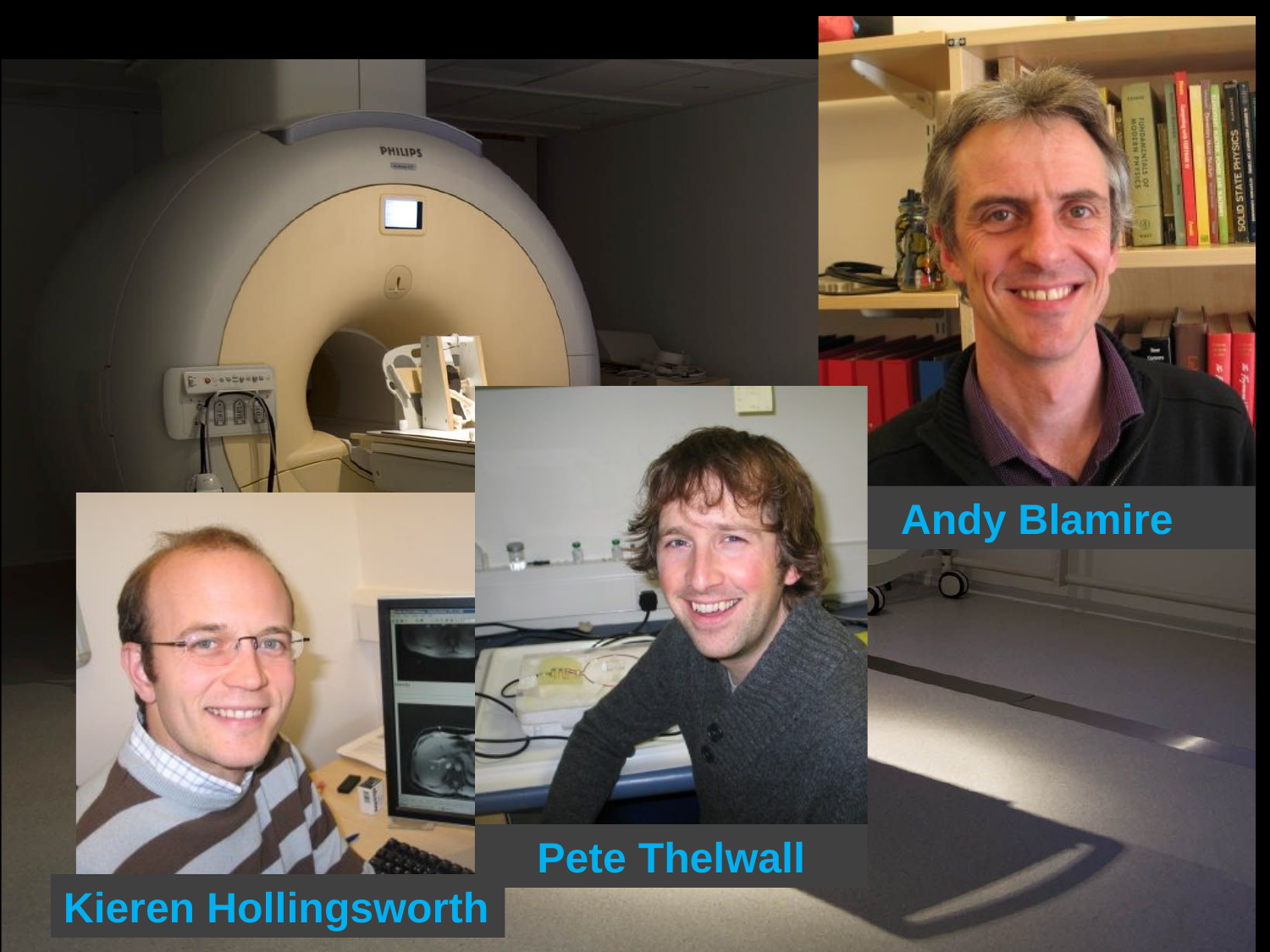

Andy Blamire
Pete Thelwall
Kieren Hollingsworth

## Slide 59
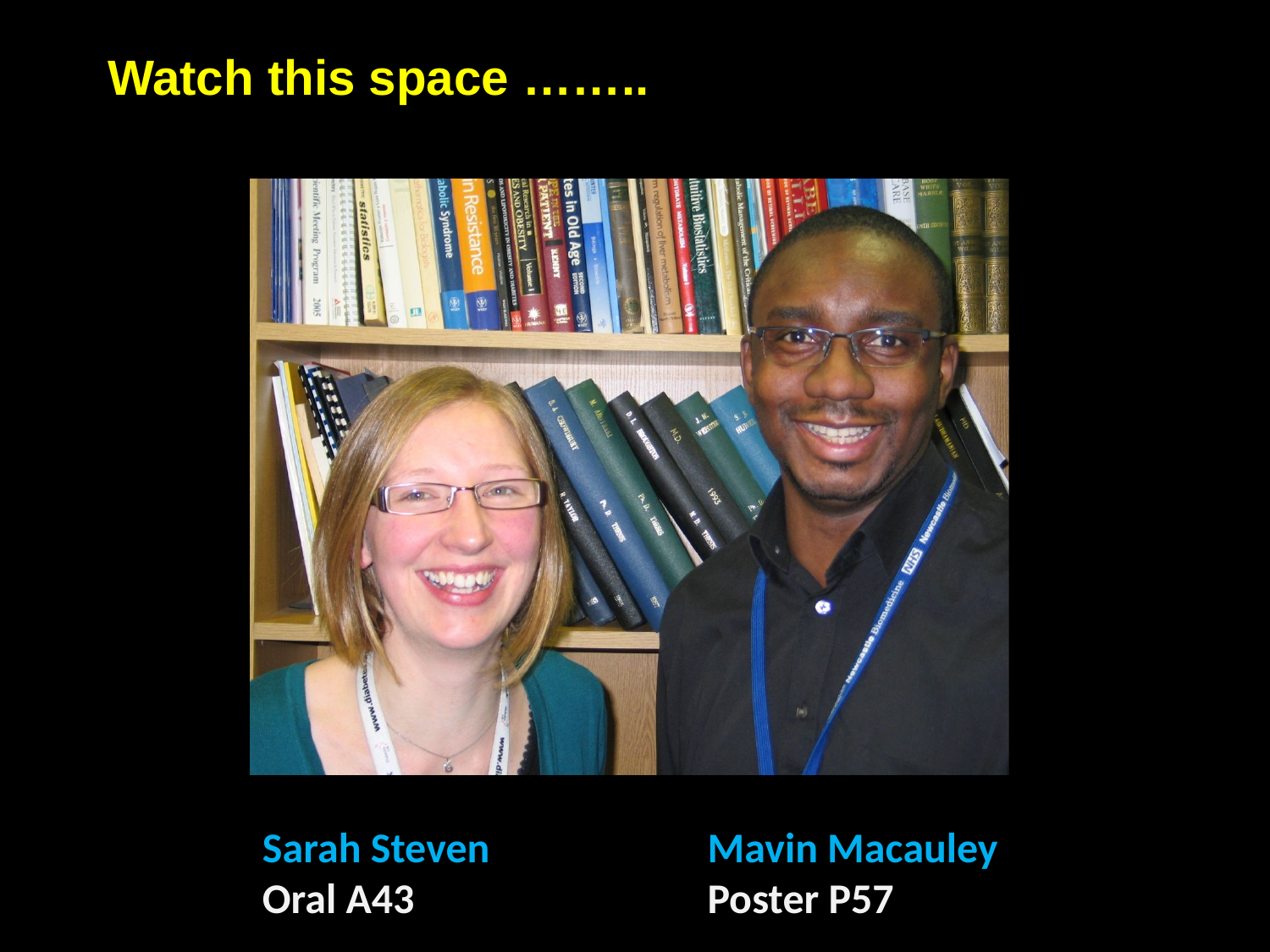

Watch this space ……..
Sarah Steven
Oral A43
Mavin Macauley
Poster P57

## Slide 60
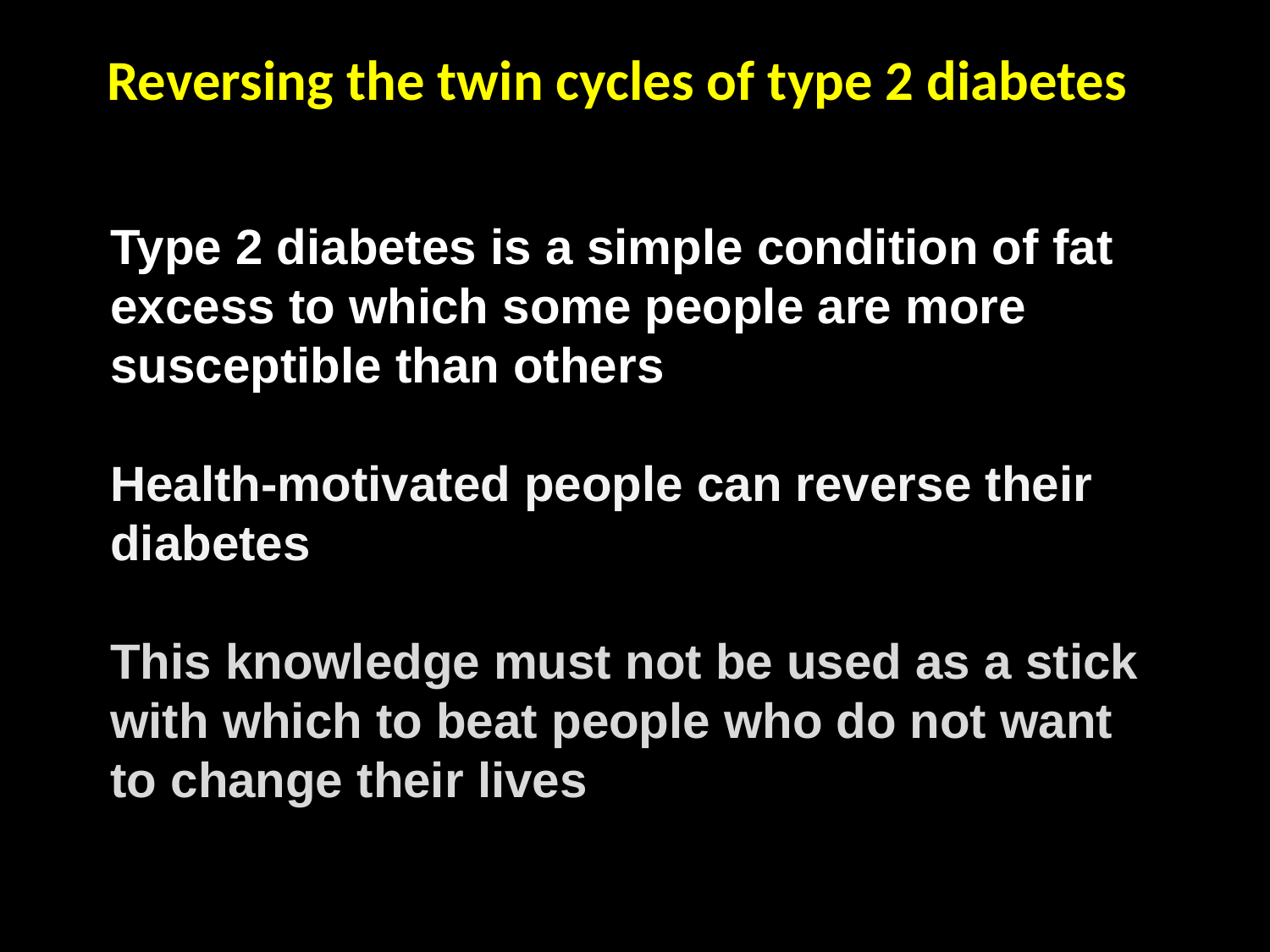

Reversing the twin cycles of type 2 diabetes
Type 2 diabetes is a simple condition of fat excess to which some people are more susceptible than others
Health-motivated people can reverse their diabetes
This knowledge must not be used as a stick with which to beat people who do not want to change their lives

## Slide 61
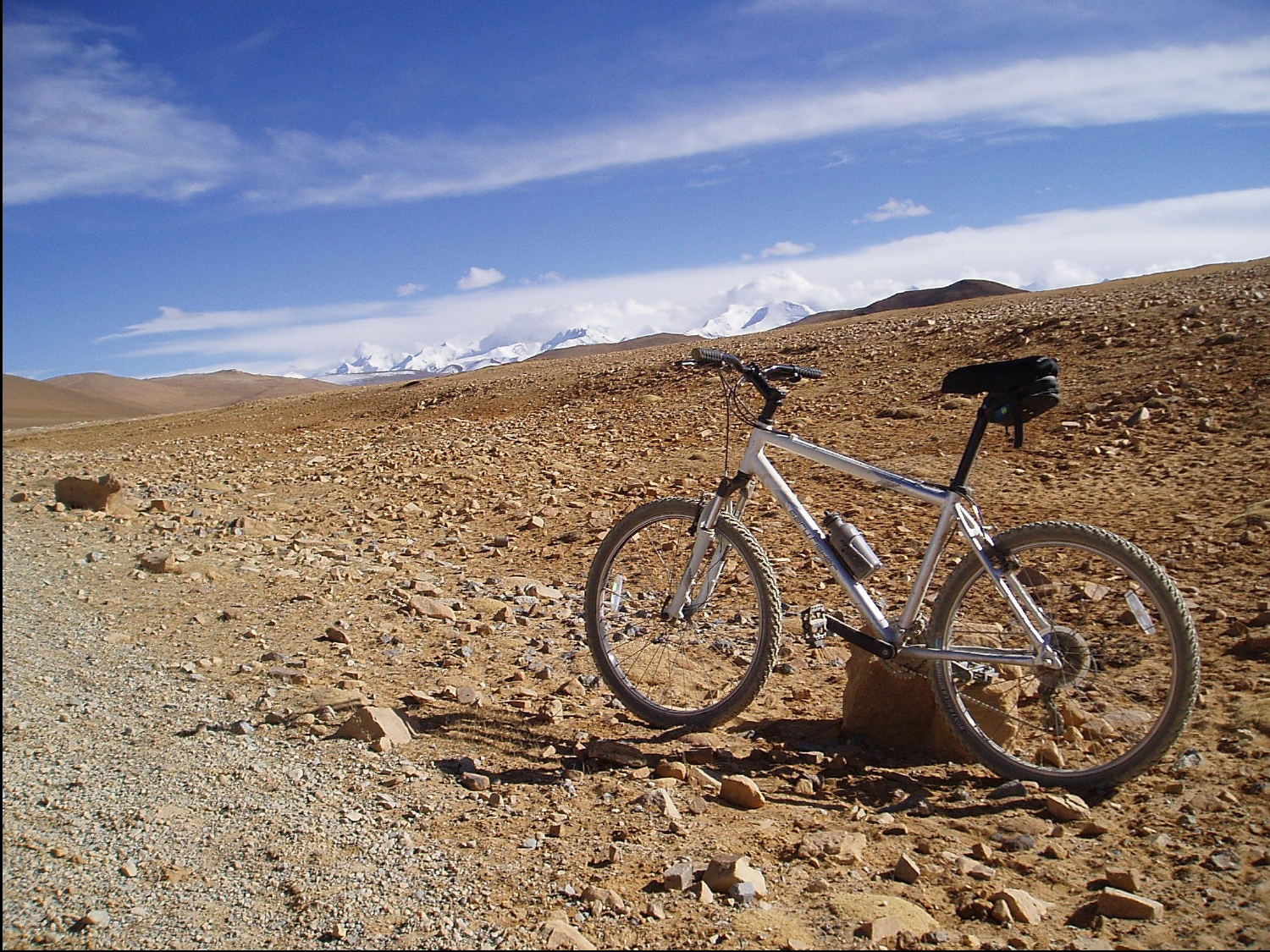

## Slide 62
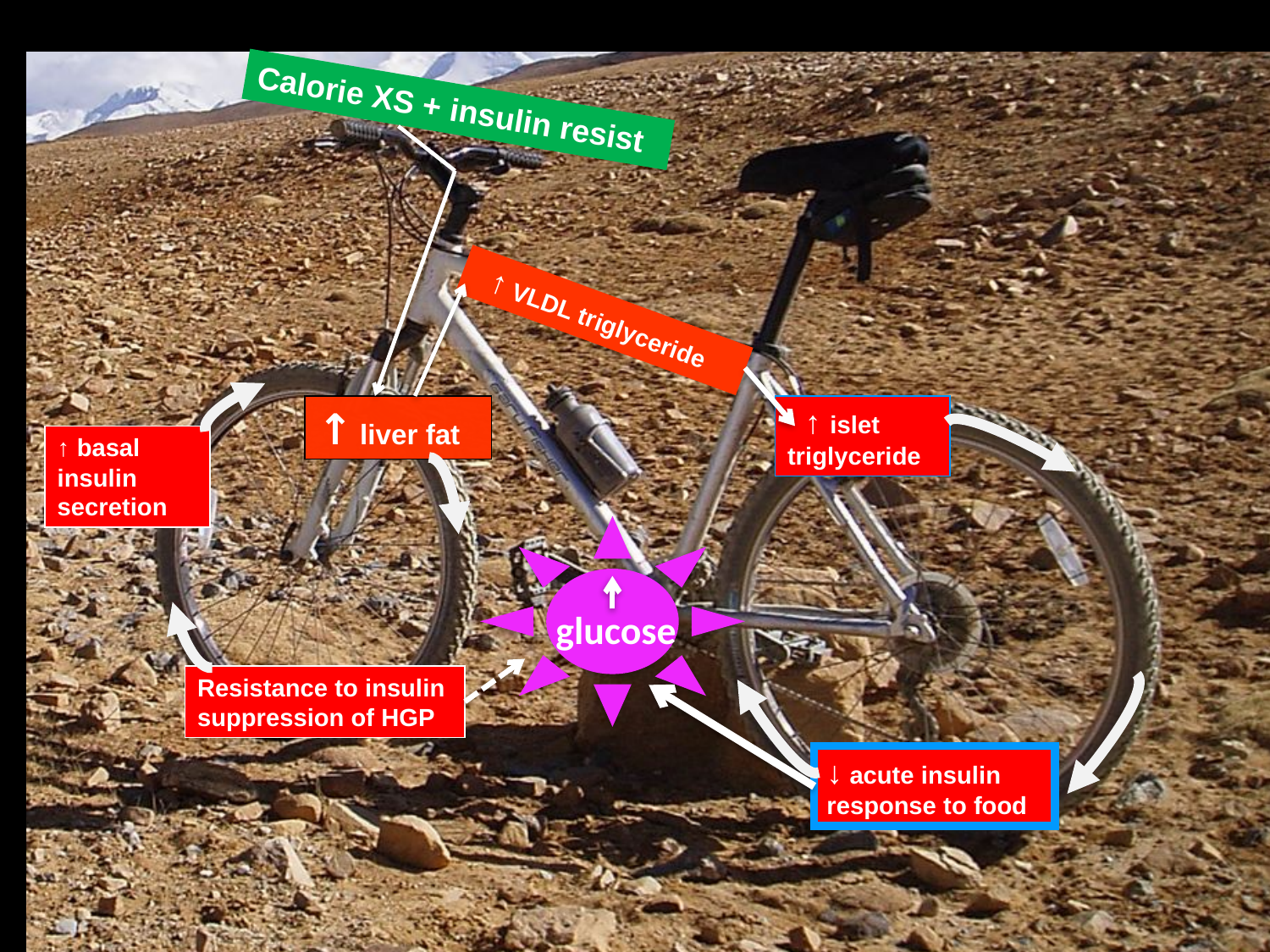

Calorie XS + insulin resist
 ↑ VLDL triglyceride
↑ liver fat
 ↑ islet triglyceride
↑ basal insulin secretion
glucose
Resistance to insulin suppression of HGP
↓ acute insulin response to food

## Slide 63
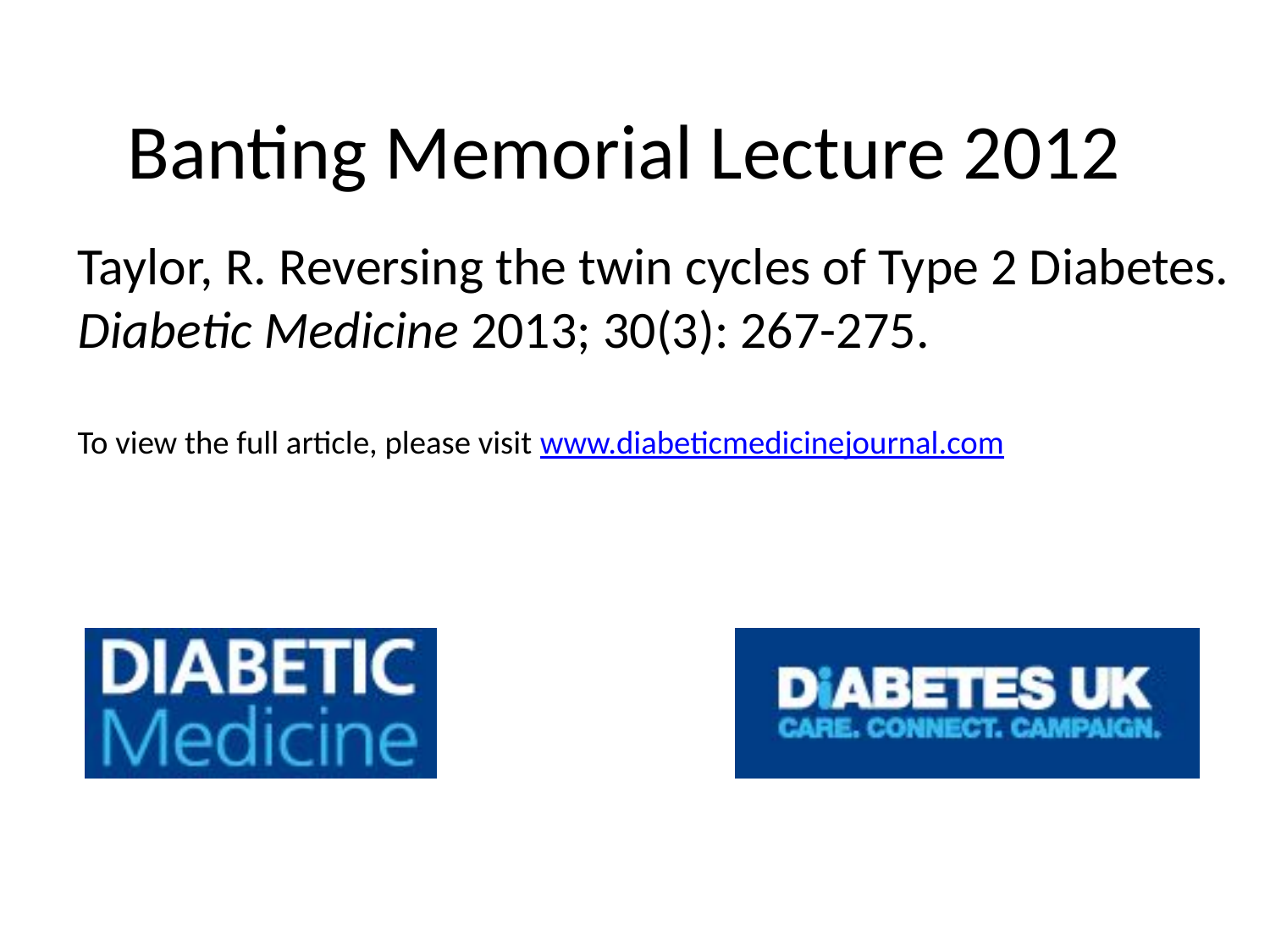

# Banting Memorial Lecture 2012
Taylor, R. Reversing the twin cycles of Type 2 Diabetes. Diabetic Medicine 2013; 30(3): 267-275.
To view the full article, please visit www.diabeticmedicinejournal.com
